# Green Extraction Techniques as Advanced Sample Preparation Approaches in Biological, Food, and Environmental Matrices: A Review

**DOI:** 10.3390/molecules27092953

**Published:** 2022-05-06

**Authors:** José S. Câmara, Rosa Perestrelo, Cristina V. Berenguer, Carolina F. P. Andrade, Telma M. Gomes, Basit Olayanju, Abuzar Kabir, Cristina M. R. Rocha, José António Teixeira, Jorge A. M. Pereira

**Affiliations:** 1 CQM—Centro de Química da Madeira, Natural Products Research Group, Universidade da Madeira, Campus Universitário da Penteada, 9020-105 Funchal, Portugal; rmp@staff.uma.pt (R.P.); cristina.berenguer@staff.uma.pt (C.V.B.); carolinafatimaandrade@hotmail.com (C.F.P.A.); telma_gomes_20@hotmail.com (T.M.G.); 2 Departamento de Química, Faculdade de Ciências Exatas e Engenharia, Universidade da Madeira, Campus da Penteada, 9020-105 Funchal, Portugal; 3 Department of Chemistry and Biochemistry, Florida International University, Miami, FL 33199, USA; olayanju.b@fiu.edu (B.O.); akabir@fiu.edu (A.K.); 4 Department of Pharmacy, Faculty of Allied Health Science, Daffodil International University, Dhaka 1207, Bangladesh; 5 CEB—Centre of Biological Engineering, Universidade do Minho, Campus de Gualtar, 4710-057 Braga, Portugal; cmrocha@ceb.uminho.pt (C.M.R.R.); jateixeira@deb.uminho.pt (J.A.T.); 6 LABBELS–Associate Laboratory, Universidade do Minho, Campus de Gualtar, 4710-057 Braga, Portugal

**Keywords:** green extraction techniques, microextraction techniques, sample preparation, biological samples, food samples, environmental samples

## Abstract

Green extraction techniques (GreETs) emerged in the last decade as greener and sustainable alternatives to classical sample preparation procedures aiming to improve the selectivity and sensitivity of analytical methods, simultaneously reducing the deleterious side effects of classical extraction techniques (CETs) for both the operator and the environment. The implementation of improved processes that overcome the main constraints of classical methods in terms of efficiency and ability to minimize or eliminate the use and generation of harmful substances will promote more efficient use of energy and resources in close association with the principles supporting the concept of green chemistry. The current review aims to update the state of the art of some cutting-edge GreETs developed and implemented in recent years focusing on the improvement of the main analytical features, practical aspects, and relevant applications in the biological, food, and environmental fields. Approaches to improve and accelerate the extraction efficiency and to lower solvent consumption, including sorbent-based techniques, such as solid-phase microextraction (SPME) and fabric-phase sorbent extraction (FPSE), and solvent-based techniques (μQuEChERS; micro quick, easy, cheap, effective, rugged, and safe), ultrasound-assisted extraction (UAE), and microwave-assisted extraction (MAE), in addition to supercritical fluid extraction (SFE) and pressurized solvent extraction (PSE), are highlighted.

## 1. Introduction

Over the last decades of the last century, technological improvements in chromatographic instruments boosted a remarkable evolution in the analytical chemistry field. Sophisticated configurations hyphenating fast and efficient chromatographic separations with powerful detection systems able to deliver unprecedented time of analysis and analytical performance become the forefront of this revolution where sample preparation was forgotten. For another decade, conventional processes, often involving large volumes of sample and organic solvents and laborious and many times cumbersome protocols prone to originate many experimental errors, continued to be used as standard procedures. Meanwhile, growing concerns with the environmental footprint and planet sustainability are promoting a green agenda affecting the most diverse human activities. The application of the green chemistry principles to analytical chemistry has been elegantly defined under the SIGNIFICANCE acronym [1]. Accordingly, the green analytical chemistry (GAC) envisages the simplest experimental layout involving minimal or no sample preparation and maximum integration of the analytical instruments used, preferentially in an automated way to limit operator intervention, energy consumption, and waste production. In this context, miniaturization of the sample extraction procedure, therefore decreasing sample and solvent requirements, as well as, wastes produced, was an obvious consequence of the GAC principles. This trend has fostered the development of a myriad of microextraction approaches, hereby considered green extraction (GreETs) approaches. These GreETs span almost all, if not all, fields of application, covering the microextraction of selected analytes from biological samples to food matrices or environmental matrices.

On this basis, this review will provide an updated overview of the most important and used green extraction approaches reported in the literature since 2016, their principles, advantages, limitations, and examples of application. Sorbent-based techniques, such as solid-phase microextraction (SPME), stir-bar sorptive extraction (SBSE), fabric-phase sorbent extraction (FPSE), and solvent-based techniques, including µQuEChERS (micro quick, easy, cheap, effective, rugged, and safe), single-drop microextraction (SDME), hollow-fiber liquid-phase microextraction (HF-LPME), and dispersive liquid–liquid microextraction (DLLME), have been considered. Additionally, the use of emerging green solvents such as ionic liquids (ILs) and deep eutectic solvents (DES) as an alternative to conventional solvents will be discussed. Finally, a brief overview of other promising green and sustainable approaches, such as pulsed electric-field-assisted extraction (PEFAE), supercritical fluid extraction (SFE), and subcritical water extraction (SWE), will also be provided.

## 2. Sample Preparation: A Key Step to Getting the Correct Data

**Figure 1:**
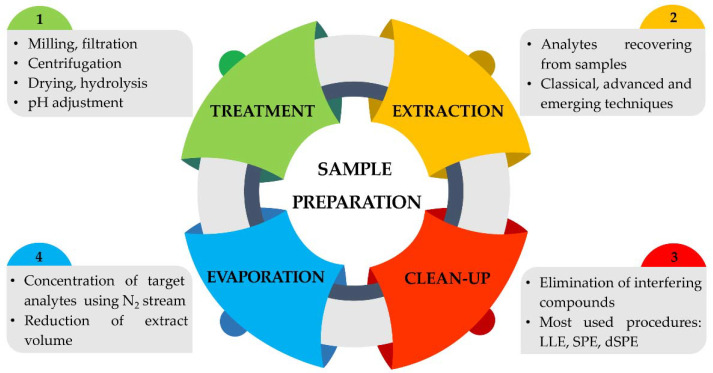
Different steps involved in sample preparation.

There has been an unprecedented growth in measurement techniques over the last few decades. Instrumentation, such as spectroscopy, microscopy, and chromatography, as well as microdevices and sensors, have undergone phenomenal developments. In contrast, the importance of sample treatment in the analytical layout seems to have been neglected. However, in the last decade, especially driven by the need of pharmaceutical and environmental industries, an exponential growth, and a rapid evolution in this industry, was observed. Some important steps in analytical chemistry to allow accurate, efficient, and fast determinations are commonly used in the sample preparation process, including, for example, extraction (recovering analytes from samples), clean-up (removal of compounds that can interfere with analysis), and solvent evaporation/concentration (concentration of analytes using an N_2_ stream), are shown in Figure 1. The procedures depend on the sample, the matrix, and the concentration level at which the analysis needs to be carried out.

Sample preparation is the source of about 30% of the experimental errors and of about 60% of the time spent on tasks in the analytical lab. For these reasons, independently of the high performance of the analytical instrument, the sampling procedure and the sample handling and pretreatment methodologies, following a carefully outlined process, are of utmost importance to acquire high-quality analytical results with high selectivity and low sensitivity limits and to ensure high accuracy and reproducibility. In addition, the selective isolation of the analytes of interest and the removal of interfering sample components are vital for eliminating the interferences and matrix effect and protecting the instrumental equipment from possible damages. However, as referred, these procedures were not always seen as key steps in the analytical process, and for that fact, the methodology followed in sample preparation did not receive the same attention as the analytical instrumentation, considered, until the last years, being the bottleneck of the whole analytical procedure. Indeed, the most widely used and commonly accepted classical extraction techniques (CETs) were liquid–liquid extraction (LLE), Soxhlet extraction, and solid-phase extraction (SPE). CETs, however, tend to be slow and labor intensive and use high amounts of hazardous organic solvents causing serious environmental concerns and present low extraction efficiency. Despite this reality, sample preparation techniques did not receive much attention until quite recently. In the last decades, to overcome the drawbacks of CETs, several novel microextraction techniques (Figure 1), which offer faster, cheaper, and “greener” pretreatment of complex samples; utilization of hazardous reagents; and less solvents with generation of less waste, maximizing the safety for operators and the environment, have been reported as alternatives to CETs.

These techniques, hereby designated as green extraction techniques (GreETs), exhibit attractive characteristics, such as simplicity, versatility, high extraction efficiency, and environmentally friendly profile, and have experienced increased development and implementation and stimulated significant progress in laboratory sample treatment. Some of them, due to their importance and growing application in the biological, food, and environmental fields, are highlighted below.

## 3. Green Extraction (GreETs) Techniques

The most important and used GreET meeting all green analytical chemistry (GAC) requirements, based on miniaturized SPE techniques, such as microextraction in packed syringe (MEPS), solid-phase microextraction (SPME) in direct (DI) and headspace modes (HS), stir-bar sorptive extraction (SBSE), and matrix solid-phase dispersion, in addition to liquid-phase extraction techniques, including single-drop microextraction (SDME), hollow-fiber liquid-phase microextraction (HF-LPME), dispersive liquid–liquid microextraction (DLLME), QuEChERS, solidification of floating organic drop microextraction (SFOME), and ultrasound-assisted back extraction (UABE), will be given more emphasis.

### 3.1. Miniaturized Sorbent-Based Techniques

**Figure 2:**
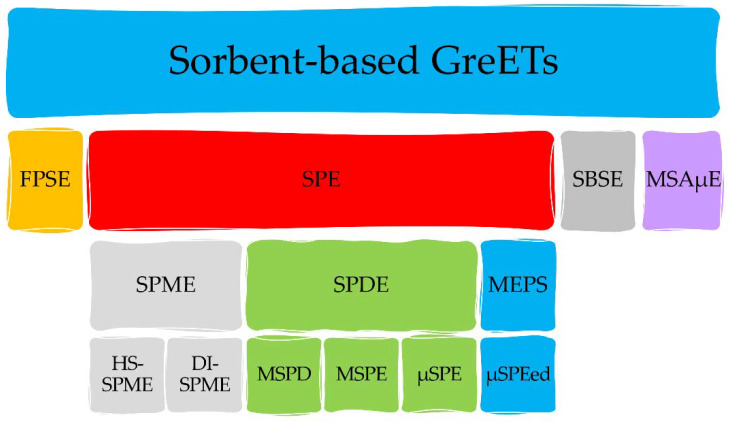
Different sorbent-based GreETs used in several fields of analysis. Legend: DI-SPME: solid-phase microextraction in direct immersion mode; FPSE: fabric-phase solvent extraction; GreETs: green extraction techniques; HS-SPME: solid-phase microextraction in headspace mode; MEPS: microextraction in packed sorbent; MSAμE: multisphere adsorptive microextraction; MSPD: matrix solid-phase dispersion; MSPE: magnetic solid-phase extraction; SBSE: stir-bar sorbent extraction; SPDE: solid-phase dynamic extraction; SPE: solid-phase extraction; SPME: solid-phase microextraction; μSPE: micro-solid-phase extraction.

SPE is one of the most used conventional extraction and preconcentration methods for the analysis of food, biological, and environmental samples [2,3]. However, this technique requires relatively large amounts of organic solvents and additional clean-up steps, which limits the automation, decreases sample throughput, and potentiates the contamination of the extracts [3]. Moreover, SPE uses large sample amounts, involving long extraction times [2,4]. Recently, new extraction methods were developed using modern techniques with less or no organic solvents to minimize environmental pollution and overcome the limitations of the conventional methods (Figure 2).

#### 3.1.1. Fabric-Phase Sorbent Extraction

Introduced in 2014 by Kabir and Furton [5], the solid-phase dynamic extraction (SPDE) format fabric-phase sorptive extraction (FPSE) is a fast, efficient, and versatile sample preparation approach by implementing a natural or synthetic permeable and flexible fabric (e.g., polyester, fiberglass, or cellulose) substrate to host a chemically coated sol–gel organic–inorganic hybrid sorbent in the form of an ultrathin coating. FPSE allows direct extraction of analytes without sample modification, thus minimizing/eliminating the sample pretreatment steps, which are considered the primary source of major analyte loss [6]. A strong covalent interaction between the fabric substrate and sol–gel contributes to improving the extraction efficiency medium, helping expose the FPSE to extreme chemical conditions without compromising the chemical/structural integrity of the microextraction device. The main disadvantages of FPSE are low sample capacity and extensive longer sample preparation time [7].

#### 3.1.2. Solid-Phase Extraction-Based Approaches

##### Solid-Phase Microextraction (SPME)

A key milestone in the development of microextraction techniques was first achieved by the seminal invention of solvent-free solid-phase microextraction, popularly known as SPME by Arthur and Pawliszyn in the early 1990s [8]. SPME is an equilibrium-based microextraction technique that involves the partitioning of the analytes from the sample solution into the sorbent coating of the SPME fiber owing to the intermolecular interaction or affinity for the sorbent material. Several configurations of SPME integrally optimize the volume of the extraction phase to improve the high surface-area-to-volume ratio of the extraction-phase coating. There are several geometries for SPME, such as planar, spherical, rod, and in-tube or cylinder [9]. The selection of the SPME geometry depends on the target analyte and matrix that will be analyzed. Usually, in the reduced diameter or length of the extraction phase, its higher surface-area-to-volume ratio can result in a smaller extraction period and higher recoveries [9].

SPME is a simple, fast, universal, sensitive, solventless, and economical technique for the preconcentration and sampling of analytes derived from various types of samples [3,10]. This technique combines extraction, enrichment, and sample injection into a single step. Other advantages of SPME are due to the reliability, sensitivity, and selectivity of this technique [4]. SPME allows the detection of semivolatile and nonvolatile compounds [10] and benefits from the constant development of new sorption coatings [3]. This procedure can be performed in different modes: (i) headspace SPME (HS-SPME) mode in which the analytes are adsorbed/absorbed from the gas phase in equilibrium with the samples (as the temperature is a parameter with a significant effect on the kinetics of the process, this is the most adequate for volatiles extraction); (ii) direct mode (DI) in which the SPME fiber is immersed directly into the bulk sample. In this case, the agitation is an important experimental parameter to facilitate the transport of the analytes from the solution to the fiber. (iii) In the third mode, membrane extraction, the extraction of less volatile compounds is facilitated by the use of a protected membrane.

##### Microextraction in Packed Sorbent (MEPS)

**Figure 3:**
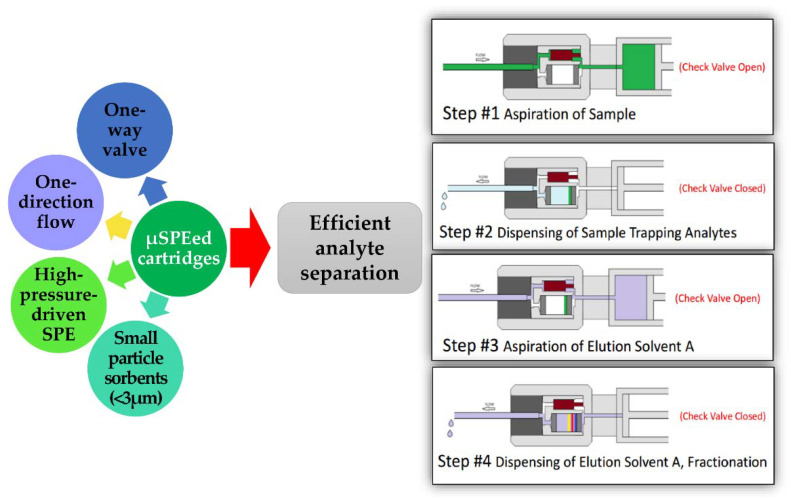
Advantages and schematic overview of µSPEed.

MEPS emerged as a greener alternative to the conventional SPE, consisting of a sample pretreatment technique based on the miniaturization of SPE. This technique uses the same sorbents as SPE but is considered more advantageous since sorbent integration into a liquid-handling syringe results in low void volumes, making sample manipulations easy [4]. MEPS can be applied to smaller samples and requires shorter sample preparation times and lower solvent volumes [2]. Moreover, MEPS can be performed online in a fully automated way using the same syringe for sample extraction and extract injection into the analytical instrument [4]. A typical MEPS application comprises sorbent conditioning, sample loading, washing, and analyte elution. Contrary to SPE, the two-direction flow potential in MEPS provides the duplication of each step and satisfactory sorbent conditioning, enhanced sample–sorbent interaction, sample loading, and improved analyte elution. The elution and washing steps can be performed with 20–50 µL of organic solvent, and 1–4 mg of reused sorbent material is sufficient to extract a target analyte with high efficiency [11]. More recently, µSPEed has been proposed. It represents an advance with respect to MEPS because it has a unidirectional valve that corresponds to a flow in one direction, in addition to the high pressure conferred by the small diameter of the sorbent particles. In this case, the analytes retained in the adsorbent are not altered by the aspiration of solvents, as is the case with MEPS, which results in more efficient extractions. The most remarkable improvement over MEPS procedure is the direct flow through the sorbent bed; therefore, the analytes retained in the sorbent bed are not disturbed by the solvent aspiration as it occurs with MEPS. Moreover, the high pressure and the single direction contribute to obtain more efficient extractions of the target analytes. Figure 3 represents a schematic overview and the most important aspects of µSPEed.

##### Solid-Phase Dispersion Extraction (SPDE)

In SPDE, the microparticles are dispersed in the solution (liquid sample) until the equilibrium between the two phases is reached. The most popular formats are the matrix solid-phase dispersion extraction (MSPD), magnetic solid-phase extraction (MSPE), and micro-solid-phase extraction (µSPE).

MSPD is an efficient and generic technique for the isolation of a wide range of drugs, pesticides, naturally occurring constituents, and other compounds from a wide variety of complex plant and animal samples. According to Barker [12], the sample is dispersed over the surface of the bonded-phase support material, producing, through hydrophobic and hydrophilic interactions of the various components, a unique mixed-character phase for conducting target analyte isolation [12].

MSPE is based on the use of sorbent materials, such as magnetic nanoparticles, carbon hemimicelles, and molecular imprinted polymers [13]. C18 functionalized magnetic nanoparticles (MNP) are used for preconcentration or cleanup of moderate and nonpolar polar pesticides due to the absence of internal diffusion resistance, the excellent absorption capacity of the target analytes, and the high surface-to-volume ratio [14]. The main advantage of MSPE is that the sorbent is composed of MNPs, often NPs of the most diverse chemistries and geometries, that can be easily recovered from a solution by a simple spin or centrifugation process.

The pipette-tip SPE is the simplest format of µSPE in which the sorbent is placed in a tip and extraction is handled by using a pipette, widely used in preclinical and clinical development programs in addition to the study of metabolomics, genomics, and proteomics. SPE tips, such as the MonoTip^®^, NuTip^®^, and ZipTip^®^, can be used for the purification of peptides or proteins that, using affinity and metal chelation, can be successfully selectively isolated [15].

#### 3.1.3. Stir-Bar Sorbent Extraction (SBSE)

Stir-bar sorbent extraction (SBSE) was introduced by Baltussen, Sandra, David, and Cramers in 1999 as an alternative to SPME and became one of the most powerful microextraction and preconcentration techniques for the enrichment of volatile analytes from aqueous samples due to its simplicity, robustness, cost-effectiveness, and environmental friendliness. After that, its applications have been extended to the analysis of nonvolatile analytes and solid and liquid samples.

This extraction procedure is based on a magnetic stir (0.5–1 mm thickness) coated with polydimethylsiloxane, a nonpolymeric phase used as an extraction phase of target analytes through hydrophilic interaction. SBSE consists of two steps: extraction and desorption. Related to extraction, the coated stir bar can act in immersion mode (immersed in the sample solution) or in headspace mode (stir bar is exposed in the gas phase above the liquid or solid sample). After extraction, the target analytes adsorbed in the stir bar are desorbed by thermal desorption, followed by analysis in a chromatographic system (e.g., GC, HPLC, and CE) [11,16].

Despite that the SBSE principle is similar to SPME, SBSE exhibits higher sensitivity, recovery, and extraction efficiency. This is due to the larger amount of coated phase in SBSE, which is 50–250 times higher than the SPME fiber, making SBSE more suitable to analyze trace levels in complex matrices. On the other hand, a special interface is required for thermal desorption in gas chromatography (GC), and lower recoveries are obtained for target analytes with a logarithm of octanol–water partitioning coefficient (log Ko/w) lower than 3 [11,16].

#### 3.1.4. Multisphere Adsorptive Microextraction (MSAμE)

A new adsorptive microextraction (AμE) technique was proposed by Nogueira et al. [17,18], which represents a great alternative for the enrichment of a wide range of polar analytes at trace levels in aqueous media, selecting appropriate sorbent phases. The new AμE approach can be used through different analytical devices presenting suitable geometry, where specific sorbents are simply sustained through sticking-based technologies. Usually, the sorbent is physically embedded on the substrate and put in the aqueous media. The solution is stirred using a stir bar or vortexed. Since most of the polar targets are nonvolatile and some of them have thermolabile properties, liquid desorption (LD), followed by HPLC is certainly the following combination of choice for analytical purposes. AμE can appear in two geometrical configurations, namely, bar adsorptive microextraction (BAμE) and multisphere adsorptive microextraction (MSAμE). Nevertheless, previous experimental data [17,18] showed that MSAµE devices present much better stability compared with BAμE, especially when they are exposed to an aggressive sample matrix because, in this case, thermal supporting promotes much higher robustness from the fixation point of view. The MSAμE showed several advantages, namely, high recovery for polar analytes, easiness to prepare, economicalness, and selectiveness, as sorbent can be selected based on the target analyte of interest. Nevertheless, the main drawback is the device’s stability as should be evaluated on a case-by-case basis.

### 3.2. Miniaturized LPE-Based Techniques

**Figure 4:**
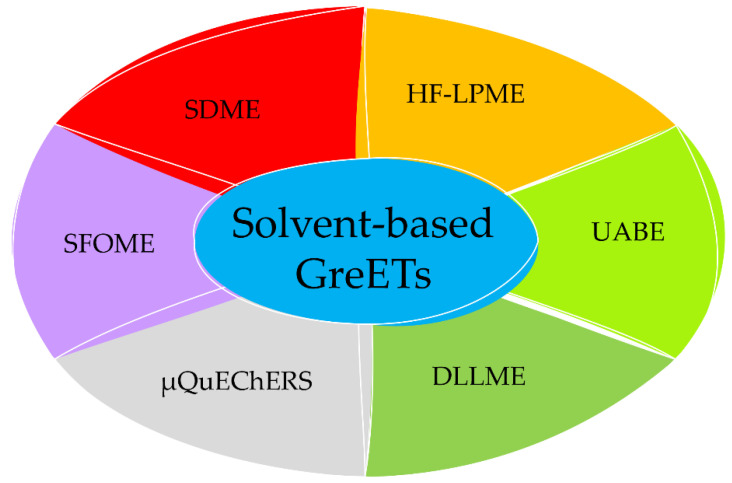
Different liquid-based GreETs used in several fields of analysis. Legend: DLLME: dispersive liquid–liquid microextraction; GreETs: green extraction techniques; HF-LPME: hollow fiber liquid-phase microextraction, SDME: single-drop microextraction; SFOME: solidification of floating organic drop microextraction; UABE: ultrasound-assisted back extraction; µQuEChERS: micro-QuEChERS.

In addition to sorbent- or solid-based GreETs, several microextraction approaches involving a sorbent phase in a liquid state have been developed in the last decades. Similar to the SPE-based techniques, the major shortcomings of the conventional liquid–liquid extraction technique (LLE), such as emulsion formation, long preparation time, noncompliance with GAC due to the usage of a high volume of toxic organic solvents, and inevitability of solvent evaporation and sample reconstitution, have triggered research into the miniaturized and greener version of LLE. In contrast to SPME, miniaturized LPE techniques include solvent-based extraction techniques that use microliters of organic solvent to accomplish the selective isolation, preconcentration of the analytes, and clean-up of the sample (Figure 4).

#### 3.2.1. Single-Drop Microextraction (SDME)

**Figure 5:**
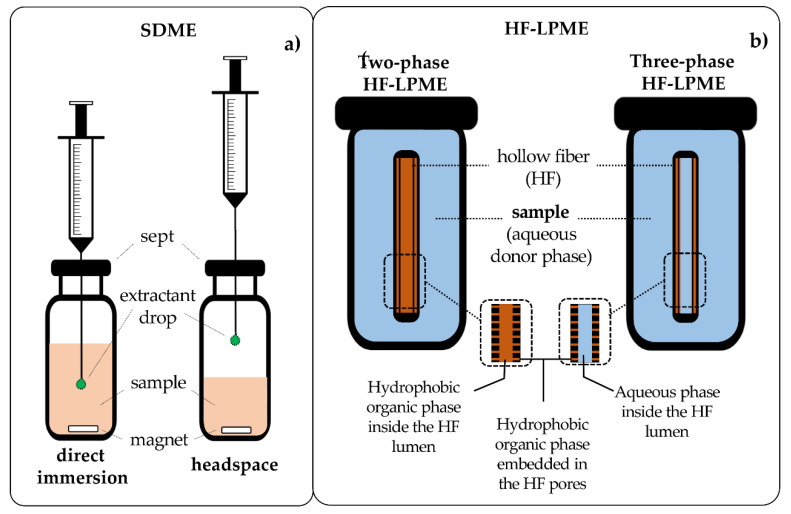
Schematic representation of direct immersion and headspace modes of single-drop microextraction (SDME) (**a**) and two- and three-phase modes of hollow fiber liquid-phase microextraction (HF-LPME) (**b**).

The first to be invented for the series of solvent-based microextraction techniques was SDME. SDME is a nearly solvent-free, quick, inexpensive, and easy-to-operate extraction technique. It can be used to highly enrich analytes in a relatively short time and uses simple laboratory equipment, which considerably lowers the cost of analysis [4,10]. This approach implies that a single drop of an extraction solvent is employed for the isolation of the analyte. It was introduced in the mid-1990s by Liu and Dasgupta [19], which used a drop of water to extract ammonia and sulfur dioxide before the spectrophotometric analysis. SDME is based on the principle of the partitioning of the analytes from the sample solution to the extraction solvent with or without mechanical aid. As presented in Figure 5a, SDME can be operated in different modes: direct immersion is employed mostly for nonvolatile analytes, being the extraction solvent immersed in the liquid sample from which the analytes are transferred, subsequently followed by the withdrawal of the drop before instrumental injection; the headspace mode (HS-SDME) is tailored for the isolation of volatile compounds [20]; the bubble-in-drop (BID-SDME) introduced by Williams et al. [21] was designed to enlarge the droplet surface area; continuous-flow microextraction (CFME) proposed by Liu and Lee [22] was designed to increase the contact area between the analyte and the extraction solvent; or the drop-to-drop liquid–liquid microextraction was developed by Wijethunga et al. [23] in which the sample volume required for analysis is reduced. Automation has also been established with SDME coupled with electrothermal atomic absorption spectrometry for the quantitation of Cr (VI) in natural water samples [24].

#### 3.2.2. Hollow-Fiber Liquid-Phase Microextraction (HF-LPME)

Pedersen-Bjergaard and Rasmussen [25] first developed the hollow fiber LPME coupled with capillary electrophoresis for methamphetamine in biological samples, such as urine and plasma. It is another mode of solvent-based microextraction technique that is premised on the transfer of the target compounds from the sample (donor) solution via a supported liquid membrane to the acceptor phase. Since its introduction, it has gained wide popularity for the analysis of a wide range of analytes in environmental samples [26], biological samples [27,28], and food samples [29,30]. HF-LPME could be operated in two different modes, a two-phase system and a three-phase system (Figure 5b). Although the two modes share a similarity in principle in that they involve the partitioning of the analytes from the sample (donor phase) solution to other phases (acceptor phase), few lines of demarcation can be observed. In two-phase systems, the analytes are transferred from the aqueous phase to the organic acceptor phase based on their affinity for them. In turn, the three-phase mode involves partitioning from the aqueous donor across the organic solid support liquid membranes (SLMs) into the aqueous acceptor phase in the lumen of the hollow fiber [26]. This results in several advantages that accompany automation, including a lower number of operators to recruit, reduced chemical use, and accelerated analysis time, just to mention a few. Automated HF-LPME has been applied for the analysis of pharmaceutical drugs [31].

#### 3.2.3. Dispersive Liquid–Liquid Microextraction (DLLME)

DLLME has also gained wide popularity over the years. DLLME offers several advantages, including small sample volume, high extraction efficiency, low consumption of solvents, high enrichment factor, good repeatability, and high recovery. Furthermore, this technique is simple and uses small amounts of extraction solvents, and the equilibration between the aqueous phase and extracting solvent is fast [32]. Ultrasounds can be applied to disperse the extraction solvent in the sample, avoiding the reduction of the analyte’s partition coefficient between water and the extracting solvent [32]. As a result of efforts to minimize errors incurred in analysis due to intermittent human intervention and to increase the efficiency of the overall process, studies detailing the automation of the LPE techniques have also been reported, for instance, the online sequential injection (SI) DLLME for the isolation and preconcentration of copper and lead using a series of reagents including methanol as disperser solvent mixed with 2.0% (*v*/*v*) xylene as the extraction solvent and 0.3% (*m*/*v*) ammonium diethyldithiophosphate as the complexing agent. The solvent mixture was merged with the aqueous sample, and 300 μL isobutyl methyl ketone was used to elute the complex of the analytes before the injection into the nebulizer of the flame atomic absorption spectrometry (FAAS) [33]. A similar study involving a modification was reported for the quantitative analysis of cadmium and lead in natural water samples [34].

#### 3.2.4. QuEChERS

At the beginning of this century, Anastassiades, et al. [35] proposed an innovative sample preparation approach with attractive characteristics, a quick, easy, cheap, effective, rugged, and safe (QuEChERS) method for the quantitative measurement of pesticide residues in vegetables and fruits. It is a two-stage process of solid–liquid partitioning with a salting-out effect and a dispersive solid-phase extraction (dSPE). The extraction of the target analytes occurs in the first stage, when a mixture of salts is dispersed in the matrix and mixes thoroughly with an organic phase, often acetonitrile, till an equilibrium is reached. This is followed by a clean-up step (dSPE) using a different combination of porous sorbents and salts according to the matrix interferences that should be removed. Since its invention, QuEChERS has been applied for the analysis of a wide spectrum of analytes in different sample matrices, such as fluoxetine and carbamazepine in benthic invertebrates (*Potamopyrgus antipodarum* and *Valvata piscinalis*) [36], pharmaceuticals and personal care products in sewage and surface waters [37], sulfonamide residues in milk samples [38], or BPA in human urine [39]. More recently, several improvements to the original procedure were reported, notably, its miniaturization applied in different fields of research [40] (additional reports available in Tables S1–S3).

#### 3.2.5. Solidification of Floating Organic Drop Microextraction (SFOME)

The SFOME method was introduced by Khalili Zanjani et al. [41] using polycyclic aromatic hydrocarbons as model compounds. In this technique, the collection of the analytes in a microdrop of an organic extraction solvent under agitation is achieved by the solidification of the suspended microdrop organic layer in ice. The solidified microdrop is allowed to melt before it is injected into the instrument for quantitative assessment. The notable characteristic of the extraction solvent, peculiar for this procedure, is its low melting point usually in the range of 10–30 °C. The use of a little amount of organic solvent indicates the compliance of this simple method with the green analytical chemistry requirements (GAC), and it has been popularly employed either individually or in conjunction with other extraction methods for the analysis of contaminants in environmental [42], food [43], and biological [44] samples. The use of SFOME is not limited to the extraction of organic compounds as it has been deployed for the isolation of inorganic metallic ions, such as lead [45].

#### 3.2.6. UABE

Ultrasound-assisted back extraction (UABE) is another LPE-based extraction method that has been used in tandem with other sample preparation strategies, such as cloud point extraction, for the analysis of brominated flame retardants in water samples (BFRs) [46], heterocyclic aromatic amines in natural water samples [47], and DLLME for the isolation of suvorexant in urine samples [48]. The use of UABE was reported to be a necessity when the extracts contain much extraction solvent that is not compatible with the analytical instrument. Zhou, Gao, Zhang, Li, and Li [46] developed a method for the quantitative determination of BFRs in water samples using a cloud point extraction coupled with UABE before injection into the inlet of HPLC–MS/MS. An amount of 400 μL of an aqueous solution of Triton X-114 and 0.5 M ammonium acetate was added to 40 mL of water sample, which was thoroughly mixed and centrifuged at 5000 rpm for 3 min. After the aqueous phase was decanted, 200 μL of acetonitrile and 2 mL of isooctane were added to the surfactant-rich component and sonicated for 5 min. The isooctane layer was allowed to dry with the aid of N_2_ flow in a new centrifuge tube, while the residue was reconstituted in methanol (50 μL) after which it was injected into the HPLC system. This method gave a limit of detection of 0.3 to 3.0 ng L^−1^ and a recovery of 8.7%–54.7%.

### 3.3. Emergent Green Solvents: Ionic Liquids (ILs), DES, and NADES

ILs have evolved as potential replacements for conventional solvents over the years. They are low-melting organic salts with a combination of an organic cation and an organic or inorganic anion, occurring in the liquid state at a temperature below 100 °C. The striking features of ILs, such as negligible vapor pressure, enhanced synthesis route, fewer by-products, thermal stability, and high hydrophobicity, are factors establishing them as green solvents as they are more environmentally friendly than conventional solvents [49]. Added to the advantages of ILs is the ability to modify functionalities, which enhances the selectivity and specificity of the target molecules [50]. ILs have been shown to improve selectivity and extraction efficiency when used in tandem with a metal organic framework (MOF). For instance, [51] employed imidazole-based ILs as a guest material with Zr-MOFs for the preconcentration of sulfonamides in a water sample in a dSPE-HPLC-DAD method. A limit of detection below 0.03 μg L^−1^ and enrichment factors greater than 270 were obtained. Despite the promising usefulness of ILs in the field of separation science, their notable drawback is that not all ILs are nonvolatile, nonflammable, and stable in air and water as originally considered. In fact, many ILs are volatile, flammable, unstable, and even toxic, particularly to aquatic beings [52]. For this reason, deep eutectic solvents (DES) have emerged as safer alternatives, exhibiting higher stability and lower costs and toxicity [52]. DES are formed by combining different hydrogen bond acceptors and donors, and their classification as ILs is not consensual, mainly because they share more differences than similarities (reviewed in [52]). Among those differences, it is important to highlight in the context of this review that DES are less hazardous and more stable and biodegradable than IL [52]. A specific subclass of DES, composed of components of natural origin, NADES, will be the ultimate green solvent that can be used. For their greener profile and possibilities to fine-tune extraction properties by combining different ILs and, more recently, DES and NADES, the use of these innovative extraction solvents in the most diverse extraction formats is growing exponentially and constitutes one of the forefronts in sample extraction. An exhaustive list of applications using ILs, DES, and NADES is available in Tables S1–S3.

### 3.4. Other Advanced Extraction Techniques

Other advanced extraction techniques may act at different levels (alone or in combination), including, but not limited to, breaking the overall matrix structure or cell wall, allowing easier penetration of selective solvents with an affinity for the target analytes, fastening mass transfer and extraction kinetics; using more selective and cost-effective extraction solvents, increasing analyte solubility, increasing safety, and decreasing environmental impact (e.g., by changing the type of solvent, decreasing the chemicals needed, reducing energy consumption, or reducing wastes generation). Pulsed electric-field-assisted extraction (PEFAE) is a nonthermal technology that has been primarily applied to disintegrate cells and cellular tissues in food processing and extraction processes. It makes use of very intense electrical pulses with a very short duration. The main objective is to disrupt cell walls and tissues and increase cell permeability without heating the target samples (and analytes), thus increasing extraction efficiency. As the duration of the pulses is designed to avoid thermal effects, it can be used with heat-sensitive compounds. Other electric-field-based approaches are also available. For instance, using moderate electric fields for longer times, combining thermal and nonthermal electric effects, may also be an effective way of extracting the target analytes. This process is also known as ohmic heating. Heat is generated inside the material through the Joule effect, and the matrix is heated almost instantaneously and evenly. Simultaneously, a limited electroporation effect is also expected. It is applicable when heat is needed to achieve an efficient extraction of the target analytes, increasing extraction efficiencies and reducing thermal degradation. Furthermore, energy efficiency in ohmic-heating-based processes is significantly higher than in traditional heating processes [53]. Electric technologies may be used complementary to other more traditional approaches to increase their efficiency and selectivity and decrease the time of sample preparation [54]. Applications of electric fields can be found both in analytes’ extraction and in samples’ concentration/purification. One example, described by Xu and coworkers [55], is the application of electric fields to enhance SPE extraction of contaminating compounds (tricyclic antidepressants) in environmental waters before their identification by GC–MS [55]. Electro-enhanced solid-phase microextraction can be found applied to several other matrices and analytes, including phthalate esters and bisphenol A from blood and seawater [56] or fluoroquinolones in eggs [57]. Electroextraction of analytes across aqueous–organic phase boundaries has also been described, and membrane-based processes coupled with electric fields are also quite common in the literature [58]. Using greener non-petroleum-based solvents and tuning their properties to increase their efficiency and selectivity by using subcritical or supercritical temperatures and pressures is also an interesting approach to reduce or remove the use of toxic and/or environmental impacting solvents in the extraction step. For instance, when considering analytes with moderate polarity and low thermal sensitivity, subcritical water extraction (SWE) may be considered a viable option. Pressurized solvent extraction (PSE) consists of a liquid–liquid extraction technique where the solvent is used at temperatures higher than its “normal” boiling point (at atmospheric pressure) in pressurized systems. The pressure is kept at values above the boiling point at the selected temperature, but under the critical point, allowing to keep the fluid in the liquid state. Higher temperatures allow increasing the solubility of the analytes and the transfer rate, thus improving extraction efficiencies. Further, viscosity decreases, and the high pressures involved may facilitate the solvent penetration into the matrix from which the analytes are being extracted. SWE is a particular case of pressurized liquid extraction, using water as solvent. Water is a solvent with unique properties in these conditions, not present in other solvents. Besides the above-mentioned advantages, the dielectric constant of water decreases when temperature increases, thus increasing its affinity for less polar analytes. Though this decrease is limited, it is possible to use subcritical water as a greener replacer for “intermediate” polarity organic solvents, such as methanol or ethanol. Further, it is possible to tune water properties to meet the desired affinity for the target analyte by selecting the most appropriate temperature. On top of that, the ionization constant of water also increases, thus liberating more ions, H^+^ and OH^−^, that may work as a catalyst to break down the matrix, thus improving the solvent accessibility to the analyte [59]. On the other side, for thermolabile nonpolar analytes, supercritical carbon dioxide extraction (SCE) is a relevant option, avoiding the use of solvents, such as hexane. Supercritical fluids have mixed properties between liquids and gases, facilitating extraction processes: diffusion, viscosity, and surface tension similar to gases and density and solvation power as liquids. In the particular case of CO_2_, the critical temperature is close to room temperature (31 ℃), and working under supercritical conditions is possible at relatively low temperatures. The solvent’s low polarity makes it ideal for nonpolar compounds. However, SCE of more polar compounds is possible using a chemical modifier or a cosolvent (such as ethanol), though decreasing the process greenness. Further evaporation or concentration steps are not needed: resuming the atmospheric conditions turns the solvent back into gas that can either return to the environment or be pressurized again to be reused while purifying/concentrating the analyte’s sample.

Greener processes tend to use greener solvents with lower environmental impact. Bio-based solvents using renewable sources or water are considered solvents with a lower environmental footprint. However, the utmost target should be using direct analytical methods not requiring reagents or solvents [60]. When the sample’s pretreatment is unavoidable, alternatives to reduce or use no solvent at all in the preparation step should be considered [61]. In this context, sample treatments such as simple pressing or extrusion, instant controlled pressure drop, PEFs, or microwaves applied directly in the matrix being analyzed, without adding extra solvents, may cause membrane or cellular structure ruptures enough to free intracellular or structural fluids containing the compounds to be analyzed.

In Table 1 are described the advantages and disadvantages of the most common GreETs used in the analysis of biological, food, and environmental samples.

## 4. High-Resolution Analytical Techniques

Liquid chromatography (LC) has a great benefit on the efficiency of separating complex matrices, but it is not appropriate to achieve structural information of the target analytes. In this sense, LC combined with mass spectrometry (MS) or MS/MS is certainly the most common analytical approach in the analysis of a diversity of target analytes in environmental, clinical, and food matrices, since it provides higher selectivity, mainly when isomeric mixtures were analyzed. HPLC combined with a traditional detector such as ultraviolet (UV) [62], photodiode array detector (PDA) [63], and fluorescence detector (FLD) [64] have been applied in the determination of several target analytes in environmental, clinical, and food matrices. The benefits of these traditional systems are economical, more accessible in common laboratories, efficient, faster, and easy to use. Despite the lower sensitivity attained using these traditional detectors, excellent results related to validation parameters were achieved, namely, low limit of detection (few µg/L), good accuracy (recoveries higher than 70%), and intra- and interday precisions with relative standard deviation (RSD) lower than 20%. Currently, UV, PDA (DAD), and FLD detectors have been substituted by MS and/or MS/MS detectors since they provide high selectivity, sensitivity (low LODs), and ability to provide information related to molecular mass and structural proprieties. Liquid chromatography–tandem mass spectrometry (LC–MS/MS) [65] is becoming a promising analytical approach to analyzing complex matrices due to its high separation resolution, high sensitivity (low LODs), and capacity to identify compounds and does not require any derivatization step before the analysis. Nevertheless, compared with the traditional systems, LC–MS/MS showed several drawbacks, such as the complexity of the operation, expensiveness, and strong matrix effects that promote in many cases signal suppression or enhancement.

The atmospheric pressure ionization of MS has electrospray ionization (ESI) and atmospheric pressure chemical ionization (APCI). The introduction of ESI and APCI overcame the limitation of previous interfaces by evaporating the mobile phase during the ionization process. This, combined with the orthogonal spray interface, provided a means to stop possibly interfering nonvolatile compounds, such as salts, buffers, and detergents, from entering the MS. For many target analytes, ESI provides high sensitivity, being more used than APCI [65]. APCI is more appropriate for the analysis of nonpolar analytes and volatile organic compounds (VOCs). In addition, the major benefits of the application of ESI for quantitative LC–MS are the production of protonated or deprotonated molecules with slight fragmentation, optimal selection of precursor ions, and maximizing sensitivity, the matrix effect being its main drawback.

Single quadrupole, triple quadrupole (QQQ-MS), ion trap (IT), time of flight (TOF), and quadrupole–time of flight (Q–TOF) are the most common MSs used in tandem mass spectrometry (MS/MS). The IT, TOF, and Q–TOF mass spectrometers usually are used for structural elucidation and the identification of unknown compounds. Nevertheless, achieving structural information of unknown compounds requires higher purity of matrices. More sophisticated analytical approaches, such as ultra-performance liquid chromatography–tandem mass spectrometry (UHPLC–MS/MS) [66], have been recently used in the analysis of compounds in environmental, clinical, and food matrices. UHPLC–MS/MS compared with HPLC–MS/MS provides high pressures, narrow peaks, high chromatographic separation, and lower analysis time and solvent volumes. Moreover, LC coupled to high-resolution mass spectrometry (HRMS) was used for direct determination of glyphosate and its metabolite aminomethylphosphonic acid (AMPA) in human urine by combining cold-induced phase separation (CIPS) with hydrophilic pipette tip solid-phase extraction (PT-SPE) [67]. LC–HRMS compared with LC–MS offers screening for targeted, suspect, and nontargeted analysis in a single run, producing high-resolution accurate masses, their isotopic patterns, and MS2 spectra included in online databases.

Gas chromatography (GC) coupled with a flame ionization detector (FID) [68], MS [69], or MS/MS [70] has also been used in minor extension, when compared with LC–MS and LC–MS/MS, in the analysis of environmental, clinical, and food matrices. This fact could be explained by the derivatization process required for the analysis of some target analytes in GC analysis to promote the volatility and decrease the polarity of the analytes, as well as the time of analysis.

Other analytical approaches have been used in the analysis of environmental, clinical, and food matrices, such as flame atomic absorption spectrometry (FAAS) [71], inductively coupled plasma (ICP) combined with mass spectrometry (MS) [72], or optical emission spectrometry (OES) [73]. Additional details about these and other examples are available in Tables S1–S3, covering the clinical, food, and environmental fields, respectively.

## 5. Applications of Green Extraction Techniques to Different Fields

**Figure 6:**
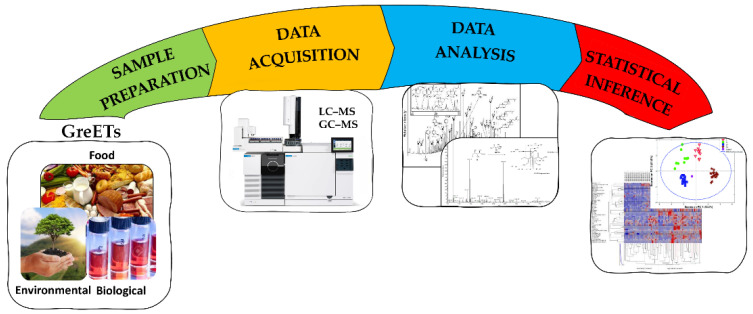
Different steps involved in sample preparation. GreETs: green extraction techniques.

As discussed in the previous sections, the use of GreETs spans a wide range of applications, covering the most diverse type of samples, from biofluids to environmental matrices and all type of foods (an exhaustive list of applications reported in the literature since 2016 is available in the Supplementary Material). In modern analytical layouts and to fulfill GAC requirements, the analysis that follows the sample preparation using GreETs should employ fast and efficient analytical instruments able to acquire huge amounts of data. As a consequence, powerful data processing and statistical analysis procedures will be required to produce consistent results (Figure 6).

### 5.1. Biological Samples

The application of GreETs to the clinical field has increased consistently since the beginning of the century [74]. This mostly includes body fluid samples containing lower-molecular-mass organic molecules, less than 500 g/mol, comprising drug analytes, metabolites, environmental exposure contaminants, poisons, tissues, and endogenous substances [74]. These biological samples present great complexity and moderate-to-high levels of protein, thus requiring robust sample preparation approaches able to simplify and isolate the target analytes from the matrix [75]. As discussed in more detail in the previous sections, traditional sample preparation methods are not particularly tailored for clinical applications because they are time-consuming and require various steps and extensive clean-up before analysis. In contrast, most GreETs require low sample amounts, very low or no solvent at all, and simple, fast, and user-friendly systems that can be easily automated [75]. These advantages made SPME, µSPE, MEPS, MSPE, just to name a few GreETs, particularly suitable to process biological samples. Moreover, they also allow spanning a wide range of analytes with different properties, such as drugs for clinical and forensic toxicology assays, pharmacokinetic studies, biochemical analysis, pharmaceuticals, in vivo applications, and metabolomics [75]. SPME and its different formats are particularly efficient in this field of application because they often require minimum sample pretreatment and can be easily coupled to analytical instruments (e.g., CG and LC), providing an enhanced extraction capacity and simultaneous quantification of different compounds with overall sensitivity. This includes the simultaneous identification of drugs of abuse (e.g., amphetamines, barbiturates, methadone), psychoactive substances, pharmaceuticals (e.g., antidepressants, antiepileptic agents, steroids, anorectic agents, corticosteroids, anaesthetics), substances that affect the adrenergic system, nonsteroidal anti-inflammatory substances, and so forth [76]. Examples of such applications are available as Supplementary Material (Table S1). Among the different biological matrices, microextraction of urine samples has the advantage of minimum processing, often not requiring any centrifugation or filtration before extraction. This minimizes sample handling and improves method precision. Additionally, it is suitable for a wide range of sample volumes, including volumes as small as 50 μL, and even for sampling when the volume is not accurately known. Diverse types of GreETs using urine are available in the literature, SPME, µSPE, and MEPS being the most often reported [75] (Table S1). The use of GreETs with blood sampling is also advantageous, particularly when this allows the elimination of blood-withdrawal steps from the analytical workflow, as with SPME. GreET usage also reduces the risk of analyte degradation and matrix changes due to enzymatic conversion, as well as fast sample collection and clean-up. Different examples of applications involving blood sampling using GreETs can be found in the literature, such as VOCs (SPME [77]), polycyclic aromatic hydrocarbons (PAHs, pipette-tip SPE [78]), Ni and Pb (µSPE [79]), opiates (MEPS [70]), and antidepressants (FPSE [80]). SPME has also been reported in in vivo assays with biological matrices like tissues. This can be performed with a removed tissue portion (ex vivo), direct in vivo measurement, exposing the BioSPME needle to the tissue or even inserting the probes directly into the tissue. Regarding this, Musteata [81] observed that microdialysis and SPME were not only appropriate for tissue sampling but also complementary to each other for in vivo sampling and ex situ analysis. By using this approach, the probe extracts only a slight fraction of the free analyte, minimizing disturbances of chemical equilibrium and allowing multiple measurements of analyte concentrations under physiological conditions. Moreover, the accurate determination of analyte concentration is unaffected by the sample volume. Finally, the technique is open to miniaturization, allowing its application within small living systems, sample storage and transportation, and easy coupling to portable instrumentation [75]. An example of such an approach was reported by Cudjoe, et al. [82], which used SPME to monitor neurotransmitter changes in the striatum of a rat brain after dosing antidepressants, variations in serotonin concentrations due to deep-brain stimulations, and distribution of pharmaceuticals in the striatal region and cortex. This elegant experiment shows that SPME can also be very useful in metabolomics assays, particularly at the initial stage of biomarker discovery in medical diagnosis. It is also very relevant to the quantification of different compounds simultaneously, which enables the simultaneous monitoring of drugs in complex treatments. This is possible because GreETs coupling with chromatographic methods, as shown in Section 4, can be easily achieved, allowing the analysis of a whole pharmacopoeia of drugs, such as anticancer, antibiotic, antidepressant, analgesic, anti-inflammatory, steroid, and neurotransmitter drugs. This can help to provide earlier detection of the disease, which is imperative for a successful clinical treatment, especially in some oncologic diseases, where an early diagnosis is crucial for the survival of the patient without suffering severe impacts on health and life quality. FPSE is a very promising GreET having a key advantage regarding other microextraction approaches, allowing a direct analyte extraction with no sample modification [6]. Since its introduction in 2014, many examples of applications involving biological samples have been reported in the literature, such as the cow and human breast milk sample clean-up for screening bisphenol A and residual dental restorative material [83]; the simultaneous monitoring of inflammatory bowel disease treatment drugs [84] and anticancer drugs [85] in whole blood, plasma, and urine; or the assessment of radiation exposure [86] (Table S1). The use of magnetic nanoparticles as microextraction sorbents in MSPE also results in a very simple and efficient extraction procedure because the sorbent can be tailored to extract specific analytes, and the sorbent-retained analyte complex can be easily recovered from the solution using a magnetic field or magnet [87]. MSPE has been used to extract different drugs from urine, such as nonsteroidal anti-inflammatory drugs (NSAIDs) [88], methadone [89], pseudoephedrine [90], fluoxetine [91], and statins [92], as well as antiepileptic drugs [93] or ibuprofen [94] from plasma (Table S1). GreETs involving liquid-phase sorbents, such as DLLME, are also often reported in the literature. This format, mostly assisted by ultrasounds (UA-DLLME), allows the usage of a myriad of extraction solvents, and consequently, the repertoire of applications is very broad. Mabrouk et al. [95], for instance, used UA-DLLME to extract three gliflozins (antidiabetic drugs) from plasma.

### 5.2. Food Samples

Food analysis is of great importance since ingestion of a growing number of compounds intentionally or not added to food can represent a risk to our health. However, beyond food safety, consumers are also more aware of the nutritional value of food and are also interested in its composition, particularly regarding the presence of bioactive compounds. For these reasons, efficient methodologies for the identification and quantification of all these analytes are required. Accordingly, GreETs have been used in the sample preparation procedures of different food matrices to extract and preconcentrate target analytes to a sufficient level to allow their analysis [96]. The µSPE technique, for instance, has been used in the determination of aflatoxins [96], pesticides [62], trace metals [73], and pollutants, such as bisphenol A [97] and PAHs [68], in a variety of food products. Additionally, it aided in the identification and quantification of rosmarinic acid in medicinal plants [98] and vitamin D3 in bovine milk [99]. MEPS is another GreET that has been employed in the analysis of foodstuffs, including the identification of herbicides in rice [100], insecticides in drinking water [101], pesticides in apple juice and coffee [102], antibiotics [103] and steroids [104] in milk, parabens in vegetable oil [105], PAHs in apple [106], caffeine in drinks [107], and polyphenols in baby food [108]. SPME has been widely used to study the volatile composition of several foods, including walnut oils [109], *hongeo* [110], melon [111], and dairy products [112]. Moreover, this technique has also been used to determine the composition of specific analytes, such as the x-ray induced markers 2-dodecylcyclobutanone and 2-tetradecylcyclobutanone in irradiated dairy products [113], the contaminants 1,4-dioxane and 1,2,3-trichloropropane [114], acrylamide [115], organophosphorus pesticides [116], phthalates [117], synthetic phenolic antioxidants [118], and xanthines [119]. MSPD has been reported in the literature for the extraction of flavonoids [120], polyphenols [121], mangiferin, and hyperoside in mango-processing waste [122], ergosterol in edible fungi [123], and pharmacologically active substances in microalgae [124]. This methodology has also been applied for pesticide [125] and sulfonylurea herbicide [126] extraction in several food matrices. MSPE allowed the extraction of trace metals in food products [127] (additional examples available in Table S2). Moreover, studies have shown that this technique can be used for the determination of acrylamide [78,79], bisphenols [80], PAHs [128], plant growth regulators [129], and caffeine [130]. FPSE is another GreET that has been shown to be very useful for the determination of several classes of pesticides in foods [96]. Other analytes studied using this technique include bisphenol A [131], oligomers [132], PAHs [64], steroid hormone residues [133], and tetracycline residues [134]. DLLME has been vastly applied for the determination of trace metals [71] (additional examples available in Table S2), pesticides [96], chloramphenicol [135], and nonsteroidal anti-inflammatory drugs [136] in different foods. μQuEChERS was employed in the extraction of several analytes from foods, ranging from pesticide residues in wine [137] and PAHs in coffee and tea [138] to polyphenols in baby food [139] and pyrrolizidine alkaloids in oregano [66]. The application of SDME was proved to allow the determination of unfavorable compounds and elements in foods, such as drug metabolites [140], acrylamide [141], ammonia [142], ethyl carbamate [143], formaldehyde [144], tartrazine [145], and Cu(II) [146]. Similarly to SDME, SFOME can be used for the detection of trace metals [147], as well as of β-lactam antibiotic residues [148] and organochlorine pesticides [149]. PEAE has been applied for the extraction of different bioactive compounds [63], including phenolic compounds [43], carotenoids [150], procyanidins [151], and sulforaphane [152]. The use of SFE has been used for the extraction of several antioxidant and antibacterial compounds from feijoa leaf [153], fatty acids and oils from Indian almonds [154], oleoresins from industrial food waste [155], and polar lipid fraction from blackberry and passion fruits [156]. Additionally, SFE was employed for the extraction of phytochemicals from *Terminalia chebula* pulp [157]. Finally, SWE is a technique largely applied to the extraction of several classes of bioactive compounds, including anthocyanins [158], fatty acids [159], hesperidin and narirutin [160], phenolic compounds [161], and scopoletin, alizarin, and rutin [162]. The extraction of antioxidant protein hydrolysates from shellfish waste [163] and pectic polysaccharides from apple pomace has been also previously accomplished by SWE [164].

### 5.3. Environmental Samples

Most environmental samples have complex matrix compositions and involve the determination of trace and ultra-trace analytes [3]. For instance, the determination of PAHs in water samples or pesticide analysis is challenging due to their very low concentrations [2,3]. This requires efficient clean-up and enrichment procedures before the analytes’ analysis [2]. MEPS seems to be tailored for these requirements and has been applied in the analysis of benzene, phenol and their derivates [165], diazinon [166], La^3+^ and Tb^3+^ [167], organophosphorus pesticides [168] in water samples, fipronil and fluazuron residues in wastewater [169], and PAHs in the most diverse samples (see Table S3), including Antarctic snow [170], and in the detection of phthalates in tap and river water [171]. SPME is eventually one of the most used sample extraction procedures and has been applied for the detection of different pesticides in water [172] (additional examples in Table S3), microplastic in coral reef invertebrates [173], PAHs in rainwater [174], and volatile organic compounds (VOCs) in wastewater [69]. Molecularly imprinted polymers (MIPs) have also been employed in the extraction of polychlorinated aromatic compounds from environmental samples. Some applications include the use of MIPs in the analysis of 2-chlorophenol [175], 2,4-dichlorophenoxyacetic acid [176], and endosulfans [177] in water samples and in the determination of organochlorine pesticides in environmental samples [178]. This methodology has also been reported in the preparation of soil samples to increase the extraction efficiency of triazine herbicides [179]. Multisphere adsorptive microextraction (MSAμ) has been applied in the extraction of caffeine, acetaminophen [18], pharmaceuticals, sexual steroid hormones, and antibiotics [17] in water samples. QuEChERS is known as the Swiss knife of extraction. Its µQuEChERS version is even more greener and includes applications such as the detection of insecticides in guttation fluids [65], pesticides in arthropods and gastropods [180], and VOCs in zebrafish [181].

LPME techniques, such as SDME and SLLME, use small volumes of organic solvents to extract the analytes [4]. SDME has gained a lot of interest in the last few years and is mostly used for the determination of trace analysis in environmental matrices, including Cu(II) in tap and seawater [146], PAHs in tap water [182], ranitidine in wastewater [183], and V(V) in water samples [184]. DLLME is another efficient microextraction procedure, and its ultrasound-assisted (UA) DLLME variation has been adopted in several environmental matrices for the analysis of aromatic amines [185], Cd [186], Cr [187], dyes [188], herbicides [189], polybrominated biphenyls [190], pyrethroid insecticides [191], and tetracycline [192] in water samples. SFE was applied to environmental matrices for the analysis of Ag in electronic waste [193], petroleum biomarkers in tar balls and crude oils [194], petroleum hydrocarbons in soil [195], and solanesol in tobacco residues [196]. In turn, SWE has been successfully used for the extraction of Co, Li, and Mn in spent lithium-ion batteries [197], crude oil in soil [198], oil shale in mines [199], and VOCs in sewage sludge [200]. An exhaustive list of GreETs involving environmental samples is available in Table S3.

## 6. Final Remarks

The sample preparation, including the extraction process, is one of the most important steps for any analysis. It is the step that determines the quality of the measurements of the target analytes, and so it should be critically considered. In recent years, a rapid transition from solvent-based extractions and solid sorbent-based extractions to miniaturized formats promoted a substantial reduction of the solvent consumption. Being simpler, faster, more economical, and user-friendly than CETs, SPE techniques have become more popular. In turn, this has boosted a continuous improvement of innovative techniques respecting the green analytical chemistry principles, such as, FPSE, SPME, SPDE, MEPS, SBSE, and MSAμE. However, there are always opportunities for improvement, and future research should be directed to, for instance, innovative sorbents/nanosorbents able to further improve retention efficiency, loading capacity, and selectivity. Also noticeable will be the use of artificial intelligence, including microfluidics and smartphones, to boost the automation of extraction procedures and the use of alternative detectors. Another challenging aspect in this field in the near future is the integration of the whole analytical procedure in injection loops able to protect operators from harmful solvents and significantly reduce their use.

## Figures and Tables

**Table 1:** Advantages and disadvantages of some GreETs commonly used in the analysis of biological, food, and environmental matrices.

Extraction Procedure	Advantages	Disadvantages
SPME	Alternative to SPE A limited number of steps Reduced sample amount Reuse of the polymeric phase Environmentally friendly Short extraction time	Potential contamination of the SPME needle
µSPE	Alternative of LLE Simplicity of automation Suitable for large scale Low sorbent Low solvent volume	Requires stirring Possibility of low recoveries
MEPS	Low solvent volume Low sample amount Fast and easy to use Economical Fully automated for online procedure	Requires a wide range of optimization steps
MSPE	Environmentally friendly A limited number of steps Low amount of sorbent material Reuse of sorbent material Short extraction time	Requires vortex/shaker/magnetic stirrerSelection of suitable sorbent
MSDP	Environmentally friendly A limited number of steps Quick Simple	Requires anhydrous sorbents activated at high temperatures
FPSE	Efficient Fast extraction Low volume of solvents High preconcentration factor	Low sorbent capacity Long sample preparation time
DLLME	Economical High recovery Low sample amount Low extraction time Low solvent volume	Low selectivity Requires centrifugation
SFOME	Environmentally friendly High enrichment factor economical Low volume of solvents Simplicity of automation	Requires a wide range of optimization steps
μQuEChERS	Economical Efficient clean-up by dSPE Low solvent consumption	Labor intensive Difficult to automate Emulsion formation
SFE	Environmentally friendly No required solvents Low operating temperatures (40–80 °C) Fast and high yield	Very expensive Complex equipment operating at high pressures High power consumption

Legend: DLLME: dispersive liquid–liquid microextraction; FPSE: fabric-phase sorptive extraction; MEPS: microextraction in packed sorbent; MSPD: matrix solid-phase dispersion; MSPE: magnetic solid-phase extraction; SFE: supercritical fluid extraction; SFOME: solidification of floating organic drop microextraction; SPME: solid-phase microextraction; µQuEChERS: micro-QuEChERS; µSPE: micro-solid-phase extraction.

## Data Availability

Not applicable.

## Supplementary Materials

The following supporting information can be downloaded at https://www.mdpi.com/article/10.3390/molecules27092953/s1: Table S1: Representative applications of GreETs for the analysis of biological samples; Table S2: Representative applications of GreETs for the analysis of food samples; Table S3: Representative applications of GreETs for the analysis of environmental samples.

## Abbreviations

AMPA: aminomethylphosphonic acid; APCI: atmospheric pressure chemical ionization; BIN: barrel insert needle; BID-SDME: bubble-in-drop; BFR: brominated flame retardants; CET: classical extraction techniques; CFME: continuous-flow microextraction; CIPS: cold-induced phase separation; CNFs: carbon nanofibers; CNTs: carbon nanotubes; DAD: diode-array detection; DCM: dichloromethane; DES: deep eutectic solvents; DLLME: dispersive liquid–liquid microextraction; dSPE: dispersive solid-phase extraction; DVB: divinylbenzene; ESI: electrospray ionization; ECD: electrochemical detection; EPT-SPME: effervescent pipette-tip solid-phase microextraction; EtAc: ethyl acetate; EtOH: ethanol; FA: formic acid; FAAS: flame atomic absorption spectrometry; FID: flame ionization detector; FGO-TD-PTFE: functional graphene oxide thermal desorption poly(tetrafluoroethylene); FLD: fluorescence detector; FPSE: fabric-phase sorbent extraction; GA: gallic acid; GAC: green analytical chemistry; GC–MS: gas chromatography–mass spectrometry; GreETs: green extraction techniques; HF-LPME: hollow fiber liquid-phase microextraction; HPLC: high performance liquid chromatography; HS: headspace; HRMS: high-resolution mass spectrometry; ICP: inductively coupled plasma; ILs: ionic liquids; IT: ion trap; LC–MS: liquid chromatography–mass spectrometry; LDH: layered double hydroxide; LLE: liquid–liquid extraction; LLME: liquid–liquid microextraction; LOD: limit of detection; LPE: liquid-phase extraction; M-ILs-SDME: magnetic ionic liquid single-drop microextraction; MAE: microwave-assisted extraction; MIP: molecular imprinted polymer; MIOMS-ir: molecularly imprinted ordered mesoporous silica imprint-removed silica; MISM: molecularly imprinted silica monolithic; MeOH: methanol; MEPS: microextraction by packed sorbent; MMF: multiple monolithic fiber; MNPs: magnetic nanoparticles; MOF: metal organic framework; MS: mass spectrometry; MSAμ: multisphere adsorptive microextraction; MSPD: matrix solid-phase dispersion; MSPE: magnetic solid-phase extraction; MS/MS: tandem mass spectrometry; MWCNTs: multiwalled CNTs; NADES: natural deep eutectic solvents; NPs: nanoparticles; NTD: needle trap device; NTME: needle trap microextraction; NSAIDs: nonsteroidal anti-inflammatory drugs; OES: optical emission spectrometry; PAHs: polycyclic aromatic hydrocarbons; PDA: photodiode array; PDMS: polydimethylsiloxane; PEFAE: pulsed electric-field-assisted extraction; PSE: pressurized solvent extraction; PT: pipette tip; PS/DVB-RP: reverse-phase polystyrene–divinylbenzene sorbent; QQQ: single quadrupole triple quadrupole; Q–TOF: quadrupole–time of flight; SBSE: stir-bar sorptive extraction; SCE: supercritical carbon dioxide extraction; SDME: single-drop microextraction; SFE: supercritical fluid extraction; SFOME: solidification of floating organic drop microextraction; SPE: solid-phase extraction; SPME: solid-phase microextraction; SWE: subcritical water extraction; TOF: quadrupole–time of flight; UA: ultrasound-assisted; UHPLC: ultrahigh performance liquid chromatography; UABE: ultrasound-assisted back extraction; UV: ultraviolet analysis; VOCs: volatile organic compounds; µQuEChERS: micro-QuEChERS; µSPE: micro-solid-phase extraction.
