# Supplementary material for: Green Extraction Techniques as Advanced Sample Preparation Approaches in Biological, Food, and Environmental Matrices: A Review
Source: Molecules. 2022 May 6;27(9):2953. doi: 10.3390/molecules27092953 (PMC9101692; doi:10.3390/molecules27092953)
Supplement: Supplementary file 1 [file molecules-27-02953-s001.zip › molecules-1704246-supplementary.pdf]

molecules

MDPI

Review

Green extraction techniques as advanced sample preparation approaches in biological, food, and environmental matrices: A review

José S. Câmara [1,2,*], Rosa Perestrelo [1], Cristina V. Berenguer [1], Carolina F. P. Andrade [1], Telma M. Gomes [1], Basit Olayanju [3], Abuzar Kabir [3,4], Cristina Vicente [5], and José A. C. Teixeira [5,6], Jorge A. M. Pereira [1]

CQM - Centro de Química da Madeira, NPRG, Universidade da Madeira, Campus Universitário da Penteada, 9020-105, Funchal, Portugal; rmp@staff.uma.pt (R.P.), cristina.berenguer@staff.uma.pt (C.V.B.), carolinafatimaandrade@hotmail.com (C.F.P.A.), telma_gomes_20@hotmail.com (T.M.G.)

[2] Departamento de Química, Faculdade de Ciências Exatas e Engenharia, Universidade da Madeira, Campus da Penteada, 9020-105 Funchal, Portugal; jsc@staff.uma.pt

[3] Department of Chemistry and Biochemistry, Florida International University, Miami, FL, USA

[4] Department of Pharmacy, Faculty of Allied Health Science, Daffodil International University, Dhaka-1207, Bangladesh abuzar.kabir@ulpgc.es

[5] CEB-Centre of Biological Engineering, Universidade do Minho, Campus de Gualtar, 4710-057 Braga, Portugal

[6] LABBELS–Associate Laboratory, Braga, Guimarães, Portugal;

* Correspondence: jorge.pereira@staff.uma.pt (J.A.M.P.); Tel.: +351 291705119

Supplementary Material

Table S1. Representative applications of GreETs for the analysis of biological samples.

	**Matrix**	**Analytical approach**	**Ref**
**Solid phase microextraction (SPME)**			
Aldehydes	Breath	GC-MS	[1]
Antidepressants	Urine	UHPLC-UV	[2]
Aromatic Amines	Urine	GC-MS/MS	[3]
BTEX	Urine	GC-FID	[4]
Doxorubicin	Lung tissue	LC-MS/MS	[5]
Estrogens	Urine	HPLC-FLD-UV	[6]
Flavonoids	Urine, feces	UHPLC-UV	[7]
Nicotine and cotinine	Hair	LC-MS/MS	[8]
Polyamines	Urine	GC-QqQ-MS	[9]
VOCs	Blood, urine	NMR	[10]
	Blood, urine	GC-MS	[11]
**Micro solid-phase extraction (μSPE)**			
Carbamazepine	Urine	HPLC-UV	[12]
Carotenoids and vitamins	Serum	HPLC-DAD	[13]
Glyphosate, aminomethyl phosphonic	Urine	LC-HRMS	[14]
Ni, Pb	Blood	AT-AAS	[15]
Ofloxacin and sparfloxacin	Plasma	HPLC-UV	[16]
Oxazepam, nitrazepam	Plasma, urine	HPLC-DAD	[17]
PAHs	Urine	LC-MS	[18]
Pb	Hair	GAAFS	[19]
	Blood and urine	FAAS	[20][a]
Phenolphthalein	Urine	HPLC-PDA	[21]
**Microextraction in packed syringe (MEPS)**			
β-blocker	Urine	LC-FLD	[22]
Amphetamine-type stimulants	Urine	GC-MS	[23]
Anesthetics	Plasma, saliva	HPLC-MS	[24]
Antidepressants	Urine	UHPLC-PDA	[25]
Antisense oligonucleotide	Plasma	LC-UV	[26]
Anti-inflammatory drugs and fluoroquinolones	Plasma, urine	UHPLC-PDA	[27]
Asthma biomarkers	Urine	UHPLC-PDA	[28]
Azole antimicrobial drugs	Plasma, urine	HPLC-DAD	[29]
Lamotrigine (*garcinia cambogia*)	Rat plasma	LC-DAD	[30]
Levofoxacin	Plasma	UHPL-UV	[31]
Mandelic Acid	Urine	LC-UV	[32]
Nifrofurantoin	Urine	UV-VIS	[33]
Opiates	Blood	GC-MS/MS	[34]
**Magnetic Solid phase extraction (MSPE)**			
Antiepileptic drugs	Plasma	HPLC-DAD	[35]
Atorvastatin, simvastatin	Urine	HPLC-PDA	[36]
Ibuprofen	Plasma	HPLC-DAD	[37]
Fluoxetine	Urine	Spectrophotometry	[38]
Methadone	Urine, plasma	GC–FID, GC–MS	[39]
NSAIDs	Urine	HPLC-DAD	[40]

Parabens, bisphenol A	Breast milk, urine	HPLC-UV	[41]
Pseudoephedrine	Urine	HPLC-UV	[42]
**Stir Bar Sorptive Extraction (SBSE)**			
Amphetamine and methamphetamine	Urine	HPLC-UV	[43]
Antidepressants	Urine	HPLC	[44][a]
Ibuprofen, aspirin, and venlafaxine	Urine	GC-MS	[45]
Losartan and Valsartan	Plasma	LC-MS	[46]
Propranolol	Urine	HPLC-UV	[47]
4-Chloro-1-Naphthol	Urine, wastewater	HPLC-UV	[48]
**Pipette Tip Solid-Phase Extraction (PT-SPE)**			
Indometacin and acemetacin	Urine	HPLC-UV	[49]
Ketoconazole	Urine	HPLC-DAD	[50]
PAHs (16)	Blood	GC-MS	[51]
Toluene and xylene exposure biomarkers	Urine	HPLC-UV	[52][a]
**Fabric phase sorptive extraction (FPSE)**			
Androgens and progestogens	Urine, wastewater	UHPLC-MS/MS	[53]
Anticancer drugs	Blood, plasma and urine	HPLC-DAD	[54]
Antidepressants	Urine	HPLC-DAD	[55]
Azole antimicrobial drugs	Plasma, urine	HPLC-DAD	[56]
Benzodiazepines	Blood	HPLC-PDA	[57]
Bisphenol A, residual dental restorative material	Breast milk	HPLC-DAD	[58]
Cu(II), Ni(II), Zn(II), Pb(II), and Cd(II)	Urine	FAAS	[59]
Inflammatory bowel disease treatment drugs	Blood, plasma and urine	HPLC-DAD	[60]
NSAIDs	Saliva	HPLC-DAD	[61]
Parabens	Blood, plasma and urine	HPLC-DAD	[62]
Radiation exposure markers	Blood	LC-MS	[63]
**Dispersive liquid-liquid microextraction (DLLME)**			
Antidepressants	Plasma, blood	LC-MS/MS	[64]
Androgen receptor modulators	Urine	UHPLC-MS/MS	[65]
Benzodiazepines	Blood	LC-MS/MS	[66]
Carbamazepine and lamotrigine	Serum, plasma and urine	HPLC-DAD	[67][a]
Empagliflozin, dapagliflozin and canagliflozin	Plasma	HPLC-DAD	[68]
Methotrexate	Urine	Spectrophotometer	[69][a]
Neurotransmitters	Urine	HILIC-MS	[70]
Ni and Co	Blood, serum and urine	ET-AAS	[71][a]
Pb	Blood and scalp hair	FAAS	[72]
Soluble vitamins and carotenoids	Serum	HPLC-PDA	[73][a]
Suvorexant (sedative)	Urine	UHPLC–MS/MS	[74]
Trans,trans-muconic acid	Urine	HPLC-DAD	[75][a]
**µQuEChERS**			
Bisphenol A	Urine	GC-MS	[76]
Fluoxetine and clomipramine	Urine	UHPLC-PDA	[77]
Psychotropic Drugs	Serum and Postmortem	UHPLC–MS-MS	[78]
Wild life pollutants	Blood	LC-MS/MS, GC-MS/MS	[79][a]

Legend: [a] – application involving the use of ILs or DES; DES: deep eutectic solvents, ET-AAS: electro thermal atomic absorption spectrometry; FAAS: flame atomic absorption spectrometry; GC-FID: gas chromatography with flame ionization detector; GC-MS/MS: gas chromatography tandem mass spectrometer; GC-MS: gas chromatography coupled with mass spectrometry; GFAAS: graphite furnace atomic absorption

spectrometry; HPLC: high performance liquid chromatography; HPLC-FLD: high performance liquid chromatography with fluorescence detection; HPLC-UV: high-performance liquid chromatography combined with an ultraviolet detector; ILs: ionic liquids; LC-HRMS: liquid chromatography-high resolution mass spectrometry; LC-MS/MS: liquid chromatography tandem mass spectrometry; NMR: nuclear magnetic resonance; PAHs: polycyclic aromatic hydrocarbons; UHPLC: ultra-high performance liquid chromatography; UV/Vis: ultraviolet-visible spectrophotometry.

Table S2. Representative applications of GreETs for the analysis of food samples.

**Analytes**	**Matrix**	**Analytical approach**	**Ref**
**Solid phase microextraction (SPME)**			
Acrylamide	Biscuits	GC-MS	[80]
Organophosphorus pesticides	Wine, juice	GC-FPD	[81]
Organophosphorus pesticides	Wine and juice	GC-ECD	[81][a]
Pesticides	Tomato	GC-FID	[82][a]
Phthalates	Food packing	GC-MS	[83]
	Wines	GC-MS	[84]
Synthetic phenolic antioxidants	Food-grade lubricants	GC-MS	[85]
VOCs	Walnut oils, *Hongeo,* dairy products, melon	GC-MS	[86–89]
Xanthines	Coffee beverages	UPLC-MS/MS	[90]
1,4-dioxane, 1,2,3-trichloropropane	Corn, wheat and tomato	GC-MS	[91]
2-dodecylcyclobutanone, 2-tetradecylcyclobutanone	Dairy products	GC-MS	[92]
3,4-Dihydroxybenzoic acid	*Ilex chinensis* Sims	SPME	[93][a]
**Micro solid-phase extraction (μSPE)**			
Aflatoxins	Non-dairy beverages	LC-MS/MS	[94]
Bisphenol A	Bottled water	HPLC-UV	[95]
Chlorobenzenes	Apple juice	HPLC-DAD	[96]
Diazinon	Tomato, cucumber, lettuce	HPLC-UV	[97]
Fluoroquinoles and amantadine	Chicken	ESI-QQQ-MS	[98]
Pesticides	Catfish	GC-MS/MS	[99]
PAHs	Roast potatoes, baked fish	HPLC-DAD	[100]
	Spent tea leaves	GC-FID	[101]
	Sunflower oil	GC-MS	[102]
	Vegetables and fruit juice	GC-FID	[103][a]
Pesticides	Cereals	GC-MS	[104]
Phenolic compounds	Fruit juice samples	HPLC-UV	[105][a]
Rosmarinic acid	Medicinal plants	HPLC-UV	[106]
Se	Green tea	FS	[107]
Trace metals (As, Cd, Cr, Co, Sb, Pb and Tl)	Vegetables	ICP-OES	[108]

Vitamin D3	Bovine milk	HPLC-UV	[109]
**Microextraction in packed syringe (MEPS)**			
Caffeine	Soft and energy drinks	HPLC-UV	[110]
Ciprofloxacin, enrofloxacin, marbofloxacin	Bovine milk	UHPLC-PDA	[111]
Clorophenhols, phenoxy acid herbicides, PAHs	Rice	GC-FID	[112]
Fipronil, fluazuron	Drinking water	UHPLC-DAD	[113]
Pesticides	Coffee	GC-MS/MS	[114][a]
Parabens	Vegetable oil	HPLC-MS	[115]
Pesticides	Apple juice	HPLC-UV	[116]
	Coffe	GC-MS/MS	[114]
PAHs	Apple	GC-MS	[117]
Polyphenols	Baby food	UHPLC-PDA	[118]
Steroids	Bovine milk	HPLC-DAD	[119]
Polybrominated diphenyl ethers	Egg	GC-MS	[120]
**Matrix solid-phase dispersion (MSPD)**			
EDCs, organochlorine pesticides	Mussels	HPLC-DAD	[121]
Ergosterol	Edible fungi	LC-DAD	[122]
Flavonoids	Buckwheat sprouts	HPLC-PDA	[123]
	Dendrobium huoshanense	UHPLC-Q-TOF/MS	[124]
Mangiferin, hyperoside	Mango processing waste	LC-UV	[125]
Pesticides	Peppers	GC-MS	[126]
	Chicken eggs	GC-MS	[127]
	Vegetables	HPLC-DAD	[128][a]
Polyphenols	Apple	LC-DAD-MS	[129]
	Grape residues	HPLC-DAD	[130]
Pharmacologically active substances, pesticide residues	Microalgae (Chrorella and spirulina)	GC-MS	[131]
Rhodamine B	Chili, tomato ketchup, jelly	HPLC-UV	[132]
Sulfonylurea herbicides	Soybean, peanut, corn	LC-MS/MS	[133]
Triazine herbicides	Brown sugar	HPLC-PDA	[134][a]
**Magnetic solid phase extraction (MSPE)**			
Acrylamide	Biscuits,	HPLC-UV	[135]
	Fruits, vegetables, chips	GC-MS	[136]
Bisphenols	Fruit juices	UHPLC-MS/MS	[137]
Caffeine	Teas, coffees, cocoa, chocolates	GC-MS	[138]
Co(II) and Hg(II)	Vegetables, meat, fish, milk	ICP-OES	[139]
Cu(II)	Cereals	FAAS	[140]
PAHs	Beef and pork	HPLC-FLD	[141][a]

PAHs	Tea	GC-MS,	[142]
	Rice	GC-FID	[143]
Parabens	Cola and green tea	GC-MS	[144][a]
Pesticide residues	Fruits and vegetables	HPLC-UV	[145][a]
Plant growth regulators	Vegetables	GC-MS	[146]
Sb(V)	Soft drinks, orange juice, beers	ET-AAS	[147]
**Fabric phase sorptive extraction (FPSE)**			
Estrogenic EDCs, bisphenol A residues	Milk	HPLC-UV, LC-MS/MS	[148]
Sulfonamides	Milk	HPLC-UV	[149]
Fungicides	Tea	HPLC-DAD	[150]
Fungicides	Tea infusions	HPLC-DAD	[150][a]
Fungicides, insecticides	Wine	UPLC-MS/MS	[151]
Oligomers	Pineapple juice	UHPLC-MS	[152]
Organophosphorus pesticides	Beans, tomato, brinjal, cabbage	GC-MS	[153]
PAHs	Nutritional supplements	HPLC-FLR	[154]
Steroid hormone residues	Raw milk	UHPLC-MS/MS	[155]
Tetracicline residues	Milk	HPLC-UV	[156]
Triazine herbicides	Fruit juices	HPLC-DAD	[157]
**Dispersive liquid-liquid microextraction (DLLME)**			
Bendiocarb, azinphos-ethyl	Orange juice, tomato, potato	IMS	[158]
Benzoylurea pesticides	Tea and fruit juices	DES/HPLC-UV	[159]
Cd, As	Wine	FAAS	[160]
Cd, Cu, Fe	Margarine	HR-CS ET ASS	[161]
Chloramphenicol	Honey	UHPLC-MS/MS	[162]
Cr	Water, beverages, vegetables	FAAS	[163]
Cu, Cd, Pb	Honey	FAAS	[164]
Histamine	Fish and meat	UV-Vis	[165][a]
Mycotoxins	Rice bran	LC-MS/MS	[166]
NSAIDs	Milk	HPLC-UV	[167]
Organothiophosphate pesticides	Honey samples	GC-MS	[168][a]
Se(VI)	Cereals, vegetables	HG-AAS	[169]
Strobilurin fungicides	Apples	HPLC-UV	[170]
**µQuEChERS**			
Insecticides	Patotoes	UHPLC-PDA	[171]
PAHs	Coffee, tea	GC-MS	[172]
Pesticide residues	Wine	UHPLC-MS/MS	[173]
Polyphenols	Baby food	UHPLC-PDA	[174]
	Fruits and Vegetables	UHPLC-PDA	[175]

	Endemic blueberries	LC-ESI-MS/MS	[176]
Pyrrolizidine alkaloids	Aromatic herbs	UHPLC-MS/MS	[177,178]
**Single drop microextraction (SDME)**			
2-phenoxyethanol	Fish	GC-MS	[179]
Acrylamide	Bread, potato chips, cookies	GC-ECD	[180]
Ammonia	Milk, yoghurt, cheese, beer	CCD-array	[181]
Cu(II)	Tap water	ET-AAS	[182]
Ethyl carbamate	Wine	GC-MS	[183]
Formaldehyde	Octopus, chicken	UV-Vis	[184]
Tartrazine	Food	IV	[185]
**Solidification of floating organic drop microextraction (SFOME)**			
β-Lactam antibiotic residues	Egg, honey, chicken muscle,	HPLC-PDA	[186]
Antibiotics	Honey		[187]
Acidic pesticides	Tomato	GC-MS	[188]
Cd	Biscuit	GFAAS	[189]
Free fatty acid	Milk	GC-MS	[190] [a]
Mn(II)	Energy drink, ice tea, sprite drink	GFAAS	[191]
Ni(II), Co(II)	Broccoli, spinach	FAAS	[192]
Organochlorine pesticides	Cocoa powder	GC-ECD	[193]
Organophosphorus and pyrethroid pesticides	Organic and conventional vegetables	GC-MS	[194]
PAHs	Honey	GC-MS	[195]
Pesticides	Milk	GC–FID	[63]
Pesticides	Fruit juices and vegetables	GC-MS	[196] [a]
Phytosterols	Edible oil	GC-MS	[197]
Terpenes	Spices	GC-MS	[198] [a]
Volatile components	*Satureja hortensis* L. leaves	GC-MS	[199] [a]
**Pulsed electric field-assisted extraction (PEAE)**			
Bioactive compounds	Cocoa bean shell, coffee silverskin	HPLC-PDA	[200]
	thinned peach fruits	HPLC-PDA	[201]
Carotenoids	Tomato wastes	HPLC-UV	[202]
Functional compounds	Nepeta binaludensis	HPLC-UV	[203]
Phenolic compounds	almond red leaves	HPLC-UV	[204]
	Rosemary, thyme by-products	UPLC-MS/MS	[205]
Procyanidins	*Vitis amurensis* seeds	UV-Vis	[206]
Sulforaphane	Broccoli florets	HPLC-DAD	[207]
**Supercritical fluid extraction (SFE)**			
Antioxidant and antibacterial compounds	Feijoa leaf	LC-MS/MS	[208]
Fatty acids	Borage seed oil	UV-Vis, GC	[209]

Oils	*Terminalia catappa* fruits	GC, IV	[210]
Oleoresins	*Capsicum annuum, Capsicum chinense,*	HPLC-UV	[211]
	Sea buckthorn pomace	HPLC-UV, GC/MS	[212]
Phytochemicals	*Terminalia chebula* pulp	UV-Vis	[213]
Polar lipid fraction	Blackberry, passion fruits	LC-DAD-MS/MS	[214]
Seed oil and active compounds	Peel from pumpkin	UV-Vis	[215]
**Subcritical water extraction (SWE)**			
Antioxidant protein hydrolysates	Shellfish waste	UV-Vis	[216]
Anthocyanins	Raspberry	HPLC-DAD	[217]
Bioactive compounds	Pumpkin peel, apple bark	HPLC-UV/Vis, GC-MS	[218,219]
Fatty acids	*Ulva lactuca, Caulerpa racemosa*	GC	[220]
Hesperidin and narirutin	*Citrus unshiu* peel	HPLC-UV/Vis	[221]
Pectic polysaccharides	Apple pomace	UV-Vis, FTIR	[222]
Phenolic antioxidants	Chestnut shells	HPLC-MS	[223]
Phenolic compounds	Avocado fruit flesh	UV-Vis	[224]
Phytochemical compounds	*Moringa oleifera, Sauropus androgynus, Sesbania grandiflora*	UV-Vis, FTIR	[225]
Scopoletin, alizarin, and rutin	*Morinda citrifolia*	HPLC-UV/Vis	[226]

Legend: [a] – application involving the use of ILs or DES; CCD-array: charge-coupled device; DES: deep eutectic solvents; EDCs: endocrine-disrupting compounds; FAAS: flame atomic absorption spectrometry; FTIR: Fourier-transform infrared spectroscopy; FS: fluorescent spectroscopy; GC: gas chromatography; GC-ECD: gas chromatography with electron capture detector; GC-FID: gas chromatography with flame ionization detector; GC-MS/MS: gas chromatography tandem mass spectrometer; GC-MS: gas chromatography coupled with mass spectrometry; GFAAS: graphite furnace atomic absorption spectrometry; GFAAS: graphite furnace atomic absorption spectrometry; HPLC: high performance liquid chromatography; HPLC-FLD: high performance liquid chromatography with fluorescence detection; HPLC-FLD: high performance liquid chromatography with fluorescence detection; HPLC-UV: high-performance liquid chromatography combined with an ultraviolet detector; ICP-OES: inductively coupled plasma optical emission spectrometry; ILs: ionic liquids; LC-MS/MS: liquid chromatography tandem mass spectrometry; PAHs: polycyclic aromatic hydrocarbons; UHPLC: ultra-high performance liquid chromatography; UHPLC-MS/MS: ultra-high performance liquid chromatography tandem mass spectrometry; UV/Vis: ultraviolet-visible spectrophotometry; VOCs: volatile organic compounds.

molecules MDPI

Table S3. Representative applications of GreETs for the analysis of environmental samples.

Analytes	Matrix	Analytical approach	Ref
**Solid phase microextraction (SPME)**			
Endocrine-disruptive pesticides	Water	GC-MS	[227]
Estrogens	Water	HPLC	[228][a]
Microplastic	Coral reef invertebrates	LC-MS	[229]
NSAIDs	Lake water	HPLC-UV	[230][a]
Organophosphorus pesticides	Water	CD-IMS	[231]
PAHs	Soil, rain, water	GC-FID, GC-MS	[232–235]
PAHs	Water	GC-FID	[236][a]
Pb (II)	Tap water	FI-ICP-OES	[237][a]
Phthalate esters	Water	GC-FID	[238][a]
Pesticides	Water	GC-MS	[239]
Toluene, ethylbenzene and o-xylene	Water	GC-FID	[240][a]
VOCs	Wastewater, air	GC-MS	[241,242]
Ultraviolet filters	Water	GC-MS	[243][a]
**Micro solid-phase extraction (µSPE)**			
Aluminium	Wastewater	ICP-MS	[244]
Chemical warfare agents	Tap water	GC-FID-MS/MS	[245]
Chlorobenzenes	Water, soil	HPLC-DAD	[96]
Diazinon	Soil	HPLC-UV	[97]
Dyes	Water	Spectrophotometry	[246][a]
EDCs	Wastewater, water, ambient air	HPLC-DAD,GC-MS/MS	[247,248]
Herbicides	Water	UHPLC-MS/MS	[249]
Non-steroidal anti-inflammatory drugs	Water	HPLC-UV	[250]
Organochlorine pesticides	Water	GC-MS	[251]
PAHs	Seawater, water	HPLC-FLR, GC-FID	[101,252]
Polar herbicides	Tap and reservoir waters	UHPLC-MS/MS	[253]
Trace metals (As, Cd, Cr, Co, Sb, Pb and Tl)	Water	ICP-OES	[108]
**Microextraction in packed syringe (MEPS)**			
Benzene, phenol and their derivates	Water	HPLC-UV	[254]
Diazinon	Water	CD-IMS	[255]
Fipronil, fluazuron residues	Wastewater	UHPLC-DAD	[113]
Fluoxetine	Wastewater, river, dam water	FL	[256]
La^{3+}, Tb^{3+}	Water	ICP-OES	[257]
PAHs	Soil, water, snow	HPLC-UV/Vis,<br>GC-FID, GC-MS	[258–260]
Organophosphorus pesticides	Water	GC-MS	[261]

Phthalates	Tap and river water	GC-FID	[262]
**Pipette Tip Solid-Phase Extraction (PT-SPE)**			
Methyl and propyl parabens	Wastewater and shampoo samples	Spectrophotometry	[263]
Sulfamerazine	River water	HPLC	[264][a]
2,4-dichlorophenoxyacetic	Lake water	HPLC-SPD	[265]
**Matrix solid-phase dispersion (MSPD)**			
Atrazine	Water	HPLC-UV/Vis-DAD	[266]
Azole fungicides	Fish	HPLC-DAD	[267]
Emerging contaminants	Aquatic plants	GC-MS	[268]
EDCs	Mussels	HPLC-DAD	[121]
Fluoroquinolones	Water	LC-MS/MS	[269]
Organophosphorus flame retardants	Marine mussel	LC-MS/MS	[270]
PAHs	Soil	GC-MS	[271]
Pesticides	Honeybees, water	GC-MS	[272,273]
Triazine herbicides	Marine sediments	HPLC-DAD	[274]
Bisphenol Contaminants	Bee polen	HPLC-DAD	[275]
Fluazuron (acaricid)	Bovine plasma	LC-UV	[276]
**Magnetic solid phase extraction (MSPE)**			
Benzoylurea pesticides	Water	HPLC-DAD	[277]
Cu (II), Cd (II), Cr (III), Zn (II), Pb (II)	Water	ICP-OES	[278]
Heterocyclic pesticides	Water	HPLC-DAD-FLR	[279]
Microcystins	Lake water	UHPLC-MS/MS	[280][a]
Organophosphorus pesticides	Water	HPLC-UV	[281]
PAHs	River, lake and sludge waters and soil	GC-MS	[282][a]
Pharmaceutical compounds	River and dam water	HPLC-UV	[283][a]
PAHs	Water	GC-MS	[284]
Phenols	Water	HPLC-UV	[285]
Se, Te	Water	ICP-MS	[286]
Strobilurin fungicides	Water	HPLC-MS/MS	[287]
Sulfonamide antibiotics	Water	HPLC-MS/MS	[288]
Triazole fungicides	Water	HPLC-DAD	[289]
**Fabric phase sorptive extraction (FPSE)**			
Amphetamine drugs	Water	LC-MS	[290]
Antidepressant drugs	Wastewater, lake water	HPLC-DAD	[291]
Cytostatic drug residues	Water	UHPLC-MS/MS	[292]
Emerging contaminants	Wastewater	UHPLC–LTQ-Orbitrap MS	[293]
Fungicides	Water	GC-MS	[294]

Parabens	Wastewater	HPLC-PDA	[295]
PAHs	Water	CD-IMS	[296]
Pesticide residues	Water	HPLC-PDA	[297]
Substituted phenols	Water	HPLC-UV	[298]
UV filters	Water	GC-MS/MS	[299]
Lead and cadmium	Wastewater	FAAS	[300]
Brominated flame retardants	Wastewater	HPLC	[301]
Plastic additives contaminants	Food packages	UPLC-MS	[302]
Sexual pheromones	Environmental air	GC-MS	[303]
**Dispersive liquid-liquid microextraction (DLLME)**			
Antibiotics	Tap, waste, and seafood market water	HPLC-UV	[304] [a]
Aromatic amines	Water	HPLC-UV	[305]
Cd	Water	TS-FF-AAS	[306]
Cr	Water	GFAAS	[307]
Cu	Wastewater	FAAS	[308] [a]
Dyes	Water	RSM-CCD	[309]
Fluoroquinolones	River water	HPLC-FLD	[310]
Herbicides	Water	GC-MS	[311]
Lipophilic organic UV filters	River, sea, and swimming pool water	TD-GC-MS	[312] [a]
Ni^{2+}, Co^{2+}, Cd^{2+}, Cu^{2+}, Pb^{2+}	River, lake water	LC-UV	[313]
Ni (II) and Co (II)	Water	DES/GFAAS	[314]
NSAIDs	Water	HPLC-UV	[167]
PAHs	Water	GC-MS	[315] [a]
Pesticides	Water	HPLC-UV	[316] [a]
Polybrominated biphenyls	Water	HPLC-UV	[317]
Pyrethroid insecticides	Water	HPLC-UV	[318]
Tetracycline	Water	HPLC-UV	[319]
Steroids	Water	HPLC-PDA	[320] [a]
Phenol	wastewater	Android app Color Grab	[321]
**μQuEChERs**			
Acidic, basic, neutral, amphiphilic species	Soil	LC-MS	[322]
Insecticides	Guttation fluids	LC-MS/MS	[323]
Pesticides	Arthropods, gastropods	GC-MS/MS	[324]
Pesticides, insecticides	Pollen, nectar	LC-MS/MS	[325]
VOCs	Small organisms (zebrafish)	GC-MS/MS	[326]
**Single drop microextraction (SDME)**			
Aromatic compounds	Lake water	HPLC-UV	[327] [a]
Cu (II)	Tap water, seawater	GFAAS	[182]

Endocrine disrupting compounds	Water	HPLC-DAD	[328][a]
Mn (II)	Fish	GFAAS	[329]
PAHs	Tap water	GC-MS	[330]
PAHs	River water and wastewater	GC-MS	[330][a]
Pesticides	Mango	GC-MS	[331]
Ranitidine	Wastewater	LC-MS/MS	[332]
V (V)	Water	DIC	[333]
Volatile aromatic hydrocarbons	Water	GC-FID	[334][a]
**Solidification of floating organic drop microextraction (SFOME)**			
Antiviral agents	River water	HPLC-UV	[335]
Cd	River, sea, and tap water	FAAS	[336]
Pb	Water	GFAAS	[336]
Ni, Co, Cu	Fish	FAAS	[337]
NSAIDs	Water	HPLC-UV/Vis	[338]
Phenol, chlorophenols	Water	HPLC	[339]
Benzophenone and salicylate ultraviolet filters	Water	HPLC-Vis	[340]
**Supercritical fluid extraction (SFE)**			
Ag	Electronic waste	ICP	[341]
Neonicotinoid pesticides	Green onion	LC-MS	[342]
PAHs	Soil	SFC-MS	[343]
Petroleum biomarkers	Tar balls, crude oils	GC-MS	[344]
Petroleum hydrocarbons	Soil	GC-FID	[345]
Polyethylene and polypropylene waxes	Polyolefin plastic feedstock	GC-MS	[346]
Solanesol	Tobacco residues	HPLC-DAD	[347]
**Subcritical water extraction (SWE)**			
Co, Li and Mn	Spent lithium-ion batteries	XPS	[348]
Crude oil	Soil	BBD-RSM	[349]
Oil shale	Mines	GC-MS	[350]
PAHs	Atmospheric particulate matter	GC-MS/MS	[351]
Petroleum hydrocarbons, oil	Soil	GC-MS	[352]
VOCs	Sewage sludge	GC-MS	[353]

Legend: [a] – application involving the use of ILs or DES; BBD-RSM: Box-Behnken Design under response surface methodology; DES: deep eutectic solvents; DIC: digital image colorimetry; EDCs: endocrine-disrupting compounds; FAAS: flame atomic absorption spectrometry; GC-FID: gas chromatography with flame ionization detector; GC-MS/MS: gas chromatography tandem mass spectrometer; GC-MS: gas chromatography coupled with mass spectrometry; GFAAS: graphite furnace atomic absorption spectrometry; GFAAS: graphite furnace atomic absorption spectrometry; HPLC: high performance liquid chromatography; HPLC-FLD: high performance liquid chromatography with fluorescence detection; HPLC-FLD: high performance liquid chromatography with fluorescence detection; HPLC-UV: high-performance liquid chromatography combined with an ultraviolet detector; ICP-MS: Inductively coupled plasma mass spectrometry; ILs: ionic liquids; LC-MS/MS: liquid chromatography tandem mass spectrometry; PAHs: polycyclic aromatic hydrocarbons; RSM-CCD: response surface methodology with central composite design; SFC-MS: supercritical fluid chromatography mass spectrometry; TS-FF-AAS: thermospray flame furnace atomic absorption spectrometry; UHPLC: ultra-high performance liquid chromatography; UHPLC-MS/MS: ultra-high performance liquid chromatography tandem mass spectrometry; VOCs: volatile organic compounds.

References

1. Yu, L.Q.; Wang, L.Y.; Su, F.H.; Hao, P.Y.; Wang, H.; Lv, Y.K. A gate-opening controlled metal-organic framework for selective solid-phase microextraction of aldehydes from exhaled breath of lung cancer patients. *Microchimica Acta* **2018**, *185*, 1-7, doi:10.1007/s00604-018-2843-1.
2. Fresco-Cala, B.; Mompó-Roselló, Ó.; Simó-Alfonso, E.F.; Cárdenas, S.; Herrero-Martínez, J.M. Carbon nanotube-modified monolithic polymethacrylate pipette tips for (micro)solid-phase extraction of antidepressants from urine samples. *Microchimica Acta* **2018**, *185*, 1-7, doi:10.1007/s00604-017-2659-4.
3. Niu, J.; Zhao, X.; Jin, Y.; Yang, G.; Li, Z.; Wang, J.; Zhao, R.; Li, Z. Determination of aromatic amines in the urine of smokers using a porous organic framework (JUC-Z2)-coated solid-phase microextraction fiber. *J. Chromatogr. A* **2018**, *1555*, 37-44, doi:10.1016/j.chroma.2018.04.059.
4. Tajik, L.; Bahrami, A.; Ghiasvand, A.; Shahna, F.G. Determination of BTEX in urine samples using cooling/heating-assisted headspace solid-phase microextraction. *Chemical Papers* **2017**, *71*, 1829-1838, doi:10.1007/s11696-017-0176-x.
5. Roszkowska, A.; Miękus, N.; Bączek, T. Application of solid-phase microextraction in current biomedical research. *J. Sep. Sci.* **2019**, *42*, 285-302, doi:10.1002/jssc.201800785.
6. Luo, X.; Li, G.; Hu, Y. In-tube solid-phase microextraction based on NH2-MIL-53(Al)-polymer monolithic column for online coupling with high-performance liquid chromatography for directly sensitive analysis of estrogens in human urine. *Talanta* **2017**, *165*, 377-383, doi:10.1016/j.talanta.2016.12.050.
7. Wang, N.; Xin, H.; Zhang, Q.; Jiang, Y.; Wang, X.; Shou, D.; Qin, L. Carbon nanotube-polymer composite for effervescent pipette tip solid phase microextraction of alkaloids and flavonoids from Epimedii herba in biological samples. *Talanta* **2017**, *162*, 10-18, doi:10.1016/j.talanta.2016.09.059.
8. Inukai, T.; Kaji, S.; Kataoka, H. Analysis of nicotine and cotinine in hair by on-line in-tube solid-phase microextraction coupled with liquid chromatography-tandem mass spectrometry as biomarkers of exposure to tobacco smoke. *J. Pharm. Biomed. Anal.* **2018**, *156*, 272-277, doi:10.1016/j.jpba.2018.04.032.
9. Naccarato, A.; Elliani, R.; Cavaliere, B.; Sindona, G.; Tagarelli, A. Development of a fast and simple gas chromatographic protocol based on the combined use of alkyl chloroformate and solid phase microextraction for the assay of polyamines in human urine. *J. Chromatogr. A* **2018**, *1549*, 1-13, doi:10.1016/j.chroma.2018.03.034.
10. Silva, C.L.; Perestrelo, R.; Capelinha, F.; Tomás, H.; Câmara, J.S. An integrative approach based on GC–qMS and NMR metabolomics data as a comprehensive strategy to search potential breast cancer biomarkers. *Metabolomics* **2021**, *17*, doi:10.1007/S11306-021-01823-1.
11. Bannaga, A.S.; Tyagi, H.; Daulton, E.; Covington, J.A.; Arasaradnam, R.P. Exploratory study using urinary volatile organic compounds for the detection of hepatocellular carcinoma. *Molecules 2021, Vol. 26, Page 2447* **2021**, *26*, 2447-2447, doi:10.3390/MOLECULES26092447.
12. Rezaei Kahkha, M.R.; Oveisi, A.R.; Kaykhaii, M.; Rezaei Kahkha, B. Determination of carbamazepine in urine and water samples using amino-functionalized metal–organic framework as sorbent. *Chem. Cent. J.* **2018**, *12*, 1-12, doi:10.1186/s13065-018-0446-x.
13. Qi, F.F.; Ma, T.Y.; Fan, Y.M.; Chu, L.l.; Liu, Y.; Yu, Y. Nanoparticle-based polyacrylonitrile monolithic column for highly efficient micro solid-phase extraction of carotenoids and vitamins in human serum. *J. Chromatogr. A* **2021**, *1635*, 461755-461755, doi:10.1016/J.CHROMA.2020.461755.
14. Chen, D.; Miao, H.; Zhao, Y.; Wu, Y. A simple liquid chromatography-high resolution mass spectrometry method for the determination of glyphosate and aminomethylphosphonic acid in human urine using cold-induced phase separation and hydrophilic pipette tip solid-phase extraction. *J. Chromatogr. A* **2019**, *1587*, 73-78, doi:10.1016/j.chroma.2018.11.030.
15. Lari, A.; Esmaeili, N.; Ghafari, H. Ionic liquid functionlized on multiwall carbon nanotubes for nickel and lead determination in human serum and urine samples by micro solid-phase extraction. *Analytical Methods in Environmental Chemistry Journal* **2021**, *4*, 72-85, doi:10.24200/AMECJ.V4.I02.144.
16. Owaid, S.J.; Yahaya, N.; Rahim, N.Y.; Mohammad, R.A.E.; Jajuli, M.N.; Miskam, M. Development of dispersive micro-solid phase extraction for the analysis of ofloxacin and sparfloxacin in human plasma (Pembangunan Pengekstrakan Fasa Pepejal-Mikro Disesarkan untuk Analisis Oflosaksin dan Sparflosaksin dalam Plasma Manusia). *Malaysian Journal of Analytical Sciences* **2020**, *24*, 893-905.
17. Amini, S.; Ebrahimzadeh, H.; Seidi, S.; Jalilian, N. Polyacrylonitrile/MIL-53(Fe) electrospun nanofiber for pipette-tip micro solid phase extraction of nitrazepam and oxazepam followed by HPLC analysis. *Microchimica Acta* **2020**, *187*, doi:10.1007/s00604-020-4112-3.
18. Chen, D.; Xu, H. Electrospun core-shell nanofibers as an adsorbent for on-line micro-solid phase extraction of monohydroxy derivatives of polycyclic aromatic hydrocarbons from human urine, and their quantitation by LC-MS. *Microchimica Acta* **2020**, *187*, 1-10, doi:10.1007/S00604-019-4007-3/TABLES/3.
19. Mirzaee, M.T.; Seidi, S.; Alizadeh, R. Pipette-tip SPE based on Graphene/ZnCr LDH for Pb(II) analysis in hair samples followed by GFAAS. *Anal. Biochem.* **2021**, *612*, 113949-113949, doi:10.1016/j.ab.2020.113949.
20. Shirkhanloo, H.; Davari Ahranjani, S. A lead analysis based on amine functionalized bimodal mesoporous silica nanoparticles in human biological samples by ultrasound assisted-ionic liquid trap-micro solid phase extraction. *J. Pharm. Biomed. Anal.* **2018**, *157*, 1-9, doi:10.1016/j.jpba.2018.05.004.

21. Jalilian, N.; Ebrahimzadeh, H.; Asgharinezhad, A.A.; Khodayari, P. Magnetic molecularly imprinted polymer for the selective dispersive micro solid phase extraction of phenolphthalein in urine samples and herbal slimming capsules prior to HPLC-PDA analysis. *Microchem. J.* **2021**, *160*, doi:10.1016/J.MICROC.2020.105712.
22. Šatínský, D.; Sobek, V.; Lhotská, I.; Solich, P. Micro-extraction by packed sorbent coupled on-line to a column-switching chromatography system – A case study on the determination of three beta-blockers in human urine. *Microchem. J.* **2019**, *147*, 60-66, doi:10.1016/j.microc.2019.02.069.
23. Malaca, S.; Rosado, T.; Restolho, J.; Rodilla, J.M.; Rocha, P.M.M.; Silva, L.; Margalho, C.; Barroso, M.; Gallardo, E. Determination of amphetamine-type stimulants in urine samples using microextraction by packed sorbent and gas chromatography-mass spectrometry. *Journal of Chromatography B: Analytical Technologies in the Biomedical and Life Sciences* **2019**, *1120*, 41-50, doi:10.1016/j.jchromb.2019.04.052.
24. Ahmadi, M.; Moein, M.M.; Madrakian, T.; Afkhami, A.; Bahar, S.; Abdel-Rehim, M. Reduced graphene oxide as an efficient sorbent in microextraction by packed sorbent: Determination of local anesthetics in human plasma and saliva samples utilizing liquid chromatography-tandem mass spectrometry. *J. Chromatogr. B* **2018**, *1095*, 177-182.
25. Fuentes, A.M.A.; Fernández, P.; Fernández, A.M.; Carro, A.M.; Lorenzo, R.A. Microextraction by packed sorbent followed by ultra high performance liquid chromatography for the fast extraction and determination of six antidepressants in urine. *J. Sep. Sci.* **2019**, *42*, 2053-2061, doi:10.1002/jssc.201900060.
26. Nuckowski, Ł.; Kaczmarkiewicz, A.; Studzińska, S.; Buszewski, B. A new approach to preparation of antisense oligonucleotide samples with microextraction by packed sorbent. *Analyst* **2019**, *144*, 4622-4632, doi:10.1039/c9an00740g.
27. D'Angelo, V.; Tessari, F.; Bellagamba, G.; De Luca, E.; Cifelli, R.; Celia, C.; Primavera, R.; Di Francesco, M.; Paolino, D.; Di Marzio, L., et al. Microextraction by packed sorbent and HPLC-PDA quantification of multiple anti-inflammatory drugs and fluoroquinolones in human plasma and urine. *J. Enzyme Inhib. Med. Chem.* **2016**, *31*, 110-116, doi:10.1080/14756366.2016.1209496.
28. Berenguer, P.H.; Camacho, I.C.; Câmara, R.; Oliveira, S.; Câmara, J.S. Determination of potential childhood asthma biomarkers using a powerful methodology based on microextraction by packed sorbent combined with ultra-high pressure liquid chromatography. Eicosanoids as case study. *J. Chromatogr. A* **2019**, *1584*, 42-56, doi:10.1016/j.chroma.2018.11.041.
29. Campestre, C.; Locatelli, M.; Guglielmi, P.; De Luca, E.; Bellagamba, G.; Menta, S.; Zengin, G.; Celia, C.; Di Marzio, L.; Carradori, S. Analysis of imidazoles and triazoles in biological samples after MicroExtraction by packed sorbent. *J. Enzyme Inhib. Med. Chem.* **2017**, *32*, 1053-1063, doi:10.1080/14756366.2017.1354858.
30. Ventura, S.; Rodrigues, M.; Falcão, A.; Alves, G. Short-term effects of Garcinia cambogia extract on the pharmacokinetics of lamotrigine given as a single-dose in Wistar rats. *Food Chem. Toxicol.* **2019**, *128*, 61-67, doi:10.1016/j.fct.2019.03.051.
31. Meng, J.; Wang, X. Microextraction by packed molecularly imprinted polymer combined ultra-high-performance liquid chromatography for the determination of levofloxacin in human plasma. *Journal of Chemistry* **2019**, *2019*, doi:10.1155/2019/4783432.
32. Rahimpoor, R.; Bahrami, A.; Nematollahi, D.; Shahna, F.G.; Farhadian, M. Facile and sensitive determination of urinary mandelic acid by combination of metal organic frameworks with microextraction by packed sorbents. *Journal of Chromatography B: Analytical Technologies in the Biomedical and Life Sciences* **2019**, *1114-1115*, 45-54, doi:10.1016/j.jchromb.2019.03.023.
33. Rasolzadeh, F.; Hashemi, P.; Haghjou, M.M.; Safdarian, M. Chlorella vulgaris microalgae as a green packing for the microextraction by packed sorbent of nitrofurantoin in urine. *Analytical and Bioanalytical Chemistry Research* **2019**, *6*, 419-429.
34. Prata, M.; Ribeiro, A.; Figueirinha, D.; Rosado, T.; Oppolzer, D.; Restolho, J.; Araújo, A.R.T.S.; Costa, S.; Barroso, M.; Gallardo, E. Determination of opiates in whole blood using microextraction by packed sorbent and gas chromatography-tandem mass spectrometry. *J. Chromatogr. A* **2019**, *1602*, 1-10, doi:10.1016/j.chroma.2019.05.021.
35. Zhang, J.; Liu, D.; Meng, X.; Shi, Y.; Wang, R.; Xiao, D.; He, H. Solid phase extraction based on porous magnetic graphene oxide/beta-cyclodextrine composite coupled with high performance liquid chromatography for determination of antiepileptic drugs in plasma samples. *J. Chromatogr. A* **2017**, *1524*, 49-56, doi:10.1016/j.chroma.2017.09.074.
36. Peng, J.; Tian, H.R.; Du, Q.Z.; Hui, X.H.; He, H. A regenerable sorbent composed of a zeolite imidazolate framework (ZIF-8), Fe3O4 and graphene oxide for enrichment of atorvastatin and simvastatin prior to their determination by HPLC. *Microchimica Acta* **2018**, *185*, 1-9, doi:10.1007/s00604-018-2697-6.
37. Yuvali, D.; Narin, I.; Soylak, M.; Yilmaz, E. Green synthesis of magnetic carbon nanodot/graphene oxide hybrid material (Fe3O4@C-nanodot@GO) for magnetic solid phase extraction of ibuprofen in human blood samples prior to HPLC-DAD determination. *J. Pharm. Biomed. Anal.* **2020**, *179*, 113001, doi:10.1016/j.jpba.2019.113001.
38. Barati, A.; Kazemi, E.; Dadfarnia, S.; Shabani, A.M.H. Synthesis/characterization of molecular imprinted polymer based on magnetic chitosan/graphene oxide for selective separation/preconcentration of fluoxetine from environmental and biological samples. *Journal of Industrial and Engineering Chemistry* **2017**, *46*, 212-221, doi:10.1016/j.jiec.2016.10.033.
39. Lamei, N.; Ezoddin, M.; Ardestani, M.S.; Abdi, K. Dispersion of magnetic graphene oxide nanoparticles coated with a deep eutectic solvent using ultrasound assistance for preconcentration of methadone in biological and water samples followed by GC–FID and GC–MS. *Anal. Bioanal. Chem.* **2017**, *409*, 6113-6121, doi:10.1007/s00216-017-0547-8.
40. Asgharinezhad, A.A.; Ebrahimzadeh, H. Poly(2-aminobenzothiazole)-coated graphene oxide/magnetite nanoparticles composite as an efficient sorbent for determination of non-steroidal anti-inflammatory drugs in urine sample. *J. Chromatogr. A* **2016**, *1435*, 18-29, doi:10.1016/j.chroma.2016.01.027.

41. Abdolmohammad-Zadeh, H.; Zamani, A.; Shamsi, Z. Extraction of four endocrine-disrupting chemicals using a Fe3O4/graphene oxide/di-(2-ethylhexyl) phosphoric acid nano-composite, and their quantification by HPLC-UV. *Microchem. J.* **2020**, *157*, 104964.
42. Taghvimi, A.; Hamishehkar, H.; Ebrahimi, M. Magnetic nano graphene oxide as solid phase extraction adsorbent coupled with liquid chromatography to determine pseudoephedrine in urine samples. *Journal of Chromatography B-Analytical Technologies in the Biomedical and Life Sciences* **2016**, *1009*, 66-72, doi:10.1016/j.jchromb.2015.12.005.
43. Taghvimi, A.; Hamishehkar, H. Developed nano carbon-based coating for simultaneous extraction of potent central nervous system stimulants from urine media by stir bar sorptive extraction method coupled to high performance liquid chromatography. *J. Chromatogr. B Analyt. Technol. Biomed. Life. Sci.* **2019**, *1125*, 121701, doi:10.1016/j.jchromb.2019.06.028.
44. Taghizadeh, M.; Ebrahimi, M.; Fooladi, E.; Yoosefian, M. Preconcentration and determination of five antidepressants from human milk and urine samples by stir bar filled magnetic ionic liquids using liquid-liquid-liquid microextraction-high performance liquid chromatography. *J. Sep. Sci.* **2022**, 10.1002/jssc.202100617, doi:10.1002/jssc.202100617.
45. Mohammadi, P.; Masrournia, M.; Es'haghi, Z.; Pordel, M. Hollow fiber coated Fe3O4@ Maleamic acid-functionalized graphene oxide as a sorbent for stir bar sorptive extraction of ibuprofen, aspirin, and venlafaxine in human urine samples before determining by gas chromatography–mass spectrometry. *Journal of the Iranian Chemical Society* **2021**, *18*, 2249-2259.
46. Babarahimi, V.; Talebpour, Z.; Haghighi, F.; Adib, N.; Vahidi, H. Validated determination of losartan and valsartan in human plasma by stir bar sorptive extraction based on acrylate monolithic polymer, liquid chromatographic analysis and experimental design methodology. *J. Pharm. Biomed. Anal.* **2018**, *153*, 204-213.
47. Fan, W.; He, M.; You, L.; Zhu, X.; Chen, B.; Hu, B. Water-compatible graphene oxide/molecularly imprinted polymer coated stir bar sorptive extraction of propranolol from urine samples followed by high performance liquid chromatography-ultraviolet detection. *J. Chromatogr. A* **2016**, *1443*, 1-9.
48. Jillani, S.M.S.; Ganiyu, S.A.; Alhooshani, K. Development of a SBSE-HPLC method using sol-gel based germania coated twister for the analysis of 4-chloro-1-naphthol in biological and water samples. *Arabian Journal of Chemistry* **2020**, *13*, 3440-3447.
49. Yuan, Y.N.; Sun, N.; Yan, H.Y.; Han, D.D.; Row, K.H. Determination of indometacin and acemetacin in human urine via reduced graphene oxide - based pipette tip solid-phase extraction coupled to HPLC. *Microchimica Acta* **2016**, *183*, 799-804, doi:10.1007/s00604-015-1711-5.
50. Santos da Silva, R.C.; Mano, V.; Pereira, A.C.; Costa de Figueiredo, E.; Borges, K.B. Development of pipette tip-based on molecularly imprinted polymer micro-solid phase extraction for selective enantioselective determination of (−)-(2S,4R) and (+)-(2R,4S) ketoconazole in human urine samples prior to HPLC-DAD. *Analytical Methods* **2016**, *8*, 4075-4085, doi:10.1039/C6AY00392C.
51. Zhang, Y.; Zhao, Y.-G.; Chen, W.-S.; Cheng, H.-L.; Zeng, X.-Q.; Zhu, Y. Three-dimensional ionic liquid-ferrite functionalized graphene oxide nanocomposite for pipette-tip solid phase extraction of 16 polycyclic aromatic hydrocarbons in human blood sample. *J. Chromatogr. A* **2018**, *1552*, 1-9.
52. Yuan, Y.; Han, Y.; Yang, C.; Han, D.; Yan, H. Deep eutectic solvent functionalized graphene oxide composite adsorbent for miniaturized pipette-tip solid-phase extraction of toluene and xylene exposure biomarkers in urine prior to their determination with HPLC-UV. *Mikrochim. Acta* **2020**, *187*, 387, doi:10.1007/s00604-020-04370-z.
53. Guedes-Alonso, R.; Ciofi, L.; Sosa-Ferrera, Z.; Santana-Rodríguez, J.J.; Bubba, M.D.; Kabir, A.; Furton, K.G. Determination of androgens and progestogens in environmental and biological samples using fabric phase sorptive extraction coupled to ultra-high performance liquid chromatography tandem mass spectrometry. *J. Chromatogr. A* **2016**, *1437*, 116-126, doi:10.1016/j.chroma.2016.01.077.
54. Locatelli, M.; Tinari, N.; Grassadonia, A.; Tartaglia, A.; Macerola, D.; Piccolantonio, S.; Sperandio, E.; D'Ovidio, C.; Carradori, S.; Ulusoy, H.I., et al. FPSE-HPLC-DAD method for the quantification of anticancer drugs in human whole blood, plasma, and urine. *J. Chromatogr. B Analyt. Technol. Biomed. Life. Sci.* **2018**, *1095*, 204-213, doi:10.1016/j.jchromb.2018.07.042.
55. Lioupi, A.; Kabir, A.; Furton, K.G.; Samanidou, V. Fabric phase sorptive extraction for the isolation of five common antidepressants from human urine prior to HPLC-DAD analysis. *J. Chromatogr. B Analyt. Technol. Biomed. Life. Sci.* **2019**, *1118-1119*, 171-179, doi:10.1016/j.jchromb.2019.04.045.
56. Locatelli, M.; Kabir, A.; Innosa, D.; Lopatriello, T.; Furton, K.G. A fabric phase sorptive extraction-High performance liquid chromatography-Photo diode array detection method for the determination of twelve azole antimicrobial drug residues in human plasma and urine. *J. Chromatogr. B Analyt. Technol. Biomed. Life. Sci.* **2017**, *1040*, 192-198, doi:10.1016/j.jchromb.2016.10.045.
57. Samanidou, V.; Kaltzi, I.; Kabir, A.; Furton, K.G. Simplifying sample preparation using fabric phase sorptive extraction technique for the determination of benzodiazepines in blood serum by high-performance liquid chromatography. *Biomed. Chromatogr.* **2016**, *30*, 829-836, doi:10.1002/bmc.3615.
58. Samanidou, V.; Filippou, O.; Marinou, E.; Kabir, A.; Furton, K.G. Sol-gel-graphene-based fabric-phase sorptive extraction for cow and human breast milk sample cleanup for screening bisphenol A and residual dental restorative material before analysis by HPLC with diode array detection. *J. Sep. Sci.* **2017**, *40*, 2612-2619, doi:10.1002/jssc.201700256.
59. Kazantzi, V.; Samanidou, V.; Kabir, A.; Furton, K.G.; Anthemidis, A. On-Line Fabric Disk Sorptive Extraction via a Flow Preconcentration Platform Coupled with Atomic Absorption Spectrometry for the Determination of Essential and Toxic Elements in Biological Samples. *Separations* **2018**, *5*, 34.

60. Kabir, A.; Furton, K.G.; Tinari, N.; Grossi, L.; Innosa, D.; Macerola, D.; Tartaglia, A.; Di Donato, V.; D'Ovidio, C.; Locatelli, M. Fabric phase sorptive extraction-high performance liquid chromatography-photo diode array detection method for simultaneous monitoring of three inflammatory bowel disease treatment drugs in whole blood, plasma and urine. *J. Chromatogr. B Analyt. Technol. Biomed. Life. Sci.* **2018**, *1084*, 53-63, doi:10.1016/j.jchromb.2018.03.028.
61. Moreira, B.J.; Schiave, L.A.; Martinez, R.; Dias, S.G.; Masetto de Gaitani, C. Dispersive liquid–liquid microextraction followed by green high-performance liquid chromatography for fluconazole determination in cerebrospinal fluid with the aid of chemometric tools. *Analytical Methods* **2020**, *12*, 3106-3114, doi:10.1039/D0AY00704H.
62. Tartaglia, A.; Kabir, A.; Ulusoy, S.; Sperandio, E.; Piccolantonio, S.; Ulusoy, H.I.; Furton, K.G.; Locatelli, M. FPSE-HPLC-PDA analysis of seven paraben residues in human whole blood, plasma, and urine. *J. Chromatogr. B Analyt. Technol. Biomed. Life. Sci.* **2019**, *1125*, 121707, doi:10.1016/j.jchromb.2019.06.034.
63. Taraboletti, A.; Goudarzi, M.; Kabir, A.; Moon, B.H.; Laiakis, E.C.; Lacombe, J.; Ake, P.; Shoishiro, S.; Brenner, D.; Fornace, A.J., Jr., et al. Fabric Phase Sorptive Extraction-A Metabolomic Preprocessing Approach for Ionizing Radiation Exposure Assessment. *J. Proteome Res.* **2019**, *18*, 3020-3031, doi:10.1021/acs.jproteome.9b00142.
64. Riffi, M.; Fabresse, N.; Abe, E.; Knapp, A.; Etting, I.; Alvarez, J.-C.; Larabi, I.A. Mise au point et validation d'une méthode de micro-extraction liquide-liquide dispersée appliquée au dosage de 7 neuroleptiques. Comparaison avec une méthode d'extraction liquide-liquide conventionnelle et application. *Toxicologie Analytique et Clinique* **2018**, *30*, 96-105.
65. Temerdashev, A.; Dmitrieva, E.; Azaryan, A.; Gashimova, E. A novel approach to the quantification of urinary aryl-propionamide-derived SARMs by UHPLC–MS/MS. *Biomed. Chromatogr.* **2020**, *34*, e4700.
66. De Boeck, M.; Missotten, S.; Dehaen, W.; Tytgat, J.; Cuypers, E. Development and validation of a fast ionic liquid-based dispersive liquid-liquid microextraction procedure combined with LC-MS/MS analysis for the quantification of benzodiazepines and benzodiazepine-like hypnotics in whole blood. *Forensic Sci. Int.* **2017**, *274*, 44-54, doi:10.1016/j.forsciint.2016.12.026.
67. Ranjbar, S.; Daryasari, A.P.; Soleimani, M. Ionic Liquid-Based Dispersive Liquidaeuro"Liquid Microextraction for the Simultaneous Determination of Carbamazepine and Lamotrigine in Biological Samples. *Acta Chim Slov* **2020**, *67*, 748-756.
68. Mabrouk, M.M.; Soliman, S.M.; El-Agizy, H.M.; Mansour, F.R. Ultrasound-assisted dispersive liquid–liquid microextraction for determination of three gliflozins in human plasma by HPLC/DAD. *J. Chromatogr. B* **2020**, *1136*, 121932, doi:10.1016/j.jchromb.2019.121932.
69. Hamidi, S.; Azami, A.; Mehdizadeh Aghdam, E. A novel mixed hemimicelles dispersive micro-solid phase extraction using ionic liquid functionalized magnetic graphene oxide/polypyrrole for extraction and pre-concentration of methotrexate from urine samples followed by the spectrophotometric method. *Clin. Chim. Acta* **2019**, *488*, 179-188, doi:10.1016/j.cca.2018.11.006.
70. Zhou, G.-S.; Yuan, Y.-C.; Yin, Y.; Tang, Y.-P.; Xu, R.-J.; Liu, Y.; Chen, P.-D.; Yin, L.; Duan, J.-A. Hydrophilic interaction chromatography combined with ultrasound-assisted ionic liquid dispersive liquid–liquid microextraction for determination of underivatized neurotransmitters in dementia patients' urine samples. *Anal. Chim. Acta* **2020**, *1107*, 74-84.
71. Shirkhanloo, H.; Karamzadeh, Z.; Rakhtshah, J.; Kazemi, N.M. A novel biostructure sorbent based on CysSB/MetSB@MWCNTs for separation of nickel and cobalt in biological samples by ultrasound assisted-dispersive ionic liquid-suspension solid phase micro extraction. *J. Pharm. Biomed. Anal.* **2019**, *172*, 285-294, doi:10.1016/j.jpba.2019.05.003.
72. Talpur, S.; Kazi, T.G.; Afridi, H.I.; Talpur, F.N.; Nizamani, S.; Lashari, A.; Akhtar, A.; Khan, M. Ultrasonically Dispersed Ionic Liquid-Based Microextraction of Lead in Biological Samples of Malnourished Children Prior to Analysis by Flame Atomic Absorption Spectrometry. *J. AOAC Int.* **2018**, *101*, 883-890, doi:10.5740/jaoacint.17-0269.
73. Kong, L.; Wang, J.; Gao, Q.; Li, X.; Zhang, W.; Wang, P.; Ma, L.; He, L. Simultaneous determination of fat-soluble vitamins and carotenoids in human serum using a nanostructured ionic liquid based microextraction method. *J. Chromatogr. A* **2022**, *1666*, 462861, doi:10.1016/j.chroma.2022.462861.
74. Iqbal, M.; Ezzeldin, E.; Khalil, N.Y.; Alam, P.; Al-Rashood, K.A. UPLC-MS/MS determination of suvorexant in urine by a simplified dispersive liquid-liquid micro-extraction followed by ultrasound assisted back extraction from solidified floating organic droplets. *J. Pharm. Biomed. Anal.* **2019**, *164*, 1-8.
75. Abbaszadeh, S.; Yousefinejad, S.; Jafari, S.; Soleimani, E. In-syringe ionic liquid-dispersive liquid-liquid microextraction coupled with HPLC for the determination of trans,trans-muconic acid in human urine sample. *J. Sep. Sci.* **2021**, *44*, 3126-3136, doi:10.1002/jssc.202100044.
76. Correia-Sa, L.; Norberto, S.; Delerue-Matos, C.; Calhau, C.; Domingues, V.F. Micro-QuEChERS extraction coupled to GC-MS for a fast determination of Bisphenol A in human urine. *J. Chromatogr. B Analyt. Technol. Biomed. Life. Sci.* **2018**, *1072*, 9-16, doi:10.1016/j.jchromb.2017.10.060.
77. Alves, V.; Conceição, C.; Gonçalves, J.; Teixeira, H.M.; Câmara, J.S. Improved Analytical Approach Based on QuECHERS/UHPLC-PDA for Quantification of Fluoxetine, Clomipramine and their Active Metabolites in Human Urine Samples. *J. Anal. Toxicol.* **2017**, *41*, 45-53, doi:10.1093/jat/bkw077.
78. Pouliopoulos, A.; Tsakelidou, E.; Krokos, A.; Gika, H.G.; Theodoridis, G.; Raikos, N. Quantification of 15 Psychotropic Drugs in Serum and Postmortem Blood Samples after a Modified Mini-QuEChERS by UHPLC–MS-MS. *J. Anal. Toxicol.* **2018**, *42*, 337-345, doi:10.1093/jat/bky006.

79. Rial-Berriel, C.; Acosta-Dacal, A.; Zumbado, M.; Luzardo, O.P. Micro QuEChERS-based method for the simultaneous biomonitoring in whole blood of 360 toxicologically relevant pollutants for wildlife. *Sci. Total Environ.* **2020**, *736*, 139444, doi:10.1016/j.scitotenv.2020.139444.
80. Passos, C.P.; Petronilho, S.; Serodio, A.F.; Neto, A.C.M.; Torres, D.; Rudnitskaya, A.; Nunes, C.; Kukurova, K.; Ciesarova, Z.; Rocha, S.M., et al. HS-SPME Gas Chromatography Approach for Underivatized Acrylamide Determination in Biscuits. *Foods* **2021**, *10*, 1-13, doi:10.3390/foods10092183.
81. Pang, L.; Yang, P.; Pang, R.; Lu, X.; Xiao, J.; Li, S.; Zhang, H.; Zhao, J. Ionogel-based ionic liquid coating for solid-phase microextraction of organophosphorus pesticides from wine and juice samples. *Food Analytical Methods* **2018**, *11*, 270-281, doi:10.1007/s12161-017-0997-9.
82. Haghi, J.N.; Husain, S.W.; Azar, P.A.; Tehran, M.S. Fabrication of silica nanoparticle-PEG-ionic liquid SPME fiber for determination of pesticide residues in tomato. *Iioab* **2017**, *8*, 11-17.
83. Perestrelo, R.; Silva, C.L.; Algarra, M.; Câmara, J.S. Evaluation of the occurrence of phthalates in plastic materials used in food packaging. *Applied Sciences* **2021**, *11*, 2130-2141, doi:10.3390/app11052130.
84. Perestrelo, R.; Silva, C.L.; Algarra, M.; Câmara, J.S. Monitoring phthalates in table and fortified wines by headspace solid-phase microextraction combined with gas chromatography-mass spectrometry analysis. *J. Agric. Food. Chem.* **2020**, *68*, 8431-8437, doi:10.1021/acs.jafc.0c02941.
85. Chen, Y.; Zhang, Y.; Xu, L. A rapid method for analyzing synthetic phenolic antioxidants in food grade lubricant samples based on headspace solid-phase microextracion coupled with gas chromatography-mass spectrometer. *Food Analytical Methods* **2021**, 10.1007/s12161-021-02053-w, doi:10.1007/s12161-021-02053-w.
86. Kalogiouri, N.P.; Manousi, N.; Rosenberg, E.; Zachariadis, G.A.; Paraskevopoulou, A.; Samanidou, V. Exploring the volatile metabolome of conventional and organic walnut oils by solid-phase microextraction and analysis by GC-MS combined with chemometrics. *Food Chem.* **2021**, *363*, 130331-130331, doi:10.1016/j.foodchem.2021.130331.
87. Zhao, C.C.; Eun, J.B. Characterization of volatile compounds and physicochemical properties of hongeo using headspace solid-phase microextraction and gas chromatography-mass spectrometry during fermentation. *Food Bioscience* **2021**, *44*, 101379-101379, doi:10.1016/j.fbio.2021.101379.
88. Majithia, D.; Metrani, R.; Dhowlaghar, N.; Crosby, K.M.; Patil, B.S. Assessment and classification of volatile profiles in melon breeding lines using headspace solid-phase microextraction coupled with gas chromatography-mass spectrometry. *Plants* **2021**, *10*, doi:10.3390/plants10102166.
89. Thomas, C.F.; Zeh, E.; Dörfel, S.; Zhang, Y.; Hinrichs, J. Studying dynamic aroma release by headspace-solid phase microextraction-gas chromatography-ion mobility spectrometry (HS-SPME-GC-IMS): method optimization, validation, and application. *Anal. Bioanal. Chem.* **2021**, *413*, 2577-2586, doi:10.1007/s00216-021-03222-w.
90. Mejía-Carmona, K.; Lanças, F.M. Modified graphene-silica as a sorbent for in-tube solid-phase microextraction coupled to liquid chromatography-tandem mass spectrometry. Determination of xanthines in coffee beverages. *J. Chromatogr. A* **2020**, *1621*, doi:10.1016/j.chroma.2020.461089.
91. He, X.; Majid, B.; Zhang, H.; Liu, W.; Limmer, M.A.; Burken, J.G.; Shi, H. Green analysis: Rapid-throughput analysis of volatile contaminants in plants by freeze-thaw-equilibration sample preparation and SPME-GC-MS analysis. *J. Agric. Food. Chem.* **2021**, *69*, 5428-5434, doi:10.1021/acs.jafc.1c01497.
92. Zianni, R.; Mentana, A.; Campaniello, M.; Chiappinelli, A.; Tomaiuolo, M.; Chiaravalle, A.E.; Marchesani, G. An investigation using a validated method based on HS-SPME-GC-MS detection for the determination of 2-dodecylcyclobutanone and 2-tetradecylcyclobutanone in X-ray irradiated dairy products. *Lwt* **2022**, *153*, 112466-112466, doi:10.1016/j.lwt.2021.112466.
93. Li, G.; Row, K.H. Selective extraction of 3,4-dihydroxybenzoic acid in Ilex chinensis Sims by meticulous mini-solid-phase microextraction using ternary deep eutectic solvent-based molecularly imprinted polymers. *Anal. Bioanal. Chem.* **2018**, *410*, 7849-7858, doi:10.1007/S00216-018-1406-Y/TABLES/1.
94. Chmangui, A.; Jayasinghe, G.D.T.M.; Driss, M.R.; Touil, S.; Bermejo-Barrera, P.; Bouabdallah, S.; Moreda-Piñeiro, A. Assessment of trace levels of aflatoxins AFB1 and AFB2 in non-dairy beverages by molecularly imprinted polymer based micro solid-phase extraction and liquid chromatography-tandem mass spectrometry. *Analytical Methods* **2021**, *13*, 3433-3443, doi:10.1039/D1AY00793A.
95. Kaykhaii, M.; Yavari, E.; Sargazi, G.; Ebrahimi, A.K. Highly Sensitive Determination of Bisphenol A in Bottled Water Samples by HPLC after Its Extraction by a Novel Th-MOF Pipette-Tip Micro-SPE. *J. Chromatogr. Sci.* **2020**, *58*, 373-382, doi:10.1093/chromsci/bmz111.
96. Amini, S.; Ebrahimzadeh, H.; Seidi, S.; Jalilian, N. Application of electrospun polyacrylonitrile/Zn-MOF-74@GO nanocomposite as the sorbent for online micro solid-phase extraction of chlorobenzenes in water, soil, and food samples prior to liquid chromatography analysis. *Food Chem.* **2021**, *363*, 130330-130330, doi:10.1016/J.FOODCHEM.2021.130330.
97. Khiltash, S.; Heydari, R.; Ramezani, M. Graphene oxide/polydopamine-polyacrylamide nanocomposite as a sorbent for dispersive micro-solid phase extraction of diazinon from environmental and food samples and its determination by HPLC-UV detection. *10.1080/03067319.2021.1971211* **2021**, 10.1080/03067319.2021.1971211, doi:10.1080/03067319.2021.1971211.

98. Tang, M.; Zhao, Y.; Chen, J.; Xu, D. On-line multi-residue analysis of fluoroquinolones and amantadine based on an integrated microfluidic chip coupled to triple quadrupole mass spectrometry. *Analytical Methods* **2020**, *12*, 5322-5331, doi:10.1039/d0ay01641a.
99. Han, L.; Sapozhnikova, Y. Semi-automated high-throughput method for residual analysis of 302 pesticides and environmental contaminants in catfish by fast low-pressure GC–MS/MS and UHPLC-MS/MS. *Food Chem.* **2020**, *319*, 126592-126592, doi:10.1016/j.foodchem.2020.126592.
100. Xia, L.; He, Y.; Xiao, X.; Li, G. An online field-assisted micro-solid-phase extraction device coupled with high-performance liquid chromatography for the direct analysis of solid samples. *Anal. Bioanal. Chem.* **2019**, *411*, 4073-4084, doi:10.1007/s00216-019-01809-y.
101. Atirah Mohd Nazir, N.; Raoov, M.; Mohamad, S. Spent tea leaves as an adsorbent for micro-solid-phase extraction of polycyclic aromatic hydrocarbons (PAHs) from water and food samples prior to GC-FID analysis. *Microchem. J.* **2020**, *159*, 105581, doi:10.1016/j.microc.2020.105581.
102. Eyring, P.; Tienstra, M.; Mol, H.; Herrmann, S.S.; Rasmussen, P.H.; Frandsen, H.L.; Poulsen, M.E. Development of a new generic extraction method for the analysis of pesticides, mycotoxins, and polycyclic aromatic hydrocarbons in representative animal feed and food samples. *Food Chem.* **2021**, *356*, 129653-129653, doi:10.1016/J.FOODCHEM.2021.129653.
103. Nasrollahpour, A.; Moradi, S.E.; Baniamerian, M.J. Vortex-Assisted Dispersive Solid-Phase Microextraction Using Ionic Liquid-Modified Metal-Organic Frameworks of PAHs from Environmental Water, Vegetable, and Fruit Juice Samples. *Food Analytical Methods* **2017**, *10*, 2815-2826, doi:10.1007/s12161-017-0843-0.
104. Hakme, E.; Poulsen, M.E. Evaluation of the automated micro-solid phase extraction clean-up system for the analysis of pesticide residues in cereals by gas chromatography-Orbitrap mass spectrometry. *J. Chromatogr. A* **2021**, *1652*, 462384-462384, doi:10.1016/J.CHROMA.2021.462384.
105. Nooraee Nia, N.; Reza Hadjmohammadi, M. Development of magnetic dispersive micro-solid phase extraction based on magnetic adipic acid nanoparticles and deep eutectic solvents for the isolation and pre-concentration of phenolic compounds in fruit juice samples prior to determination by HPLC-UV. *Microchem. J.* **2021**, *170*, 106721-106721, doi:10.1016/j.microc.2021.106721.
106. Alipanahpour Dil, E.; Asfaram, A.; Goudarzi, A.; Zabihi, E.; Javadian, H. Biocompatible chitosan-zinc oxide nanocomposite based dispersive micro-solid phase extraction coupled with HPLC-UV for the determination of rosmarinic acid in the extracts of medical plants and water sample. *Int. J. Biol. Macromol.* **2020**, *154*, 528-537, doi:10.1016/J.IJBIOMAC.2020.03.132.
107. Kamali, S.R.; Tsai, C.H.; Chen, C.N. Pipette Tip Solid-Phase Extraction Combined with Fluorescence Spectroscopy for Determination of Selenium in Green Tea Samples. *IOP Conference Series: Earth and Environmental Science* **2021**, *913*, 012064, doi:10.1088/1755-1315/913/1/012064.
108. Nyaba, L.; Nomngongo, P.N. Determination of trace metals in vegetables and water samples using dispersive ultrasound-assisted cloud point-dispersive μ-solid phase extraction coupled with inductively coupled plasma optical emission spectrometry. *Food Chem.* **2020**, *322*, 126749, doi:10.1016/j.foodchem.2020.126749.
109. Sereshti, H.; Toloutehrani, A.; Nodeh, H.R. Determination of cholecalciferol (vitamin D3) in bovine milk by dispersive micro-solid phase extraction based on the magnetic three-dimensional graphene-sporopollenin sorbent. *J. Chromatogr. B* **2020**, *1136*, 121907-121907, doi:10.1016/J.JCHROMB.2019.121907.
110. Teixeira, L.S.; Silva, C.F.; de Oliveira, H.L.; Dinali, L.A.F.; Nascimento, C.S.; Borges, K.B. Microextraction by packed molecularly imprinted polymer to selectively determine caffeine in soft and energy drinks. *Microchem. J.* **2020**, *158*, 105252, doi:10.1016/j.microc.2020.105252.
111. Aresta, A.; Cotugno, P.; Zambonin, C. Determination of ciprofloxacin, enrofloxacin, and marbofloxacin in bovine urine, serum, and milk by microextraction by a packed sorbent coupled to ultra-high performance liquid chromatography. *Anal. Lett.* **2019**, *52*, 790-802, doi:10.1080/00032719.2018.1496093.
112. Mousavi, K.Z.; Yamini, Y.; Karimi, B.; Seidi, S.; Khorasani, M.; Ghaemmaghami, M.; Vali, H. Imidazolium-based mesoporous organosilicas with bridging organic groups for microextraction by packed sorbent of phenoxy acid herbicides, polycyclic aromatic hydrocarbons and chlorophenols. *Microchimica Acta* **2019**, *186*, doi:10.1007/s00604-019-3355-3.
113. Teixeira, R.A.; Dinali, L.A.F.; Silva, C.F.; de Oliveira, H.L.; da Silva, A.T.M.; Nascimento, C.S.; Borges, K.B. Microextraction by packed molecularly imprinted polymer followed by ultra-high performance liquid chromatography for determination of fipronil and fluazuron residues in drinking water and veterinary clinic wastewater. *Microchem. J.* **2021**, *168*, 106405, doi:10.1016/j.microc.2021.106405.
114. Jordan-Sinisterra, M.; Lanças, F.M. Microextraction by packed sorbent of selected pesticides in coffee samples employing ionic liquids supported on graphene nanosheets as extraction phase. *Anal. Bioanal. Chem.* **2022**, *414*, 413-423, doi:10.1007/s00216-021-03245-3.
115. Jiang, Y.; Qin, Z.; Song, X.; Piao, H.; Li, J.; Wang, X.; Song, D.; Ma, P.; Sun, Y. Facile preparation of metal organic framework-based laboratory semi-automatic micro-extraction syringe packed column for analysis of parabens in vegetable oil samples. *Microchem. J.* **2020**, *158*, 105200-105200, doi:10.1016/j.microc.2020.105200.

116. Dinali, L.A.F.; de Oliveira, H.L.; Teixeira, L.S.; de Souza Borges, W.; Borges, K.B. Mesoporous molecularly imprinted polymer core@shell hybrid silica nanoparticles as adsorbent in microextraction by packed sorbent for multiresidue determination of pesticides in apple juice. *Food Chem.* **2021**, *345*, 128745-128745, doi:10.1016/j.foodchem.2020.128745.
117. Paris, A.; Gaillard, J.L.; Ledauphin, J. Rapid extraction of polycyclic aromatic hydrocarbons in apple: ultrasound-assisted solvent extraction followed by microextraction by packed sorbent. *Food Analytical Methods* **2019**, *12*, 2194-2204, doi:10.1007/s12161-019-01568-7.
118. Casado, N.; Perestrelo, R.; Silva, C.L.; Sierra, I.; Câmara, J.S. Comparison of high-throughput microextraction techniques, MEPS and μ-SPEed, for the determination of polyphenols in baby food by ultrahigh pressure liquid chromatography. *Food Chem.* **2019**, *292*, 14-23, doi:10.1016/j.foodchem.2019.04.038.
119. Florez, D.H.Â.; de Oliveira, H.L.; Borges, K.B. Polythiophene as highly efficient sorbent for microextraction in packed sorbent for determination of steroids from bovine milk samples. *Microchem. J.* **2020**, *153*, 104521-104521, doi:10.1016/j.microc.2019.104521.
120. Souza, M.C.O.; Rocha, B.A.; Souza, J.M.O.; Berretta, A.A.; Barbosa, F. A Fast and simple procedure for polybrominated diphenyl ether determination in egg samples by using microextraction by packed sorbent and gas chromatography–mass spectrometry. *Food Analytical Methods* **2019**, *12*, 1528-1535, doi:10.1007/s12161-019-01484-w.
121. Canadas, R.; Garrido Gamarro, E.; Garcinuno Martinez, R.M.; Paniagua Gonzalez, G.; Fernandez Hernando, P. Occurrence of common plastic additives and contaminants in mussel samples: Validation of analytical method based on matrix solid-phase dispersion. *Food Chem.* **2021**, *349*, 129169, doi:10.1016/j.foodchem.2021.129169.
122. Qian, Z.; Wu, Z.; Li, C.; Tan, G.; Hu, H.; Li, W. A green liquid chromatography method for rapid determination of ergosterol in edible fungi based on matrix solid-phase dispersion extraction and a core-shell column. *Analytical Methods* **2020**, *12*, 3327-3343, doi:10.1039/d0ay00714e.
123. Mansur, A.R.; Kim, K.J.; Kim, D.B.; Yoo, M.; Jang, H.W.; Kim, D.O.; Nam, T.G. Matrix solid-phase dispersion extraction method for HPLC determination of flavonoids from buckwheat sprouts. *Lwt* **2020**, *133*, 110121-110121, doi:10.1016/j.lwt.2020.110121.
124. Peng, L.Q.; Zhang, Y.; Yan, T.C.; Gu, Y.X.; Zi, X.; Cao, J. Carbonized biosorbent assisted matrix solid-phase dispersion microextraction for active compounds from functional food. *Food Chem.* **2021**, *365*, 130545-130545, doi:10.1016/j.foodchem.2021.130545.
125. Segatto, M.L.; Zanotti, K.; Zuin, V.G. Microwave-assisted extraction and matrix solid-phase dispersion as green analytical chemistry sample preparation techniques for the valorisation of mango processing waste. *Current Research in Chemical Biology* **2021**, *1*, 100007-100007, doi:10.1016/j.crchbi.2021.100007.
126. Santos Barreto, A.; de Cássia da Silva Andrade, P.; Meira Farias, J.; Menezes Filho, A.; Fernandes de Sá, G.; Alves Júnior, S. Characterization and application of a lanthanide-based metal–organic framework in the development and validation of a matrix solid-phase dispersion procedure for pesticide extraction on peppers (Capsicum annuum L.) with gas chromatography–mass spectrometr. *J. Sep. Sci.* **2018**, *41*, 1593-1599, doi:10.1002/jssc.201700812.
127. Souza, M.R.R.; Jesus, R.A.; Costa, J.A.S.; Barreto, A.S.; Navickiene, S.; Mesquita, M.E. Applicability of metal–organic framework materials in the evaluation of pesticide residues in egg samples of chicken (Gallus gallus domesticus). *Journal fur Verbraucherschutz und Lebensmittelsicherheit* **2021**, *16*, 83-91, doi:10.1007/s00003-020-01304-y.
128. Chatzimitakos, T.G.; Anderson, J.L.; Stalikas, C.D. Matrix solid-phase dispersion based on magnetic ionic liquids: An alternative sample preparation approach for the extraction of pesticides from vegetables. *J. Chromatogr. A* **2018**, *1581-1582*, 168-172, doi:10.1016/j.chroma.2018.11.008.
129. Loncaric, A.; Matanovic, K.; Ferrer, P.; Kovac, T.; Sarkanj, B.; Skendrovic Babojelic, M.; Lores, M. Peel of Traditional Apple Varieties as a Great Source of Bioactive Compounds: Extraction by Micro-Matrix Solid-Phase Dispersion. *Foods* **2020**, *9*, 4-6, doi:10.3390/foods9010080.
130. Gomez-Mejia, E.; Mikkelsen, L.H.; Rosales-Conrado, N.; Leon-Gonzalez, M.E.; Madrid, Y. A combined approach based on matrix solid-phase dispersion extraction assisted by titanium dioxide nanoparticles and liquid chromatography to determine polyphenols from grape residues. *J. Chromatogr. A* **2021**, *1644*, 462128, doi:10.1016/j.chroma.2021.462128.
131. Martín-Girela, I.; Albero, B.; Tiwari, B.K.; Miguel, E.; Aznar, R. Screening of contaminants of emerging concern in microalgae food supplements. *Separations* **2020**, *7*, doi:10.3390/separations7020028.
132. Arabi, M.; Ostovan, A.; Bagheri, A.R.; Guo, X.; Li, J.; Ma, J.; Chen, L. Hydrophilic molecularly imprinted nanospheres for the extraction of rhodamine B followed by HPLC analysis: A green approach and hazardous waste elimination. *Talanta* **2020**, *215*, 120933-120933, doi:10.1016/j.talanta.2020.120933.
133. Liang, T.; Gao, L.; Qin, D.; Chen, L. Determination of sulfonylurea herbicides in grain samples by matrix solid-phase dispersion with mesoporous structured molecularly imprinted polymer. *Food Analytical Methods* **2019**, *12*, 1938-1948, doi:10.1007/s12161-019-01539-y.
134. Li, P.; Huang, D.; Tang, J.; Zhang, P.; Meng, F. Silica gel impregnated with deep eutectic solvent-based matrix solid-phase dispersion followed by high-performance liquid chromatography for extraction and detection of triazine herbicides in brown sugar. *Anal. Bioanal. Chem.* **2022**, 10.1007/s00216-022-03970-3, 1-9, doi:10.1007/s00216-022-03970-3.
135. Bagheri, A.R.; Arabi, M.; Ghaedi, M.; Ostovan, A.; Wang, X.; Li, J.; Chen, L. Dummy molecularly imprinted polymers based on a green synthesis strategy for magnetic solid-phase extraction of acrylamide in food samples. *Talanta* **2019**, *195*, 390-400, doi:10.1016/j.talanta.2018.11.065.

136. Rashidi Nodeh, H.; Wan Ibrahim, W.A.; Kamboh, M.A.; Sanagi, M.M. Magnetic graphene sol–gel hybrid as clean-up adsorbent for acrylamide analysis in food samples prior to GC–MS. *Food Chem.* **2018**, *239*, 208-216, doi:10.1016/j.foodchem.2017.06.094.
137. Jiang, H.L.; Lin, Y.L.; Li, N.; Wang, Z.W.; Liu, M.; Zhao, R.S.; Lin, J.M. Application of magnetic N-doped carbon nanotubes in solid-phase extraction of trace bisphenols from fruit juices. *Food Chem.* **2018**, *269*, 413-418, doi:10.1016/j.foodchem.2018.07.032.
138. Rahimi, A.; Zanjanchi, M.A.; Bakhtiari, S.; Dehsaraei, M. Selective determination of caffeine in foods with 3D-graphene based ultrasound-assisted magnetic solid phase extraction. *Food Chem.* **2018**, *262*, 206-214, doi:10.1016/j.foodchem.2018.04.035.
139. Özdemir, S.; Mohamedsaid, S.A.; Kılınç, E.; Soylak, M. Magnetic solid phase extractions of Co(II) and Hg(II) by using magnetized C. micaceus from water and food samples. *Food Chem.* **2019**, *271*, 232-238, doi:10.1016/j.foodchem.2018.07.067.
140. Azizi, M.; Seidi, S.; Rouhollahi, A. A novel N,N′-bis(acetylacetone)ethylenediimine functionalized silica-core shell magnetic nanosorbent for manetic dispersive solid phase extraction of copper in cereal and water samples. *Food Chem.* **2018**, *249*, 30-37, doi:10.1016/j.foodchem.2017.12.085.
141. Zhang, W.; Zhou, P.; Liu, W.; Wang, H.; Wang, X. Enhanced adsorption/extraction of five typical polycyclic aromatic hydrocarbons from meat samples using magnetic effervescent tablets composed of dicationic ionic liquids and NiFe2O4 nanoparticles. *J. Mol. Liq.* **2020**, *315*, 113682-113682, doi:10.1016/j.molliq.2020.113682.
142. Zhou, D.B.; Sheng, X.; Han, F.; Hu, Y.Y.; Ding, L.; Lv, Y.L.; Song, W.; Zheng, P. Magnetic solid-phase extraction based on [60]fullerene functionalization of magnetic nanoparticles for the determination of sixteen polycyclic aromatic hydrocarbons in tea samples. *J. Chromatogr. A* **2018**, *1578*, 53-60, doi:10.1016/j.chroma.2018.10.010.
143. Boon, Y.H.; Mohamad Zain, N.N.; Mohamad, S.; Osman, H.; Raoov, M. Magnetic poly(beta-cyclodextrin-ionic liquid) nanocomposites for micro-solid phase extraction of selected polycyclic aromatic hydrocarbons in rice samples prior to GC-FID analysis. *Food Chem.* **2019**, *278*, 322-332, doi:10.1016/j.foodchem.2018.10.145.
144. Gao, Y.; Wang, B.; Yan, Y. Self-Assembling Bifunctional Hydrophilic Magnetic Nanomaterials for Highly Efficient Enrichment of Parabens in Beverages Sample. *ChemistrySelect* **2019**, *4*, 10488-10493, doi:10.1002/slct.201902055.
145. Zhao, J.; Meng, Z.; Zhao, Z.; Zhao, L. Ultrasound-assisted deep eutectic solvent as green and efficient media combined with functionalized magnetic multi-walled carbon nanotubes as solid-phase extraction to determine pesticide residues in food products. *Food Chem.* **2020**, *310*, 125863-125863, doi:10.1016/j.foodchem.2019.125863.
146. Chen, J.Y.; Cao, S.R.; Xi, C.X.; Chen, Y.; Li, X.L.; Zhang, L.; Wang, G.M.; Chen, Y.L.; Chen, Z.Q. A novel magnetic β-cyclodextrin modified graphene oxide adsorbent with high recognition capability for 5 plant growth regulators. *Food Chem.* **2018**, *239*, 911-919, doi:10.1016/j.foodchem.2017.07.013.
147. Narimani-Sabegh, S.; Noroozian, E. Magnetic solid-phase extraction and determination of ultra-trace amounts of antimony in aqueous solutions using maghemite nanoparticles. *Food Chem.* **2019**, *287*, 382-389, doi:10.1016/j.foodchem.2019.02.112.
148. Mesa, R.; Kabir, A.; Samanidou, V.; Furton, K.G. Simultaneous determination of selected estrogenic endocrine disrupting chemicals and bisphenol A residues in whole milk using fabric phase sorptive extraction coupled to HPLC-UV detection and LC-MS/MS. *J. Sep. Sci.* **2019**, *42*, 598-608, doi:10.1002/jssc.201800901.
149. Karageorgou, E.; Manousi, N.; Samanidou, V.; Kabir, A.; Furton, K.G. Fabric phase sorptive extraction for the fast isolation of sulfonamides residues from raw milk followed by high performance liquid chromatography with ultraviolet detection. *Food Chem.* **2016**, *196*, 428-436, doi:10.1016/j.foodchem.2015.09.060.
150. Yang, M.; Gu, Y.; Wu, X.; Xi, X.; Yang, X.; Zhou, W.; Zeng, H.; Zhang, S.; Lu, R.; Gao, H., et al. Rapid analysis of fungicides in tea infusions using ionic liquid immobilized fabric phase sorptive extraction with the assistance of surfactant fungicides analysis using IL-FPSE assisted with surfactant. *Food Chem.* **2018**, *239*, 797-805, doi:10.1016/j.foodchem.2017.06.080.
151. Pérez-Mayán, L.; Rodríguez, I.; Ramil, M.; Kabir, A.; Furton, K.G.; Cela, R. Fabric phase sorptive extraction followed by ultra-performance liquid chromatography-tandem mass spectrometry for the determination of fungicides and insecticides in wine. *J. Chromatogr. A* **2019**, *1584*, 13-23, doi:10.1016/j.chroma.2018.11.025.
152. Ubeda, S.; Aznar, M.; Nerín, C.; Kabir, A. Fabric phase sorptive extraction for specific migration analysis of oligomers from biopolymers. *Talanta* **2021**, *233*, 122603-122603, doi:10.1016/j.talanta.2021.122603.
153. Kaur, R.; Kaur, R.; Rani, S.; Malik, A.K.; Kabir, A.; Furton, K.G. Application of fabric phase sorptive extraction with gas chromatography and mass spectrometry for the determination of organophosphorus pesticides in selected vegetable samples. *J. Sep. Sci.* **2019**, *42*, 862-870, doi:10.1002/jssc.201800854.
154. Gazioglu, I.; Zengin, O.S.; Tartaglia, A.; Locatelli, M.; Furton, K.G.; Kabir, A. Determination of polycyclic aromatic hydrocarbons in nutritional supplements by fabric phase sorptive extraction (FPSE) with high-performance liquid chromatography (HPLC) with fluorescence detection. *Anal. Lett.* **2021**, *54*, 1683-1696, doi:10.1080/00032719.2020.1821209.
155. Guedes-Alonso, R.; Sosa-Ferrera, Z.; Santana-Rodríguez, J.J.; Kabir, A.; Furton, K.G. Fabric phase sorptive extraction of selected steroid hormone residues in commercial raw milk followed by ultra-high-performance liquid chromatography–tandem mass spectrometry. *Foods* **2021**, *10*, doi:10.3390/foods10020343.
156. Agadellis, E.; Tartaglia, A.; Locatelli, M.; Kabir, A.; Furton, K.G.; Samanidou, V. Mixed-mode fabric phase sorptive extraction of multiple tetracycline residues from milk samples prior to high performance liquid chromatography-ultraviolet analysis. *Microchem. J.* **2020**, *159*, 105437-105437, doi:10.1016/j.microc.2020.105437.

157. Manousi, N.; Alampanos, V.; Priovolos, I.; Kabir, A.; Furton, K.G.; Rosenberg, E.; Zachariadis, G.A.; Samanidou, V.F. Exploring sol – gel zwitterionic fabric phase sorptive extraction sorbent as a new multi-mode platform for the extraction and preconcentration of triazine herbicides from juice samples. **2021**.
158. Ghoraba, Z.; Aibaghi, B.; Soleymanpour, A. Ultrasound-assisted dispersive liquid-liquid microextraction followed by ion mobility spectrometry for the simultaneous determination of bendiocarb and azinphos-ethyl in water, soil, food and beverage samples. *Ecotoxicol. Environ. Saf.* **2018**, *165*, 459-466, doi:10.1016/j.ecoenv.2018.09.021.
159. Liu, X.; Chen, M.; Meng, Z.; Qian, H.; Zhang, S.; Lu, R.; Gao, H.; Zhou, W. Extraction of benzoylurea pesticides from tea and fruit juices using deep eutectic solvents. *Journal of Chromatography B: Analytical Technologies in the Biomedical and Life Sciences* **2020**, *1140*, 121995-121995, doi:10.1016/j.jchromb.2020.121995.
160. Ji, Y.; Zhao, M.; Li, A.; Zhao, L. Hydrophobic deep eutectic solvent-based ultrasonic-assisted dispersive liquid-liquid microextraction for preconcentration and determination of trace cadmium and arsenic in wine samples. *Microchem. J.* **2021**, *164*, 105974-105974, doi:10.1016/j.microc.2021.105974.
161. Almeida, J.S.; Meira, L.A.; Nascimento, A.D.S.; Santos, G.L.; Lemos, V.A.; Teixeira, L.S.G. Ultrasound-assisted dispersive liquid-liquid microextraction based on melting of the donor phase: a new approach for the determination of trace elements in solid samples. *Food Analytical Methods* **2021**, *14*, 596-605, doi:10.1007/s12161-020-01897-y.
162. Campone, L.; Celano, R.; Piccinelli, A.L.; Pagano, I.; Cicero, N.; Sanzo, R.D.; Carabetta, S.; Russo, M.; Rastrelli, L. Ultrasound assisted dispersive liquid-liquid microextraction for fast and accurate analysis of chloramphenicol in honey. *Food Res. Int.* **2019**, *115*, 572-579, doi:10.1016/j.foodres.2018.09.006.
163. Tuzen, M.; Elik, A.; Altunay, N. Ultrasound-assisted supramolecular solvent dispersive liquid-liquid microextraction for preconcentration and determination of Cr(VI) in waters and total chromium in beverages and vegetables. *J. Mol. Liq.* **2021**, *329*, 115556, doi:10.1016/j.molliq.2021.115556.
164. Altunay, N.; Elik, A.; Gürkan, R. Monitoring of some trace metals in honeys by flame atomic absorption spectrometry after ultrasound assisted-dispersive liquid liquid microextraction using natural deep eutectic solvent. *Microchem. J.* **2019**, *147*, 49-59, doi:10.1016/j.microc.2019.03.003.
165. Elik, A.; Altunay, N.; Gürkan, R. Ultrasound-Assisted Low-Density Solvent-Based Dispersive Liquid–Liquid Microextraction Coupled to Spectrophotometry for the Determination of Low Levels of Histamine in Fish and Meat Products. *Food Analytical Methods* **2019**, *12*, 489-502, doi:10.1007/s12161-018-1380-1.
166. Salim, S.A.; Sukor, R.; Ismail, M.N.; Selamat, J. Dispersive Liquid-Liquid Microextraction (DLLME) and LC-MS/MS Analysis for Multi-Mycotoxin in Rice Bran: Method Development, Optimization and Validation. *Toxins* **2021**, *13*, 280, doi:10.3390/toxins13040280.
167. Qiao, L.Z.; Sun, R.T.; Yu, C.M.; Tao, Y.; Yan, Y. Novel hydrophobic deep eutectic solvents for ultrasound-assisted dispersive liquid-liquid microextraction of trace non-steroidal anti-inflammatory drugs in water and milk samples. *Microchem. J.* **2021**, *170*, 106686, doi:10.1016/j.microc.2021.106686.
168. Mokhtari, N.; Torbati, M. Synthesis and characterization of phosphocholine chloride-based three- component deep eutectic solvent : application in dispersive liquid – liquid microextraction for determination of organothiophosphate pesticides. **2020**, 10.1002/jsfa.10203, doi:10.1002/jsfa.10203.
169. Altunay, N.; Tuzen, M. A simple and green ultrasound liquid–liquid microextraction method based on low viscous hydrophobic deep eutectic solvent for the preconcentration and separation of selenium in water and food samples prior to HG-AAS detection. *Food Chem.* **2021**, *364*, 130371-130371, doi:10.1016/j.foodchem.2021.130371.
170. Ahmadi-Jouibari, T.; Shaahmadi, Z.; Moradi, M.; Fattahi, N. Extraction and determination of strobilurin fungicides residues in apple samples using ultrasound-assisted dispersive liquid-liquid microextraction based on a novel hydrophobic deep eutectic solvent followed by H.P.L.C-U.V. *Food Additives and Contaminants - Part A Chemistry, Analysis, Control, Exposure and Risk Assessment* **2021**, *00*, 1-11, doi:10.1080/19440049.2021.1978559.
171. Reis, D.; Silva, P.; Perestrelo, R.; Câmara, J.S. Residue Analysis of Insecticides in Potatoes by QuEChERS-dSPE/UHPLC-PDA. *Foods* **2020**, *9*, doi:10.3390/foods9081000.
172. Kamal El-Deen, A.; Shimizu, K. Modified μ-QuEChERS coupled to diethyl carbonate-based liquid microextraction for PAHs determination in coffee, tea, and water prior to GC–MS analysis: An insight to reducing the impact of caffeine on the GC–MS measurement. *Journal of Chromatography B: Analytical Technologies in the Biomedical and Life Sciences* **2021**, *1171*, 122555-122555, doi:10.1016/j.jchromb.2021.122555.
173. Bernardi, G.; Kemmerich, M.; Adaime, M.B.; Prestes, O.D.; Zanella, R. Miniaturized QuEChERS method for determination of 97 pesticide residues in wine by ultra-high performance liquid chromatography coupled with tandem mass spectrometry. *Analytical Methods* **2020**, *12*, 2682-2692, doi:10.1039/d0ay00744g.
174. Casado, N.; Perestrelo, R.; Silva, C.L.; Sierra, I.; Câmara, J.S. An improved and miniaturized analytical strategy based on μ-QuEChERS for isolation of polyphenols. A powerful approach for quality control of baby foods. *Microchem. J.* **2018**, *139*, 110-118, doi:10.1016/j.microc.2018.02.026.
175. Aguiar, J.; Gonçalves, J.L.; Alves, V.L.; Câmara, J.S. Chemical Fingerprint of Free Polyphenols and Antioxidant Activity in Dietary Fruits and Vegetables Using a Non-Targeted Approach Based on QuEChERS Ultrasound-Assisted Extraction Combined with UHPLC-PDA. *Antioxidants (Basel)* **2020**, *9*, doi:10.3390/antiox9040305.

176. Figueira, J.A.; Porto-Figueira, P.; Pereira, J.A.M.; Câmara, J.S. Free low-molecular weight phenolics composition and bioactivity of Vaccinium padifolium Sm fruits. *Food Res. Int.* **2021**, *148*, 110580, doi:10.1016/j.foodres.2021.110580.
177. Izcara, S.; Casado, N.; Morante-Zarcero, S.; Sierra, I. A miniaturized QuEChERS method combined with ultrahigh liquid chromatography coupled to tandem mass spectrometry for the analysis of pyrrolizidine alkaloids in oregano samples. *Foods* **2020**, *9*, doi:10.3390/foods9091319.
178. Izcara, S.; Casado, N.; Morante-Zarcero, S.; Perez-Quintanilla, D.; Sierra, I. Miniaturized and modified QuEChERS method with mesostructured silica as clean-up sorbent for pyrrolizidine alkaloids determination in aromatic herbs. *Food Chem.* **2022**, *380*, 132189, doi:10.1016/j.foodchem.2022.132189.
179. Abreu, D.C.P.; Botrel, B.M.C.; Bazana, M.J.F.; e Rosa, P.V.; Sales, P.F.; Marques, M.d.S.; Saczk, A.A. Development and comparative analysis of single-drop and solid-phase microextraction techniques in the residual determination of 2-phenoxyethanol in fish. *Food Chem.* **2019**, *270*, 487-493, doi:10.1016/j.foodchem.2018.07.136.
180. Saraji, M.; Javadian, S. Single-drop microextraction combined with gas chromatography-electron capture detection for the determination of acrylamide in food samples. *Food Chem.* **2019**, *274*, 55-60, doi:10.1016/j.foodchem.2018.08.108.
181. Jain, A.; Soni, S.; Verma, K.K. Combined liquid phase microextraction and fiber-optics-based cuvetteless micro-spectrophotometry for sensitive determination of ammonia in water and food samples by the indophenol reaction. *Food Chem.* **2021**, *340*, 128156-128156, doi:10.1016/j.foodchem.2020.128156.
182. Neri, T.S.; Rocha, D.P.; Munoz, R.A.A.; Coelho, N.M.M.; Batista, A.D. Highly sensitive procedure for determination of Cu(II) by GF AAS using single-drop microextraction. *Microchem. J.* **2019**, *147*, 894-898, doi:10.1016/j.microc.2019.04.014.
183. Ma, Z.; Zhao, T.; Cui, S.; Zhao, X.; Fan, Y.; Song, J. Determination of ethyl carbamate in wine by matrix modification-assisted headspace single-drop microextraction and gas chromatography – mass spectrometry technique. **2021**.
184. Qi, T.; Xu, M.; Yao, Y.; Chen, W.; Xu, M.; Tang, S.; Shen, W.; Kong, D.; Cai, X.; Shi, H., et al. Gold nanoprism/Tollens' reagent complex as plasmonic sensor in headspace single-drop microextraction for colorimetric detection of formaldehyde in food samples using smartphone readout. *Talanta* **2020**, *220*, 121388, doi:10.1016/j.talanta.2020.121388.
185. Tiwari, S.; Deb, M.K. Modified silver nanoparticles-enhanced single drop microextraction of tartrazine in food samples coupled with diffuse reflectance Fourier transform infrared spectroscopic analysis. *Analytical Methods* **2019**, *11*, 3552-3562, doi:10.1039/c9ay00713j.
186. Shirani, M.; Akbari-adergani, B.; Shahdadi, F.; Faraji, M.; Akbari, A. A Hydrophobic Deep Eutectic Solvent-Based Ultrasound-Assisted Dispersive Liquid–Liquid Microextraction for Determination of β-Lactam Antibiotics Residues in Food Samples. *Food Analytical Methods* **2021**, *15*, 391-400, doi:10.1007/s12161-021-02122-0.
187. Nemati, M.; Mogaddam, M.R.A.; Farazajdeh, M.A.; Tuzen, M.; Khandaghi, J. In-situ formation/decomposition of deep eutectic solvent during solidification of floating organic droplet-liquid-liquid microextraction method for the extraction of some antibiotics from honey prior to high performance liquid chromatography-tandem mass s. *J. Chromatogr. A* **2021**, 10.1016/j.chroma.2021.462653, 462653, doi:10.1016/j.chroma.2021.462653.
188. Nemati, M.; Farajzadeh, M.A.; Mohebbi, A.; Khodadadeian, F.; Afshar Mogaddam, M.R. Development of a stir bar sorptive extraction method coupled to solidification of floating droplets dispersive liquid–liquid microextraction based on deep eutectic solvents for the extraction of acidic pesticides from tomato samples. *J. Sep. Sci.* **2020**, *43*, 1119-1127, doi:10.1002/jssc.201901000.
189. Urucu, O.A.; Yetimoglu, E.K.; Donmez, S.; Deniz, S. Undecanol-ethanol-water ternary system-based microextraction for the detection of cadmium. *J. Serb. Chem. Soc.* **2019**, *84*, 435-443, doi:10.2298/Jsc180831110u.
190. Trujillo-Rodríguez, M.J.; Pino, V.; Anderson, J.L. Magnetic ionic liquids as extraction solvents in vacuum headspace single-drop microextraction. *Talanta* **2017**, *172*, 86-94, doi:10.1016/j.talanta.2017.05.021.
191. Chen, S.; Yan, J.; Liu, Y.; Wang, C.; Lu, D. Determination of Mn(II) and Mn(VII) in beverage samples using magnetic dispersive micro-solid phase extraction coupled with solidified floating organic drop microextraction followed by graphite furnace atomic absorption spectrometry. *Food Chem.* **2021**, *359*, 129958-129958, doi:10.1016/j.foodchem.2021.129958.
192. Tavakoli, M.; Jamali, M.R.; Nezhadali, A. Ultrasound-Assisted Dispersive Liquid–Liquid Microextraction (DLLME) Based on Solidification of Floating Organic Drop Using a Deep Eutectic Solvent for Simultaneous Preconcentration and Determination of Nickel and Cobalt in Food and Water Samples. *Anal. Lett.* **2021**, *54*, 2863-2873, doi:10.1080/00032719.2021.1897990.
193. Mardani, A.; Torbati, M.; Farajzadeh, M.A.; Mohebbi, A.; Alizadeh, A.A.; Afshar Mogaddam, M.R. Development of temperature-assisted solidification of floating organic droplet-based dispersive liquid–liquid microextraction performed during centrifugation for extraction of organochlorine pesticide residues in cocoa powder prior to GC-ECD. *Chemical Papers* **2021**, *75*, 1691-1700, doi:10.1007/s11696-020-01424-7.
194. Mao, X.; Wan, Y.; Li, Z.; Chen, L.; Lew, H.; Yang, H. Analysis of organophosphorus and pyrethroid pesticides in organic and conventional vegetables using QuEChERS combined with dispersive liquid-liquid microextraction based on the solidification of floating organic droplet. *Food Chem.* **2020**, *309*, 125755, doi:10.1016/j.foodchem.2019.125755.
195. Fazaieli, F.; Afshar Mogaddam, M.R.; Farajzadeh, M.A.; Feriduni, B.; Mohebbi, A. Development of organic solvents-free mode of solidification of floating organic droplet–based dispersive liquid–liquid microextraction for the extraction of polycyclic aromatic hydrocarbons from honey samples before their determination by gas chromatograp. *J. Sep. Sci.* **2020**, *43*, 2393-2400, doi:10.1002/jssc.202000136.

196. Abolghasemi, M.M.; Piryaei, M.; Imani, R.M. Deep eutectic solvents as extraction phase in head-space single-drop microextraction for determination of pesticides in fruit juice and vegetable samples. *Microchem. J.* **2020**, *158*, 105041-105041, doi:10.1016/j.microc.2020.105041.
197. Afshar Mogaddam, M.R.; Farajzadeh, M.A.; Azadmard Damirchi, S.; Nemati, M. Dispersive solid phase extraction combined with solidification of floating organic drop–liquid–liquid microextraction using in situ formation of deep eutectic solvent for extraction of phytosterols from edible oil samples. *J. Chromatogr. A* **2020**, *1630*, 461523, doi:10.1016/j.chroma.2020.461523.
198. Triaux, Z.; Petitjean, H.; Marchioni, E.; Boltoeva, M.; Marcic, C. Deep eutectic solvent–based headspace single-drop microextraction for the quantification of terpenes in spices. *Anal. Bioanal. Chem.* **2020**, *412*, 933-948, doi:10.1007/s00216-019-02317-9.
199. Piryaei, M.; Behroozi, M. Deep Eutectic Solvent as a Green Solvent for Fast Analysis of the Volatile Components of Satureja Hortensis L. *Phys. Chem. Res* **2022**, *10*, 421-428, doi:10.22036/PCR.2022.312969.1979.
200. Barbosa-Pereira, L.; Guglielmetti, A.; Zeppa, G. Pulsed Electric Field Assisted Extraction of Bioactive Compounds from Cocoa Bean Shell and Coffee Silverskin. *Food and Bioprocess Technology* **2018**, *11*, 818-835, doi:10.1007/s11947-017-2045-6.
201. Redondo, D.; Venturini, M.E.; Luengo, E.; Raso, J.; Arias, E. Pulsed electric fields as a green technology for the extraction of bioactive compounds from thinned peach by-products. *Innov. Food Sci. Emerg. Technol.* **2018**, *45*, 335-343, doi:10.1016/j.ifset.2017.12.004.
202. Pataro, G.; Carullo, D.; Ferrari, G. Effect of PEF pre-treatment and extraction temperature on the recovery of carotenoids from tomato wastes. *Chemical Engineering Transactions* **2019**, *75*, 139-144, doi:10.3303/CET1975024.
203. Mahalleh, A.A.; Sharayei, P.; Mortazavi, S.A.; Azarpazhooh, E.; Niazmand, R. Optimization of the pulsed electric field-assisted extraction of functional compounds from nepeta binaludensis. *Agricultural Engineering International: CIGR Journal* **2019**, *21*, 184-194.
204. Moghaddam, T.N.; Elhamirad, A.H.; Asl, M.R.S.; Noghabi, M.S. Pulsed electric field-assisted extraction of phenolic antioxidants from tropical almond red leaves. *Chemical Papers* **2020**, *74*, 3957-3961, doi:10.1007/s11696-020-01153-x.
205. Tzima, K.; Brunton, N.P.; Lyng, J.G.; Frontuto, D.; Rai, D.K. The effect of Pulsed Electric Field as a pre-treatment step in Ultrasound Assisted Extraction of phenolic compounds from fresh rosemary and thyme by-products. *Innov. Food Sci. Emerg. Technol.* **2021**, *69*, 102644-102644, doi:10.1016/j.ifset.2021.102644.
206. Dong, Z.Y.; Wang, H.H.; Li, M.Y.; Liu, W.; Zhang, T.H. Optimization of high-intensity pulsed electric field-assisted extraction of procyanidins from Vitis amurensis seeds using response surface methodology. *E3S Web of Conferences* **2020**, *189*, doi:10.1051/e3sconf/202018902029.
207. Mahn, A.; Comett, R.; Segura-Ponce, L.A.; Díaz-Álvarez, R.E. Effect of pulsed electric field-assisted extraction on recovery of sulforaphane from broccoli florets. *J. Food Process Eng.* **2021**, 10.1111/jfpe.13837, 3-8, doi:10.1111/jfpe.13837.
208. Santos, P.H.; Kammers, J.C.; Silva, A.P.; Oliveira, J.V.; Hense, H. Antioxidant and antibacterial compounds from feijoa leaf extracts obtained by pressurized liquid extraction and supercritical fluid extraction. *Food Chem.* **2021**, *344*, 128620-128620, doi:10.1016/j.foodchem.2020.128620.
209. Casas-Cardoso, L.; Mantell, C.; Obregon, S.; Cejudo-Bastante, C.; Alonso-Moraga, A.; de la Ossa, E.J.M.; de Haro-Bailon, A. Health-Promoting Properties of Borage Seed Oil Fractionated by Supercritical Carbon Dioxide Extraction. *Foods* **2021**, *10*, doi:10.3390/foods10102471.
210. Santos, O.V.; Lorenzo, N.D.; Souza, A.L.G.; Costa, C.E.F.; Conceição, L.R.V.; Lannes, S.C.d.S.; Teixeira-Costa, B.E. CO2 supercritical fluid extraction of pulp and nut oils from Terminalia catappa fruits: Thermogravimetric behavior, spectroscopic and fatty acid profiles. *Food Res. Int.* **2021**, *139*, doi:10.1016/j.foodres.2020.109814.
211. Deka, D.; Hulle, N.R.S. Supercritical fluid extraction of Bhut Jolokia oleoresin and its quality analysis. *Sn Applied Sciences* **2021**, *3*, doi:ARTN 260, 10.1007/s42452-021-04218-y.
212. Mihalcea, L.; Turturica, M.; Cucolea, E.I.; Danila, G.M.; Dumitrascu, L.; Coman, G.; Constantin, O.E.; Grigore-Gurgu, L.; Stanciuc, N. CO2 Supercritical Fluid Extraction of Oleoresins from Sea Buckthorn Pomace: Evidence of Advanced Bioactive Profile and Selected Functionality. *Antioxidants (Basel)* **2021**, *10*, doi:10.3390/antiox10111681.
213. Jha, A.K.; Sit, N. Comparison of response surface methodology (RSM) and artificial neural network (ANN) modelling for supercritical fluid extraction of phytochemicals from Terminalia chebula pulp and optimization using RSM coupled with desirability function (DF) and genetic. *Ind. Crops Prod.* **2021**, *170*, doi:10.1016/j.indcrop.2021.113769.
214. Arturo-Perdomo, D.; Mora, J.P.J.; Ibáñez, E.; Cifuentes, A.; Hurtado-Benavides, A.; Montero, L. Extraction and Characterization of the Polar Lipid Fraction of Blackberry and Passion Fruit Seeds Oils Using Supercritical Fluid Extraction. *Food Analytical Methods* **2021**, *14*, 2026-2037, doi:10.1007/s12161-021-02020-5.
215. Cuco, R.P.; Cardozo-Filho, L.; Silva, C.d. Simultaneous extraction of seed oil and active compounds from peel of pumpkin (Cucurbita maxima) using pressurized carbon dioxide as solvent. *J. Supercrit. Fluids* **2019**, *143*, 8-15, doi:10.1016/j.supflu.2018.08.002.
216. Rodrigues, L.A.; Matias, A.A.; Paiva, A. Recovery of antioxidant protein hydrolysates from shellfish waste streams using subcritical water extraction. *Food Bioprod. Process.* **2021**, *130*, 154-163, doi:10.1016/j.fbp.2021.09.011.
217. Wang, Y.; Ye, Y.; Wang, L.; Yin, W.; Liang, J. Antioxidant activity and subcritical water extraction of anthocyanin from raspberry process optimization by response surface methodology. *Food Bioscience* **2021**, *44*, 101394-101394, doi:10.1016/j.fbio.2021.101394.

218. Salami, A.; Asefi, N.; Kenari, R.E.; Gharekhani, M. Extraction of pumpkin peel extract using supercritical CO2 and subcritical water technology: Enhancing oxidative stability of canola oil. *Journal of Food Science and Technology* **2021**, *58*, 1101-1109, doi:10.1007/s13197-020-04624-x.
219. Svarc-Gajic, J.; Cerda, V.; Delerue-Matos, C.; Maskovic, P.; Clavijo, S.; Suarez, R.; Cvetanovic, A.; Ramalhosa, M.J.; Barroso, M.F.; Moreira, M., et al. Chemical Characterization and In Vitro Bioactivity of Apple Bark Extracts Obtained by Subcritical Water. *Waste and Biomass Valorization* **2021**, *12*, 6781-6794, doi:10.1007/s12649-021-01477-z.
220. Pangestuti, R.; Haq, M.; Rahmadi, P.; Chun, B.-s. Nutritional Value and Biofunctionalities of Two Edible Green Seaweeds (Ulva lactuca and Caulerpa racemosa) from Indonesia by Subcritical Water Hydrolysis. **2021**.
221. Hwang, H.J.; Kim, H.J.; Ko, M.J.; Chung, M.S. Recovery of hesperidin and narirutin from waste Citrus unshiu peel using subcritical water extraction aided by pulsed electric field treatment. *Food Science and Biotechnology* **2021**, *30*, 217-226, doi:10.1007/s10068-020-00862-z.
222. Zhang, F.; Zhang, L.; Chen, J.; Du, X.; Lu, Z.; Wang, X.; Yi, Y.; Shan, Y.; Liu, B.; Zhou, Y., et al. Systematic evaluation of a series of pectic polysaccharides extracted from apple pomace by regulation of subcritical water conditions. *Food Chem.* **2022**, *368*, 130833-130833, doi:10.1016/j.foodchem.2021.130833.
223. Pinto, D.; Vieira, E.F.; Peixoto, A.F.; Freire, C.; Freitas, V.; Costa, P.; Delerue-Matos, C.; Rodrigues, F. Optimizing the extraction of phenolic antioxidants from chestnut shells by subcritical water extraction using response surface methodology. *Food Chem.* **2021**, *334*, 127521-127521, doi:10.1016/j.foodchem.2020.127521.
224. Mazyan, W.I.; O'connor, E.; Martin, E.; Vogt, A.; Charter, E.; Ahmadi, A. Effects of temperature and extraction time on avocado flesh (Persea americana) total phenolic yields using subcritical water extraction. *Processes* **2021**, *9*, 1-13, doi:10.3390/pr9010159.
225. Machmudah, S.; Wahyu Fitriana, M.; Fatbamayani, N.; Wahyudiono; Kanda, H.; Winardi, S.; Goto, M. Phytochemical compounds extraction from medicinal plants by subcritical water and its encapsulation via electrospraying. *Alexandria Engineering Journal* **2021**, 10.1016/j.aej.2021.07.033, doi:10.1016/j.aej.2021.07.033.
226. Jamaludin, R.; Kim, D.S.; Salleh, L.M.; Lim, S.B. Kinetic study of subcritical water extraction of scopoletin, alizarin, and rutin from morinda citrifolia. *Foods* **2021**, *10*, 1-13, doi:10.3390/foods10102260.
227. Vera, J.; Fernandes, V.C.; Correia-Sá, L.; Mansilha, C.; Delerue-Matos, C.; Domingues, V.F. Occurrence of Selected Known or Suspected Endocrine-Disrupting Pesticides in Portuguese Surface Waters Using SPME-GC-IT/MS. *Separations* **2021**, *8*, doi:10.3390/separations8060081.
228. Sun, M.; Feng, J.; Bu, Y.; Luo, C. Ionic liquid coated copper wires and tubes for fiber-in-tube solid-phase microextraction. *J. Chromatogr. A* **2016**, *1458*, 1-8, doi:10.1016/j.chroma.2016.06.023.
229. Saliu, F.; Montano, S.; Hoeksema, B.W.; Lasagni, M.; Galli, P. A non-lethal SPME-LC/MS method for the analysis of plastic-associated contaminants in coral reef invertebrates. *Analytical Methods* **2020**, *12*, 1935-1942, doi:10.1039/C9AY02621E.
230. Wang, R.; Li, W.; Chen, Z. Solid phase microextraction with poly(deep eutectic solvent) monolithic column online coupled to HPLC for determination of non-steroidal anti-inflammatory drugs. *Anal. Chim. Acta* **2018**, *1018*, 111-118, doi:10.1016/j.aca.2018.02.024.
231. Aladaghlo, Z.; Maddah, B.; Fakhari, A.R. Fabrication of Co3O4 quantum dot incorporated polyacrylamide ethylene glycol dimethacrylate as a new fiber for solid phase microextraction and trace determination of organophosphorus pesticides in environmental water samples. *Analytical Methods* **2021**, *13*, 3394-3401, doi:10.1039/D1AY00855B.
232. Ghiasvand, A.; Yazdankhah, F.; Paull, B. Heating-, Cooling- and Vacuum-Assisted Solid-Phase Microextraction (HCV-SPME) for Efficient Sampling of Environmental Pollutants in Complex Matrices. *Chromatographia* **2020**, *83*, 531-540, doi:10.1007/s10337-020-03869-0.
233. Beiranvand, M.; Ghiasvand, A. Design and optimization of the VA-TV-SPME method for ultrasensitive determination of the PAHs in polluted water. *Talanta* **2020**, *212*, 120809, doi:10.1016/j.talanta.2020.120809.
234. Zang, X.; Pang, Y.; Li, H.; Chang, Q.; Zhang, S.; Wang, C.; Wang, Z. Solid phase microextraction of polycyclic aromatic hydrocarbons from water samples by a fiber coated with covalent organic framework modified graphitic carbon nitride. *J. Chromatogr. A* **2020**, *1628*, 461428, doi:10.1016/j.chroma.2020.461428.
235. Terzaghi, E.; Falakdin, P.; Fattore, E.; Di Guardo, A. Estimating temporal and spatial levels of PAHs in air using rain samples and SPME analysis: Feasibility evaluation in an urban scenario. *Sci. Total Environ.* **2021**, *762*, 144184, doi:10.1016/j.scitotenv.2020.144184.
236. Merdivan, M.; Pino, V.; Anderson, J.L. Determination of volatile polycyclic aromatic hydrocarbons in waters using headspace solid-phase microextraction with a benzyl-functionalized crosslinked polymeric ionic liquid coating. *Environ. Technol.* **2017**, *38*, 1897-1904, doi:10.1080/09593330.2016.1240242.
237. Hosseinzadegan, S.; Nischkauer, W.; Bica, K.; Limbeck, A. FI-ICP-OES determination of Pb in drinking water after pre-concentration using magnetic nanoparticles coated with ionic liquid. *Microchem. J.* **2019**, *146*, 339-344, doi:10.1016/j.microc.2019.01.029.
238. Mirzajani, R.; Kardani, F.; Ramezani, Z. Fabrication of UMCM-1 based monolithic and hollow fiber – Metal-organic framework deep eutectic solvents/molecularly imprinted polymers and their use in solid phase microextraction of phthalate esters in yogurt, water and edible oil by GC-FID. *Food Chem.* **2020**, *314*, 126179-126179, doi:10.1016/j.foodchem.2020.126179.

239. Valenzuela, E.F.; de Paula, F.G.F.; Teixeira, A.P.C.; Menezes, H.C.; Cardeal, Z.L. A new carbon nanomaterial solid-phase microextraction to pre-concentrate and extract pesticides in environmental water. *Talanta* **2020**, *217*, 121011, doi:10.1016/j.talanta.2020.121011.
240. Li, T.; Song, Y.; Xu, J.; Fan, J. A hydrophobic deep eutectic solvent mediated sol-gel coating of solid phase microextraction fiber for determination of toluene, ethylbenzene and o-xylene in water coupled with GC-FID. *Talanta* **2019**, *195*, 298-305, doi:10.1016/j.talanta.2018.11.085.
241. Alvarez-Martin, A.; McHugh, K.; Martin, C.; Kavich, G.; Kaczkowski, R. Understanding air-tight case environments at the National Museum of the American Indian (Smithsonian Institution) by SPME-GC-MS analysis. *Journal of Cultural Heritage* **2020**, *44*, 38-46, doi:10.1016/j.culher.2020.01.004.
242. Moufid, M.; Hofmann, M.; El Bari, N.; Tiebe, C.; Bartholmai, M.; Bouchikhi, B. Wastewater monitoring by means of e-nose, VE-tongue, TD-GC-MS, and SPME-GC-MS. *Talanta* **2021**, *221*, 121450, doi:10.1016/j.talanta.2020.121450.
243. Trujillo-Rodríguez, M.J.; Nan, H.; Anderson, J.L. Expanding the use of polymeric ionic liquids in headspace solid-phase microextraction: Determination of ultraviolet filters in water samples. *J. Chromatogr. A* **2018**, *1540*, 11-20, doi:10.1016/j.chroma.2018.01.048.
244. Shirkhanloo, H.; Khaleghi Abbasabadi, M.; Hosseini, F.; Faghihi Zarandi, A. Nanographene oxide modified phenyl methanethiol nanomagnetic composite for rapid separation of aluminum in wastewaters, foods, and vegetable samples by microwave dispersive magnetic micro solid-phase extraction. *Food Chem.* **2021**, *347*, 129042, doi:10.1016/j.foodchem.2021.129042.
245. Althoff, M.A.; Bertsch, A.; Metzulat, M. Automation of μ-SPE (Smart-SPE) and Liquid-Liquid Extraction Applied for the Analysis of Chemical Warfare Agents. *Separations* **2019**, *6*, 49.
246. Zhang, Y.; Li, Q.; Gao, Q.; Li, J.; Shen, Y.; Zhu, X. An aspirated in-syringe device fixed with ionic liquid and β-cyclodextrin-functionalized CNTs/TiO 2 for rapid adsorption and visible-light-induced photocatalytic activity. *New J. Chem.* **2019**, *43*, 9345-9353, doi:10.1039/C9NJ01602C.
247. Rozaini, M.N.H.; Kiatkittipong, W.; Saad, B.; Yahaya, N.; Shaharun, M.S.; Sangu, S.S.; Mohamed Saheed, M.S.; Wong, Y.F.; Mohamad, M.; Sambudi, N.S., et al. Green adsorption–desorption of mixed triclosan, triclocarban, 2-phenylphenol, bisphenol A and 4-tert-octylphenol using MXene encapsulated polypropylene membrane protected micro-solid-phase extraction device in amplifying the HPLC analysis. *Microchem. J.* **2021**, *170*, 106695, doi:10.1016/j.microc.2021.106695.
248. Naing, N.N.; Goh, E.X.Y.; Lee, H.K. Enhanced microextraction of endocrine disrupting chemicals adsorbed on airborne fine particulate matter with gas chromatography-tandem mass spectrometric analysis. *J. Chromatogr. A* **2021**, *1637*, 461828, doi:10.1016/j.chroma.2020.461828.
249. Tan, S.C.; Zulkifli, F.I.b.; Lee, H.K. Solvent-loaded metal-organic framework of type MIL-101(Cr)-NH2 for the dispersive solid-phase extraction and UHPLC-MS/MS analysis of herbicides from paddy field waters. *Microchimica Acta* **2021**, *188*, doi:10.1007/S00604-020-04661-5.
250. Di, X.; Zhao, X.; Guo, X. Dispersive micro-solid phase extraction combined with switchable hydrophilicity solvent-based homogeneous liquid-liquid microextraction for enrichment of non-steroidal anti-inflammatory drugs in environmental water samples. *J. Chromatogr. A* **2020**, *1634*, 461677, doi:10.1016/j.chroma.2020.461677.
251. Abdi Hassan, A.; Sajid, M.; Al Ghafly, H.; Alhooshani, K. Ionic liquid-based membrane-protected micro-solid-phase extraction of organochlorine pesticides in environmental water samples. *Microchem. J.* **2020**, *158*, doi:10.1016/J.MICROC.2020.105295.
252. Abaroa-Pérez, B.; Sánchez-Almeida, G.; Hernández-Brito, J.J.; Vega-Moreno, D. In Situ Miniaturised Solid Phase Extraction (m-SPE) for Organic Pollutants in Seawater Samples. *Journal of Analytical Methods in Chemistry* **2018**, *2018*, 7437031, doi:10.1155/2018/7437031.
253. Tan, S.C.; Sin Leow, J.W.; Lee, H.K. Emulsification-assisted micro-solid-phase extraction using a metal-organic framework as sorbent for the liquid chromatography-tandem mass spectrometric analysis of polar herbicides from aqueous samples. *Talanta* **2020**, *216*, 120962, doi:10.1016/j.talanta.2020.120962.
254. Darvishnejad, M.; Ebrahimzadeh, H. Graphitic carbon nitride-reinforced polymer ionic liquid nanocomposite: a novel mixed-mode sorbent for microextraction in packed syringe. *Int. J. Environ. Anal. Chem.* **2020**, 10.1080/03067319.2020.1770243, 1-14, doi:10.1080/03067319.2020.1770243.
255. Saraji, M.; Jafari, M.T.; Amooshahi, M.M. Sol-gel/nanoclay composite as a sorbent for microextraction in packed syringe combined with corona discharge ionization ion mobility spectrometry for the determination of diazinon in water samples. *J. Sep. Sci.* **2018**, *41*, 493-500, doi:10.1002/jssc.201700967.
256. Matin, P.; Ayazi, Z.; Jamshidi-Ghaleh, K. Montmorillonite reinforced polystyrene nanocomposite supported on cellulose as a novel layered sorbent for microextraction by packed sorbent for determination of fluoxetine followed by spectrofluorimetry based on multivariate optimisation. *Int. J. Environ. Anal. Chem.* **2020**, 10.1080/03067319.2020.1791333, 1-16, doi:10.1080/03067319.2020.1791333.
257. Moradi, E.; Mehrani, Z.; Ebrahimzadeh, H. Gelatin/sodium triphosphate hydrogel electrospun nanofiber mat as a novel nanosorbent for microextraction in packed syringe of La3+ and Tb3+ ions prior to their determination by ICP-OES. *Reactive & Functional Polymers* **2020**, *153*, 104627, doi:10.1016/j.reactfunctpolym.2020.104627.

258. Serenjeh, F.N.; Hashemi, P.; Ghiasvand, A.R.; Rasolzadeh, F.; Heydari, N.; Badiei, A. Cooling assisted headspace microextraction by packed sorbent coupled to HPLC for the determination of volatile polycyclic aromatic hydrocarbons in soil. *Anal. Chim. Acta* **2020**, *1125*, 128-134, doi:10.1016/j.aca.2020.05.067.
259. Mehrani, Z.; Ebrahimzadeh, H.; Moradi, E. Use of aloin-based and rosin-based electrospun nanofibers as natural nanosorbents for the extraction of polycyclic aromatic hydrocarbons and phenoxyacetic acid herbicides by microextraction in packed syringe method prior to GC-FID detection. *Mikrochim. Acta* **2020**, *187*, 401, doi:10.1007/s00604-020-04374-9.
260. Arcoleo, A.; Bianchi, F.; Careri, M. A sensitive microextraction by packed sorbent-gas chromatography-mass spectrometry method for the assessment of polycyclic aromatic hydrocarbons contamination in Antarctic surface snow. *Chemosphere* **2021**, *282*, 131082, doi:10.1016/j.chemosphere.2021.131082.
261. Taghani, A.; Goudarzi, N.; Bagherian, G.A.; Arab Chamjangali, M.; Amin, A.H. Application of nanoperlite as a new natural sorbent in the preconcentration of three organophosphorus pesticides by microextraction in packed syringe coupled with gas chromatography and mass spectrometry. *J. Sep. Sci.* **2018**, *41*, 2245-2252, doi:10.1002/jssc.201701276.
262. Amiri, A.; Chahkandi, M.; Targhoo, A. Synthesis of nano-hydroxyapatite sorbent for microextraction in packed syringe of phthalate esters in water samples. *Anal. Chim. Acta* **2017**, *950*, 64-70, doi:10.1016/j.aca.2016.11.027.
263. Kaykhaii, M.; Hashemi, S.H.; Andarz, F.; Piri, A.; Sargazi, G. Chromium-based metal organic framework for pipette tip micro-solid phase extraction: an effective approach for determination of methyl and propyl parabens in wastewater and shampoo samples. *BMC Chemistry* **2021**, *15*, 60, doi:10.1186/s13065-021-00786-7.
264. Liu, L.; Tang, W.; Tang, B.; Han, D.; Row, K.H.; Zhu, T. Pipette-tip solid-phase extraction based on deep eutectic solvent modified graphene for the determination of sulfamerazine in river water. *J. Sep. Sci.* **2017**, *40*, 1887-1895, doi:10.1002/jssc.201601436.
265. Wu, B.; Muhammad, T.; Aihebaier, S.; Karim, K.; Hu, Y.; Piletsky, S. A molecularly imprinted polymer based monolith pipette tip for solid-phase extraction of 2,4-dichlorophenoxyacetic acid in an aqueous sample. *Analytical Methods* **2020**, *12*, 4913-4921, doi:10.1039/D0AY01587C.
266. Mohammadi, F.; Esrafili, A.; Kermani, M.; Farzadkia, M.; Gholami, M.; Behbahani, M. Application of amino modified mesostructured cellular foam as an efficient mesoporous sorbent for dispersive solid-phase extraction of atrazine from environmental water samples. *Microchem. J.* **2019**, *146*, 753-762, doi:10.1016/j.microc.2019.01.049.
267. Zhang, X.; Sun, X.; Wang, M.; Wang, Y.; Wu, Q.; Ji, L.; Li, Q.; Yang, J.; Zhou, Q. Dummy molecularly imprinted microspheres prepared by Pickering emulsion polymerization for matrix solid-phase dispersion extraction of three azole fungicides from fish samples. *J. Chromatogr. A* **2020**, *1620*, 461013, doi:10.1016/j.chroma.2020.461013.
268. Aznar, R.; Albero, B.; Sánchez-Brunete, C.; Miguel, E.; Martín-Girela, I.; Tadeo, J.L. Simultaneous determination of multiclass emerging contaminants in aquatic plants by ultrasound-assisted matrix solid-phase dispersion and GC-MS. *Environ Sci Pollut Res Int* **2017**, *24*, 7911-7920, doi:10.1007/s11356-016-6327-8.
269. Yu, H.; Wang, Z.; Wu, R.; Chen, X.; Chan, T.D. Water-dispersible pH/thermo dual-responsive microporous polymeric microspheres as adsorbent for dispersive solid-phase extraction of fluoroquinolones from environmental water samples and food samples. *J. Chromatogr. A* **2019**, *1601*, 27-34, doi:10.1016/j.chroma.2019.05.004.
270. Castro, V.; Montes, R.; Quintana, J.B.; Rodil, R.; Cela, R. Determination of 18 organophosphorus flame retardants/plasticizers in mussel samples by matrix solid-phase dispersion combined to liquid chromatography-tandem mass spectrometry. *Talanta* **2020**, *208*, 120470, doi:10.1016/j.talanta.2019.120470.
271. Xu, S.; Li, H.; Wu, H.; Xiao, L.; Dong, P.; Feng, S.; Fan, J. A facile cooling-assisted solid-phase microextraction device for solvent-free sampling of polycyclic aromatic hydrocarbons from soil based on matrix solid-phase dispersion technique. *Anal. Chim. Acta* **2020**, *1115*, 7-15, doi:10.1016/j.aca.2020.04.019.
272. Soares, K.L.; Cerqueira, M.B.R.; Caldas, S.S.; Primel, E.G. Evaluation of alternative environmentally friendly matrix solid phase dispersion solid supports for the simultaneous extraction of 15 pesticides of different chemical classes from drinking water treatment sludge. *Chemosphere* **2017**, *182*, 547-554, doi:10.1016/j.chemosphere.2017.05.062.
273. Balsebre, A.; Báez, M.E.; Martínez, J.; Fuentes, E. Matrix solid-phase dispersion associated to gas chromatography for the assessment in honey bee of a group of pesticides of concern in the apicultural field. *J. Chromatogr. A* **2018**, *1567*, 47-54, doi:10.1016/j.chroma.2018.06.062.
274. Rodriguez-Gonzalez, N.; Gonzalez-Castro, M.J.; Beceiro-Gonzalez, E.; Muniategui-Lorenzo, S. Development of a matrix solid phase dispersion methodology for the determination of triazine herbicides in marine sediments. *Microchem. J.* **2017**, *133*, 137-143, doi:10.1016/j.microc.2017.03.022.
275. Zhang, J.; Yu, F.; Tao, Y.; Du, C.; Yang, W.; Chen, W.; Tu, X. Micro Salting-Out Assisted Matrix Solid-Phase Dispersion: A Simple and Fast Sample Preparation Method for the Analysis of Bisphenol Contaminants in Bee Pollen. *Molecules* **2021**, *26*, doi:10.3390/molecules26082350.
276. Ferreira, T.; Lima, I.; Magalhaes, V.; Avelar, B.; Oliveira, G.; Barbour Scott, F.; Cid, Y. Bioanalytical Method to Measure Fluazuron in Bovine Plasma and its Application in Pharmacokinetic Studies. *Revista Virtual de Química* **2019**, *11*, 1067-1079, doi:10.21577/1984-6835.20190072.
277. Fan, J.; Liu, Z.; Li, J.; Zhou, W.; Gao, H.; Zhang, S.; Lu, R. PEG-modified magnetic Schiff base network-1 materials for the magnetic solid phase extraction of benzoylurea pesticides from environmental water samples. *J. Chromatogr. A* **2020**, *1619*, 460950, doi:10.1016/j.chroma.2020.460950.

278. Kobylinska, N.; Kostenko, L.; Khainakov, S.; Garcia-Granda, S. Advanced core-shell EDTA-functionalized magnetite nanoparticles for rapid and efficient magnetic solid phase extraction of heavy metals from water samples prior to the multi-element determination by ICP-OES. *Microchimica Acta* **2020**, *187*, 289, doi:10.1007/s00604-020-04231-9.
279. Ma, J.; Wu, G.; Li, S.; Tan, W.; Wang, X.; Li, J.; Chen, L. Magnetic solid-phase extraction of heterocyclic pesticides in environmental water samples using metal-organic frameworks coupled to high performance liquid chromatography determination. *J. Chromatogr. A* **2018**, *1553*, 57-66, doi:10.1016/j.chroma.2018.04.034.
280. Liu, X.; Gao, S.; Li, X.; Wang, H.; Ji, X.; Zhang, Z. Determination of microcystins in environmental water samples with ionic liquid magnetic graphene. *Ecotoxicol. Environ. Saf.* **2019**, *176*, 20-26, doi:10.1016/j.ecoenv.2019.03.063.
281. Shakourian, M.; Yamini, Y.; Safari, M. Facile magnetization of metal-organic framework TMU-6 for magnetic solid-phase extraction of organophosphorus pesticides in water and rice samples. *Talanta* **2020**, *218*, 121139, doi:10.1016/j.talanta.2020.121139.
282. Shahriman, M.S.; Ramachandran, M.R.; Zain, N.N.M.; Mohamad, S.; Manan, N.S.A.; Yaman, S.M. Polyaniline-dicationic ionic liquid coated with magnetic nanoparticles composite for magnetic solid phase extraction of polycyclic aromatic hydrocarbons in environmental samples. *Talanta* **2018**, *178*, 211-221, doi:10.1016/j.talanta.2017.09.023.
283. Abujaber, F.; Zougagh, M.; Jodeh, S.; Ríos, Á.; Guzmán Bernardo, F.J.; Rodríguez Martín-Doimeadios, R.C. Magnetic cellulose nanoparticles coated with ionic liquid as a new material for the simple and fast monitoring of emerging pollutants in waters by magnetic solid phase extraction. *Microchem. J.* **2018**, *137*, 490-495, doi:10.1016/j.microc.2017.12.007.
284. Manousi, N.; Deliyanni, E.A.; Rosenberg, E.; Zachariadis, G.A. Ultrasound-assisted magnetic solid-phase extraction of polycyclic aromatic hydrocarbons and nitrated polycyclic aromatic hydrocarbons from water samples with a magnetic polyaniline modified graphene oxide nanocomposite. *J. Chromatogr. A* **2021**, *1645*, 462104, doi:10.1016/j.chroma.2021.462104.
285. Zhou, Q.; Lei, M.; Wu, Y.; Zhou, X.; Wang, H.; Sun, Y.; Sheng, X.; Tong, Y. Magnetic solid phase extraction of bisphenol A, phenol and hydroquinone from water samples by magnetic and thermo dual-responsive core-shell nanomaterial. *Chemosphere* **2020**, *238*, 124621, doi:10.1016/j.chemosphere.2019.124621.
286. He, M.; Su, S.; Chen, B.; Hu, B. Simultaneous speciation of inorganic selenium and tellurium in environmental water samples by polyaniline functionalized magnetic solid phase extraction coupled with ICP-MS detection. *Talanta* **2020**, *207*, 120314, doi:10.1016/j.talanta.2019.120314.
287. Li, X.; Li, B.; Chen, M.; Yan, M.; Cao, X.; Yin, J.; Zhang, Z. Preparation of magnetic zeolitic imidazolate framework-8 for magnetic solid-phase extraction of strobilurin fungicides from environmental water samples. *Analytical Methods* **2021**, *13*, 2943-2950, doi:10.1039/D1AY00645B.
288. Zhao, Y.; Wu, R.; Yu, H.; Li, J.; Liu, L.; Wang, S.; Chen, X.; Chan, T.D. Magnetic solid-phase extraction of sulfonamide antibiotics in water and animal-derived food samples using core-shell magnetite and molybdenum disulfide nanocomposite adsorbent. *J. Chromatogr. A* **2020**, *1610*, 460543, doi:10.1016/j.chroma.2019.460543.
289. Senosy, I.A.; Guo, H.M.; Ouyang, M.N.; Lu, Z.H.; Yang, Z.H.; Li, J.H. Magnetic solid-phase extraction based on nano-zeolite imidazolate framework-8-functionalized magnetic graphene oxide for the quantification of residual fungicides in water, honey and fruit juices. *Food Chem.* **2020**, *325*, 126944, doi:10.1016/j.foodchem.2020.126944.
290. Ruan, X.; Xing, L.; Peng, J.; Li, S.; Song, Y.; Sun, Q. A simplified fabric phase sorptive extraction method for the determination of amphetamine drugs in water samples using liquid chromatography-mass spectrometry. *RSC Advances* **2020**, *10*, 10854-10866, doi:10.1039/C9RA10138A.
291. Jiménez-Holgado, C.; Chrimatopoulos, C.; Stathopoulos, V.; Sakkas, V. Investigating the Utility of Fabric Phase Sorptive Extraction and HPLC-UV-Vis/DAD to Determine Antidepressant Drugs in Environmental Aqueous Samples. *Separations* **2020**, *7*, doi:10.3390/separations7030039.
292. Santana-Viera, S.; Guedes-Alonso, R.; Sosa-Ferrera, Z.; Santana-Rodríguez, J.J.; Kabir, A.; Furton, K.G. Optimization and application of fabric phase sorptive extraction coupled to ultra-high performance liquid chromatography tandem mass spectrometry for the determination of cytostatic drug residues in environmental waters. *J. Chromatogr. A* **2017**, *1529*, 39-49, doi:10.1016/j.chroma.2017.10.070.
293. Kalaboka, M.; Chrimatopoulos, C.; Jiménez-Holgado, C.; Boti, V.; Sakkas, V.; Albanis, T. Exploring the Efficiency of UHPLC-Orbitrap MS for the Determination of 20 Pharmaceuticals and Acesulfame K in Hospital and Urban Wastewaters with the Aid of FPSE. *Separations* **2020**, *7*, doi:10.3390/separations7030046.
294. Celeiro, M.; Vazquez, L.; Nurerk, P.; Kabir, A.; Furton, K.G.; Dagnac, T.; Llompart, M. Fabric phase sorptive extraction for the determination of 17 multiclass fungicides in environmental water by gas chromatography-tandem mass spectrometry. *J. Sep. Sci.* **2020**, *43*, 1817-1829, doi:10.1002/jssc.201901232.
295. Gülle, S.; Ulusoy, H.I.; Kabir, A.; Tartaglia, A.; Furton, K.G.; Locatelli, M.; Samanidou, V.F. Application of a fabric phase sorptive extraction-high performance liquid chromatography-photodiode array detection method for the trace determination of methyl paraben, propyl paraben and butyl paraben in cosmetic and environmental samples. *Analytical Methods* **2019**, *11*, 6136-6145, doi:10.1039/C9AY02260K.
296. Sun, T.; Wang, D.; Tang, Y.; Xing, X.; Zhuang, J.; Cheng, J.; Du, Z. Fabric-phase sorptive extraction coupled with ion mobility spectrometry for on-site rapid detection of PAHs in aquatic environment. *Talanta* **2019**, *195*, 109-116, doi:10.1016/j.talanta.2018.11.018.

297. Ulusoy, H.İ.; Köseoğlu, K.; Kabir, A.; Ulusoy, S.; Locatelli, M. Fabric phase sorptive extraction followed by HPLC-PDA detection for the monitoring of pirimicarb and fenitrothion pesticide residues. *Microchimica Acta* **2020**, *187*, 337, doi:10.1007/s00604-020-04306-7.
298. Kabir, A.; Mesa, R.; Jurmain, J.; Furton, K.G. Fabric Phase Sorptive Extraction Explained. *Separations* **2017**, *4*, 21, doi:10.3390/separations4020021.
299. Celeiro, M.; Acerbi, R.; Kabir, A.; Furton, K.G.; Llompart, M. Development of an analytical methodology based on fabric phase sorptive extraction followed by gas chromatography-tandem mass spectrometry to determine UV filters in environmental and recreational waters. *Anal Chim Acta X* **2020**, *4*, 100038, doi:10.1016/j.acax.2019.100038.
300. Anthemidis, A.; Kazantzi, V.; Samanidou, V.; Kabir, A.; Furton, K.G. An automated flow injection system for metal determination by flame atomic absorption spectrometry involving on-line fabric disk sorptive extraction technique. *Talanta* **2016**, *156-157*, 64-70, doi:10.1016/j.talanta.2016.05.012.
301. Huang, G.; Dong, S.; Zhang, M.; Zhang, H.; Huang, T. Fabric phase sorptive extraction: Two practical sample pretreatment techniques for brominated flame retardants in water. *Water Res.* **2016**, *101*, 547-554, doi:10.1016/j.watres.2016.06.007.
302. Aznar, M.; Alfaro, P.; Nerin, C.; Kabir, A.; Furton, K.G. Fabric phase sorptive extraction: An innovative sample preparation approach applied to the analysis of specific migration from food packaging. *Anal. Chim. Acta* **2016**, *936*, 97-107, doi:10.1016/j.aca.2016.06.049.
303. Alcudia-León, M.C.; Lucena, R.; Cárdenas, S.; Valcárcel, M.; Kabir, A.; Furton, K.G. Integrated sampling and analysis unit for the determination of sexual pheromones in environmental air using fabric phase sorptive extraction and headspace-gas chromatography-mass spectrometry. *J. Chromatogr. A* **2017**, *1488*, 17-25, doi:10.1016/j.chroma.2017.01.077.
304. Tang, W.; Dai, Y.; Row, K.H. Evaluation of fatty acid/alcohol-based hydrophobic deep eutectic solvents as media for extracting antibiotics from environmental water. *Anal. Bioanal. Chem.* **2018**, *410*, 7325-7336, doi:10.1007/s00216-018-1346-6.
305. Werner, J. Low Density Ionic Liquid-Based Ultrasound-Assisted Dispersive Liquid–Liquid Microextraction for the Preconcentration of Trace Aromatic Amines in Waters. *J. Anal. Chem.* **2021**, *76*, 1182-1188, doi:10.1134/S1061934821100130.
306. Yang, S.; Liu, H.; Hu, K.; Deng, Q.; Wen, X. Investigation of thermospray flame furnace atomic absorption spectrometric determination of cadmium combined with ultrasound-assisted dispersive liquid-liquid microextraction. *Int. J. Environ. Anal. Chem.* **2022**, *102*, 443-455, doi:10.1080/03067319.2020.1723566.
307. Ali, J.; Tuzen, M.; Citak, D.; Uluozlu, O.D.; Mendil, D.; Kazi, T.G.; Afridi, H.I. Separation and preconcentration of trivalent chromium in environmental waters by using deep eutectic solvent with ultrasound-assisted based dispersive liquid-liquid microextraction method. *J. Mol. Liq.* **2019**, *291*, 111299, doi:10.1016/j.molliq.2019.111299.
308. Ayala-Cabrera, J.F.; Trujillo-Rodríguez, M.J.; Pino, V.; Hernández-Torres, Ó.M.; Afonso, A.M.; Sirieix-Plénet, J. Ionic liquids versus ionic liquid-based surfactants in dispersive liquid–liquid microextraction for determining copper in water by flame atomic absorption spectrometry. *Int. J. Environ. Anal. Chem.* **2016**, *96*, 101-118, doi:10.1080/03067319.2015.1128538.
309. Shojaei, S.; Shojaei, S.; Nouri, A.; Baharinikoo, L. Application of chemometrics for modeling and optimization of ultrasound-assisted dispersive liquid–liquid microextraction for the simultaneous determination of dyes. *npj Clean Water* **2021**, *4*, 23, doi:10.1038/s41545-021-00113-6.
310. Dias, R.A.S.; Sousa, E.R.; Silva, G.S.; Silva, L.K.; Freitas, A.S.; Lima, D.L.D.; Sousa, E.M.L. Ultrasound-assisted dispersive liquid-liquid microextraction for determination of enrofloxacin in surface waters. *Microchem. J.* **2021**, *160*, 105633, doi:10.1016/j.microc.2020.105633.
311. Xizhi, S.; Sun, A.-l.; Wang, Q.-h.; Hengel, M.; Shibamoto, T. Rapid Multi-Residue Analysis of Herbicides with Endocrine-Disrupting Properties in Environmental Water Samples Using Ultrasound-Assisted Dispersive Liquid–Liquid Microextraction and Gas Chromatography–Mass Spectrometry. *Chromatographia* **2018**, *81*, doi:10.1007/s10337-018-3530-4.
312. Chisvert, A.; Benedé, J.L.; Anderson, J.L.; Pierson, S.A.; Salvador, A. Introducing a new and rapid microextraction approach based on magnetic ionic liquids: Stir bar dispersive liquid microextraction. *Anal. Chim. Acta* **2017**, *983*, 130-140, doi:10.1016/j.aca.2017.06.024.
313. Werner, J. Ionic liquid ultrasound-assisted dispersive liquid-liquid microextraction based on solidification of the aqueous phase for preconcentration of heavy metals ions prior to determination by LC-UV. *Talanta* **2018**, *182*, 69-73, doi:10.1016/j.talanta.2018.01.060.
314. Abdi, K.; Ezoddin, M.; Pirooznia, N. Ultrasound-assisted liquid–liquid microextraction based on solidification of floating organic droplet using deep eutectic solvent as disperser for preconcentration of Ni and Co. *Int. J. Environ. Anal. Chem.* **2021**, 10.1080/03067319.2021.1931853, 1-14, doi:10.1080/03067319.2021.1931853.
315. Benedé, J.L.; Anderson, J.L.; Chisvert, A. Trace determination of volatile polycyclic aromatic hydrocarbons in natural waters by magnetic ionic liquid-based stir bar dispersive liquid microextraction. *Talanta* **2018**, *176*, 253-261, doi:10.1016/j.talanta.2017.07.091.
316. Carbonell-Rozas, L.; Canales, R.; Lara, F.J.; García-Campaña, A.M.; Silva, M.F. A natural deep eutectic solvent as a novel dispersive solvent in dispersive liquid-liquid microextraction based on solidification of floating organic droplet for the determination of pesticide residues. *Anal. Bioanal. Chem.* **2021**, *413*, 6413-6424, doi:10.1007/s00216-021-03605-z.

317. Wang, X.M.; Du, T.T.; Wang, J.; Kou, H.X.; Du, X.Z. Determination of polybrominated biphenyls in environmental water samples by ultrasound-assisted dispersive liquid-liquid microextraction followed by high-performance liquid chromatography. *Microchem. J.* **2019**, *148*, 85-91, doi:10.1016/j.microc.2019.04.065.
318. Xinya, L.; Liu, C.; Qian, H.; Qu, Y.; Zhang, S.; Lu, R.; Gao, H.; Zhou, W. Ultrasound-assisted dispersive liquid-liquid microextraction based on a hydrophobic deep eutectic solvent for the preconcentration of pyrethroid insecticides prior to determination by high-performance liquid chromatography. *Microchem. J.* **2019**, *146*, doi:10.1016/j.microc.2019.01.048.
319. Rahimi Moghadam, M.; Zargar, B.; Rastegarzadeh, S. Determination of Tetracycline Using Ultrasound-Assisted Dispersive Liquid-Liquid Microextraction Based on Solidification of Floating Organic Droplet Followed by HPLC-UV System. *J. AOAC Int.* **2021**, *104*, 999-1004, doi:10.1093/jaoacint/qsab044.
320. El-Deen, A.K.; Shimizu, K. Deep eutectic solvent as a novel disperser in dispersive liquid-liquid microextraction based on solidification of floating organic droplet (DLLME-SFOD) for preconcentration of steroids in water samples: Assessment of the method deleterious impact on the e. *Microchem. J.* **2019**, *149*, 103988-103988, doi:10.1016/j.microc.2019.103988.
321. Moslemzadeh, M.; Larki, A.; Ghanemi, K. A combination of dispersive liquid–liquid microextraction and smartphone-based colorimetric system for the phenol measurement. *Microchem. J.* **2020**, *159*, 105583, doi:10.1016/j.microc.2020.105583.
322. Townsend, R.; Keulen, G.; Desbrow, C.; Godfrey, A.R. An investigation of the utility of QuEChERS for extracting acid, base, neutral and amphiphilic species from example environmental and clinical matrices. *Analytical Science Advances* **2020**, *1*, 152-160, doi:10.1002/ansa.202000018.
323. Hrynko, I.; Łozowicka, B.; Kaczyński, P. Development of precise micro analytical tool to identify potential insecticide hazards to bees in guttation fluid using LC-ESI-MS/MS. *Chemosphere* **2021**, *263*, 128143, doi:10.1016/j.chemosphere.2020.128143.
324. Stoeckelhuber, M.; Müller, C.; Vetter, F.; Mingo, V.; Lötters, S.; Wagner, N.; Bracher, F. Determination of Pesticides Adsorbed on Arthropods and Gastropods by a Micro-QuEChERS Approach and GC–MS/MS. *Chromatographia* **2017**, *80*, doi:10.1007/s10337-017-3280-8.
325. Moreno-Gonzalez, D.; Alcantara-Duran, J.; Gilbert-Lopez, B.; Beneito-Cambra, M.; Cutillas, V.M.; Rajski, L.; Molina-Diaz, A.; Garcia-Reyes, J.F. Sensitive Detection of Neonicotinoid Insecticides and Other Selected Pesticides in Pollen and Nectar Using Nanoflow Liquid Chromatography Orbitrap Tandem Mass Spectrometry. *J. AOAC Int.* **2017**, 10.5740/jaoacint.17-0412, doi:10.5740/jaoacint.17-0412.
326. Kurth, D.; Krauss, M.; Schulze, T.; Brack, W. Measuring the internal concentration of volatile organic compounds in small organisms using micro-QuEChERS coupled to LVI-GC-MS/MS. *Anal. Bioanal. Chem.* **2017**, *409*, 6041-6052, doi:10.1007/s00216-017-0532-2.
327. An, J.; Rahn, K.L.; Anderson, J.L. Headspace single drop microextraction versus dispersive liquid-liquid microextraction using magnetic ionic liquid extraction solvents. *Talanta* **2017**, *167*, 268-278, doi:10.1016/j.talanta.2017.01.079.
328. Mafra, G.; Vieira, A.A.; Merib, J.; Anderson, J.L.; Carasek, E. Single drop microextraction in a 96-well plate format: A step toward automated and high-throughput analysis. *Anal. Chim. Acta* **2019**, *1063*, 159-166, doi:10.1016/j.aca.2019.02.013.
329. Nunes, L.S.; das Graças Andrade Korn, M.; Lemos, V.A. Direct Immersion Single-Drop Microextraction and Continuous-Flow Microextraction for the Determination of Manganese in Tonic Drinks and Seafood Samples. *Food Analytical Methods* **2020**, *13*, 1681-1689, doi:10.1007/s12161-020-01794-4.
330. Mehravar, A.; Feizbakhsh, A.; Sarafi, A.H.M.; Konoz, E.; Faraji, H. Deep eutectic solvent-based headspace single-drop microextraction of polycyclic aromatic hydrocarbons in aqueous samples. *J. Chromatogr. A* **2020**, *1632*, 461618, doi:10.1016/j.chroma.2020.461618.
331. Pano-Farias, N.S.; Ceballos-Magaña, S.G.; Muñiz-Valencia, R.; Jurado, J.M.; Alcázar, Á.; Aguayo-Villarreal, I.A. Direct immersion single drop micro-extraction method for multi-class pesticides analysis in mango using GC-MS. *Food Chem.* **2017**, *237*, 30-38, doi:10.1016/j.foodchem.2017.05.030.
332. Kiszkiel-Taudul, I.; Starczewska, B. Single drop microextraction coupled with liquid chromatography-tandem mass spectrometry (SDME-LC-MS/MS) for determination of ranitidine in water samples. *Microchem. J.* **2019**, *145*, 936-941, doi:10.1016/j.microc.2018.12.015.
333. Nunes, L.S.; Korn, M.G.A.; Lemos, V.A. A novel direct-immersion single-drop microextraction combined with digital colorimetry applied to the determination of vanadium in water. *Talanta* **2021**, *224*, 121893, doi:10.1016/j.talanta.2020.121893.
334. Yousefi, S.M.; Shemirani, F.; Ghorbanian, S.A. Enhanced headspace single drop microextraction method using deep eutectic solvent based magnetic bucky gels: Application to the determination of volatile aromatic hydrocarbons in water and urine samples. *J. Sep. Sci.* **2018**, *41*, 966-974, doi:10.1002/jssc.201700807.
335. Kannouma, R.E.; Hammad, M.A.; Kamal, A.H.; Mansour, F.R. A dispersive liquid–liquid microextraction method based on solidification of floating organic droplet for determination of antiviral agents in environmental water using HPLC/UV. *Microchem. J.* **2021**, *171*, 106790, doi:10.1016/j.microc.2021.106790.
336. Aydın Urucu, O.; Dönmez, Ş.; Kök Yetimoğlu, E. Solidified Floating Organic Drop Microextraction for the Detection of Trace Amount of Lead in Various Samples by Electrothermal Atomic Absorption Spectrometry. *Journal of Analytical Methods in Chemistry* **2017**, *2017*, 6268975, doi:10.1155/2017/6268975.

337. de S. Dias, F.; de S. R. Neto, S.C.; de N. Pires, L.; Lemos, V.A. Emulsification solidified floating organic drop microextraction assisted by ultrasound for the determination of nickel, cobalt and copper in oyster and fish samples. *Analytical Methods* **2020**, *12*, 865-871, doi:10.1039/C9AY02453K.
338. Silva, L.K.; Rangel, J.H.G.; Brito, N.M.; Sousa, E.R.; Sousa É, M.L.; Lima, D.L.D.; Esteves, V.I.; Freitas, A.S.; Silva, G.S. Solidified floating organic drop microextraction (SFODME) for the simultaneous analysis of three non-steroidal anti-inflammatory drugs in aqueous samples by HPLC. *Anal. Bioanal. Chem.* **2021**, *413*, 1851-1859, doi:10.1007/s00216-021-03153-6.
339. Shiri, S. Salting out and vortex-assisted dispersive liquid–liquid microextraction based on solidification of floating organic drop microextraction as a new approach for simultaneous determination of phenol and chlorophenols in water samples. **2019**, 10.30473/icc.2019.47906.1592, doi:10.30473/icc.2019.47906.1592.
340. Zhang, K.; Li, S.; Wang, Y.; Fan, J.; Zhu, G. Air-assisted liquid-liquid microextraction based on solidification of floating deep eutectic solvent for the analysis of ultraviolet filters in water samples by high performance liquid chromatography with the aid of response surface methodology. *J. Chromatogr. A* **2020**, *1618*, 460876, doi:10.1016/j.chroma.2020.460876.
341. Fayaz, S.M.; Abdoli, M.A.; Baghdadi, M.; Karbasi, A. Ag removal from e-waste using supercritical fluid: improving efficiency and selectivity. *Int. J. Environ. Stud.* **2021**, *78*, 459-473, doi:10.1080/00207233.2020.1834305.
342. Nakamura, K.; Otake, T.; Hanari, N. Evaluation of supercritical fluid extraction for the determination of neonicotinoid pesticides in green onion. *Journal of Environmental Science and Health, Part B* **2020**, *55*, 604-612, doi:10.1080/03601234.2020.1747905.
343. Wicker, A.P.; Carlton, D.D., Jr.; Tanaka, K.; Nishimura, M.; Chen, V.; Ogura, T.; Hedgepeth, W.; Schug, K.A. On-line supercritical fluid extraction-supercritical fluid chromatography-mass spectrometry of polycyclic aromatic hydrocarbons in soil. *J. Chromatogr. B Analyt. Technol. Biomed. Life. Sci.* **2018**, *1086*, 82-88, doi:10.1016/j.jchromb.2018.04.014.
344. Falsafi, Z.; Raofie, F.; Kazemi, H.; Ariya, P.A. Simultaneous extraction and fractionation of petroleum biomarkers from tar balls and crude oils using a two-step sequential supercritical fluid extraction. *Mar. Pollut. Bull.* **2020**, *159*, 111484, doi:10.1016/j.marpolbul.2020.111484.
345. Meskar, M.; Sartaj, M.; Infante Sedano, J.A. Assessment and comparison of PHCs removal from three types of soils (sand, silt loam and clay) using supercritical fluid extraction. *Environ. Technol.* **2019**, *40*, 3040-3053, doi:10.1080/09593330.2018.1466917.
346. Yao, Y.; Chau, E.; Azimi, G. Supercritical fluid extraction for purification of waxes derived from polyethylene and polypropylene plastics. *Waste Manag* **2019**, *97*, 131-139, doi:10.1016/j.wasman.2019.08.003.
347. Tita, G.J.; Navarrete, A.; Martin, A.; Cocero, M.J. Model assisted supercritical fluid extraction and fractionation of added-value products from tobacco scrap. *J. Supercrit. Fluids* **2021**, *167*, 105046, doi:10.1016/j.supflu.2020.105046.
348. Lie, J.; Tanda, S.; Liu, J.-C. Subcritical Water Extraction of Valuable Metals from Spent Lithium-Ion Batteries. *Molecules* **2020**, *25*, doi:10.3390/molecules25092166.
349. Taki, G.; Islam, M.N.; Park, S.-J.; Park, J.-H. Optimization of operating parameters to remove and recover crude oil from contaminated soil using subcritical water extraction process. *Environmental Engineering Research* **2018**, *23*, 175-180, doi:10.4491/eer.2017.145.
350. Kang, S.J.; Sun, Y.H.; Qiao, M.Y.; Li, S.L.; Deng, S.H.; Guo, W.; Li, J.S.; He, W.T. The enhancement on oil shale extraction of FeCl3 catalyst in subcritical water. *Energy* **2022**, *238*, 121763, doi:10.1016/j.energy.2021.121763.
351. Ramos-Contreras, C.; Concha-Grana, E.; Lopez-Mahia, P.; Molina-Perez, F.; Muniategui-Lorenzo, S. Determination of atmospheric particle-bound polycyclic aromatic hydrocarbons using subcritical water extraction coupled with membrane microextraction. *J. Chromatogr. A* **2019**, *1606*, 460381, doi:10.1016/j.chroma.2019.460381.
352. Islam, M.; Jung, S.-K.; Jung, H.-Y.; Park, J.-H. The feasibility of recovering oil from contaminated soil at petroleum oil spill site using a subcritical water extraction technology. *Process Saf. Environ. Prot.* **2017**, *111*, doi:10.1016/j.psep.2017.06.015.
353. Zohar, M.; Matzrafi, M.; Abu-Nassar, J.; Khoury, O.; Gaur, R.Z.; Posmanik, R. Subcritical water extraction as a circular economy approach to recover energy and agrochemicals from sewage sludge. *J. Environ. Manage.* **2021**, *285*, 112111, doi:10.1016/j.jenvman.2021.112111.
